# Iron-Catalyzed Thioarylation of Arenes Using Saccharin-Derived Reagents

**DOI:** 10.1021/acs.joc.5c00250

**Published:** 2025-04-17

**Authors:** Lachlan J. N. Waddell, Oluwajuwon A. M. Okunade, Amy C. Dodds, Michael C. H. Lok, R. Nisha Khanizeman, Andrew Sutherland

**Affiliations:** † School of Chemistry, University of Glasgow, The Joseph Black Building, Glasgow G12 8QQ, U.K.; ‡ GSK Medicines Research Centre, Gunnels Wood Road, Stevenage SG1 2NY, U.K.

## Abstract

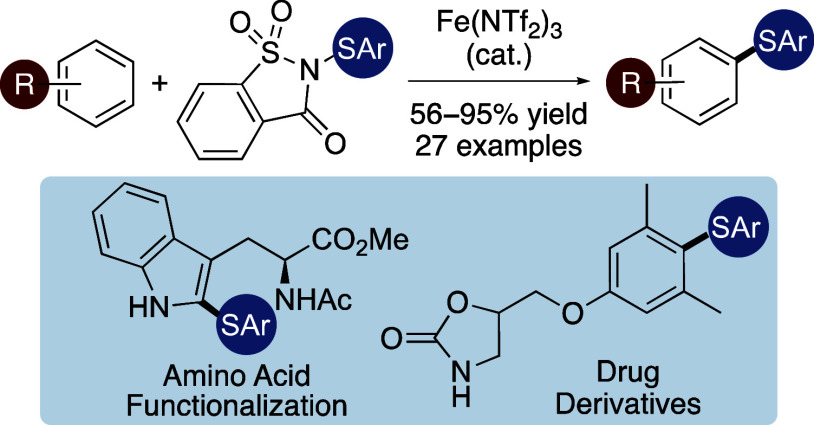

Biaryl sulfides are important scaffolds found in various natural products and pharmaceutically active compounds. One of the main approaches for the synthesis of this compound class involves the substitution of arenes using electrophilic thioaryl species. However, these methods generally require acidic activation of the electrophile, more forcing conditions, and long reaction times. Here, we describe the combination of the super Lewis acid iron(III) triflimide with saccharin-based thioarylation reagents for the rapid synthesis of unsymmetrical biaryl sulfides under mild conditions. This approach was effective with electron-deficient thioaryl species that performed poorly with previous methods, allowing the efficient functionalization of bioactive compounds.

## Introduction

**Figure 1:**
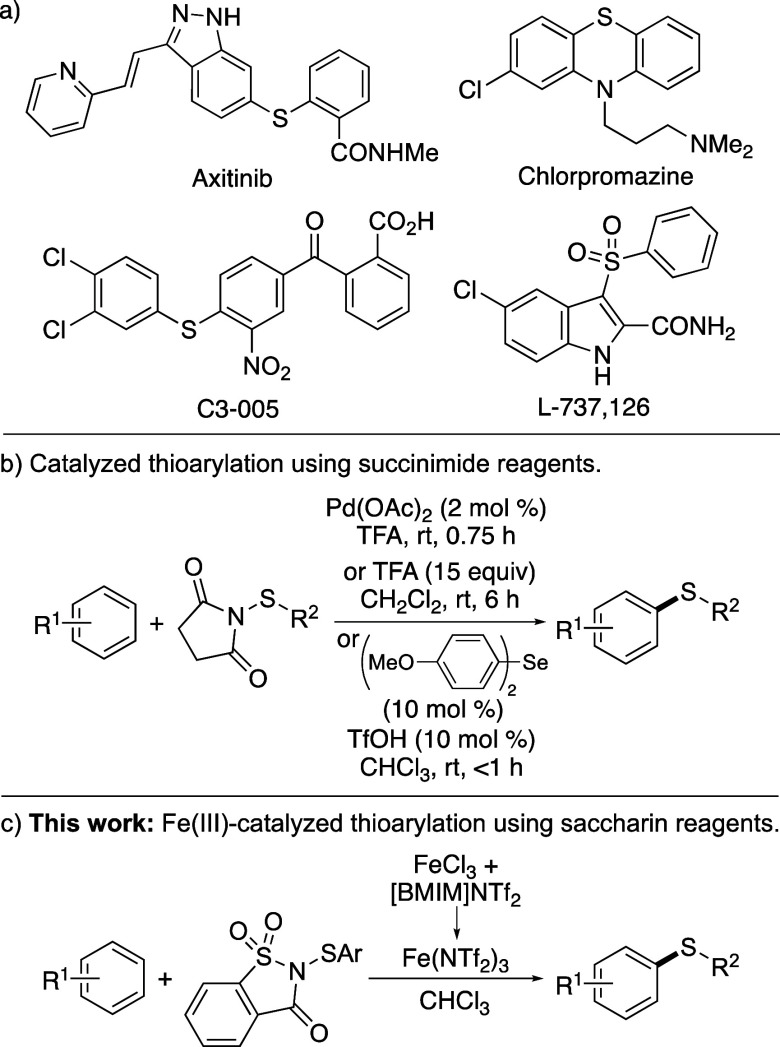
(a) Examples of bioactive aryl thioethers. (b) Previous thioarylation reactions using succinimide reagents. (c) This work: thioarylation using saccharin-based reagents.

Polyaromatic compounds containing sulfur, such as biaryl thioethers, are important motifs for organic chemistry. In synthesis, they serve as intermediates for sulfoxides and sulfones, while in medicinal chemistry, aryl thioethers are prevalent in many pharmaceutical agents.^1^ Examples include axitinib, a tyrosine kinase inhibitor,^2^ phenothiazines such as chlorpromazine,^3^ which are employed as antipsychotic drugs, and C3–005, an antimicrobial agent that targets the protein–protein interaction between bacterial RNA polymerase and the sigma initiation factor (Figure 1a).^4^ Many sulfone-containing drugs such as the HIV-1 reverse transcriptase inhibitor L-737,126 are prepared via an aryl thioether intermediate with C–S bond formation as the key step.^5^

Due to this importance, many strategies have been developed for the synthesis of biaryl thioethers. Traditional methods include the reaction of organometallic reagents with electrophilic arylsulfur compounds^6^ or the transition metal-catalyzed cross-coupling of aryl (pseudo)halides with thiols or disulfides.^7^ Biaryl sulfides have also been prepared by direct thiolation of aryl C–H bonds,^8^ including a method reported by Procter and co-workers that utilizes thioarylation of arenes using electrophilic sulfoxonium salts.^8b^ Thioarylation of arenes has also been achieved using *N*-(arylthio)succinimides as electrophilic reagents (Figure 1b).^9−12^ These transformations have been facilitated by palladium catalysis, necessitating the use of trifluoroacetic acid (TFA) as the solvent.^9^ Alternative approaches have used Brønsted acids such as TFA^11^ or triflic acid to activate the succinimide reagent.^12^ These include accelerated reactions reported by Gustafson and co-workers, who demonstrated that a dual-catalytic process using a combination of triflic acid and biaryl selenides as Lewis bases allowed fast thiolation of electron-rich arenes and heteroarenes.^12^

To avoid the use of Brønsted acids such as TFA or triflic acid, we recently developed a thioarylation reaction of activated arenes using the super Lewis acid, iron(III) triflimide.^13^ In exploring the scope of this process, it was found that a wide range of arenes could undergo accelerated thioarylation under relatively mild conditions, resulting in the application of this reaction for the synthesis of the leprosy drug, dapsone, and the antidepressant, vortioxetine. However, despite the wide scope of this process, limitations were observed. For example, while electron-rich thioaryl groups could be transferred efficiently under mild conditions, electron-deficient analogues required higher temperatures (up to 75 °C) and longer reaction times (up to 48 h).^13a^ Mechanistic studies demonstrated that on formation of electron-rich biaryl thioethers, these acted as Lewis base catalysts, accelerating the activation of remaining *N*-(arylthio)succinimide, while electron-deficient biaryl thioethers were formed solely via the slower iron(III) activation pathway. More recently, we have reported the use of iron(III) salts as Lewis acids for the thiocyanation and trifluoromethylthiolation of arenes.^14,15^ During the optimization of these processes, it was found that saccharin-based reagents, which are more effective leaving groups, resulted in faster and more efficient transformations compared to succinimide reagents. Based on this work, we proposed that *N*-(arylthio)saccharin reagents may act as more effective reagents for the iron(III) triflimide-catalyzed synthesis of biaryl thioethers and, in particular, allow faster transfer of electron-deficient thioaryl groups under mild conditions. Here, we report the application of *N*-(arylthio)saccharin reagents for the synthesis of biaryl thioethers and demonstrate the advantages of these compared to analogous succinimide-based reagents (Figure 1c). We also show the use of these reagents for the transfer of electron-deficient thioaryl groups, such as chloro- and nitro-substituted thioarenes, and demonstrate the synthetic utility of this process with the thioarylation of bioactive compounds.

## Results and Discussion

The development of a faster thioarylation reaction under milder conditions using electron-deficient thioaryl groups was investigated with the synthesis of (4′-chlorophenyl)(4-methoxyphenyl)sulfane (**2a**) (Table 1). Previously, the reaction of anisole (**1a**) with *N*-(4-chlorophenylthio)succinimide (**3a**) in the presence of iron(III) triflimide (2.5 mol %), which is prepared in situ from iron(III) chloride and the readily available [BMIM]NTf_2_ ionic liquid gave biaryl thioether **2a** in a 90% yield (entry 1).^13a,16^ To achieve full conversion, a temperature of 60 °C and a reaction time of 68 h were required. Gustafson and co-workers demonstrated that Brønsted acid-catalyzed thioarylation reactions using *N*-(arylthio)succinimides could be accelerated using Lewis bases such as biaryl selenides.^12^ It is proposed that on Brønsted acid activation of the succinimide reagents, the biaryl selenide forms a cationic intermediate with the thioaryl group that then quickly reacts with the arene (quod vide). Based on this, we investigated the use of the Lewis base diphenyl selenide in combination with iron(III) triflimide to accelerate the synthesis of **2a**. Thus, diphenyl selenide (**4**) (10 mol %) was added to the iron(III) triflimide-catalyzed reaction of anisole (**1a**) with succinimide **3a** (entry 2). Although this had the desired effect of accelerating the reaction (4 h) while maintaining a high yield of **2a** (95%), a temperature of 60 °C was still required. The analogous saccharin-derived reagent **5a** was next investigated. In general, *N*-(arylthio)saccharin reagents are typically prepared by deprotonation of saccharin with sodium hydride, followed by a reaction with an arylsulfenyl chloride.^17^ However, as the commercial availability of arylsulfenyl chlorides is limited, this requires the synthesis of these reagents. For this project, we developed a straightforward method involving the reaction of *N*-chlorosaccharin with thiophenols in the presence of triethylamine. This gave the corresponding *N*-(arylthio)saccharin reagents in 70–75% yields (see experimental section). Using saccharin-derived reagent **5a** at 60 °C, the reaction with **1a** was complete in 5 min and gave biaryl thioether **2a** in a 93% yield (entry 3). In our previous work on iron(III)-catalyzed thiocyanation of arenes, we found that the smaller Lewis acid, iron(III) chloride, was more effective at activating *N*-thiocyanatosaccharin.^14^ In this current study, the use of only iron(III) chloride (2.5 mol %) for the activation of **5a** gave biaryl sulfide **2a** in a lower yield of 77% and required a longer reaction time of 0.5 h (entry 4). Using iron(III) triflimide as the preferred Lewis acid, the limits of the reaction temperature were next studied. At 40 °C, this gave similar results (entry 5), while at room temperature (20 °C, entry 6), **2a** was formed in a 94% yield after a reaction time of 0.5 h. This final set of conditions was deemed optimal and used to investigate a larger-scale reaction. On a 1 mmol scale, the reaction was also complete after 0.5 h and gave a similar yield (90%).

**Table 1:**
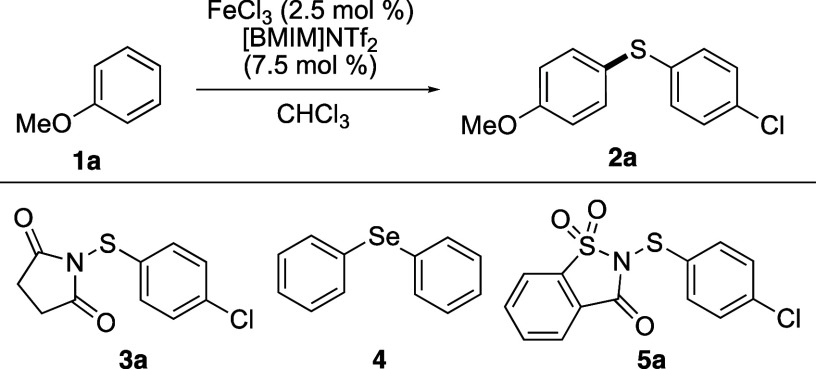
Optimization Studies for Fe(III)-Catalyzed Synthesis of Bis(4-methoxyphenyl)sulfane (2a)

entry	reagent	temperature (°C)	time (h)	yield (%)^a^
1	**3a**	60	68	90
2^b^	**3a** + **4**	60	4	95
3	**5a**	60	0.1	93
4^c^	**5a**	60	0.5	77
5	**5a**	40	0.1	92
6	**5a**	20	0.5	94

a Isolated yields.

b Using diphenyl selenide (10 mol %).

c Using only FeCl_3_ (2.5 mol %). [BMIM]NTf_2_ was omitted from this reaction.

**Scheme 1:**
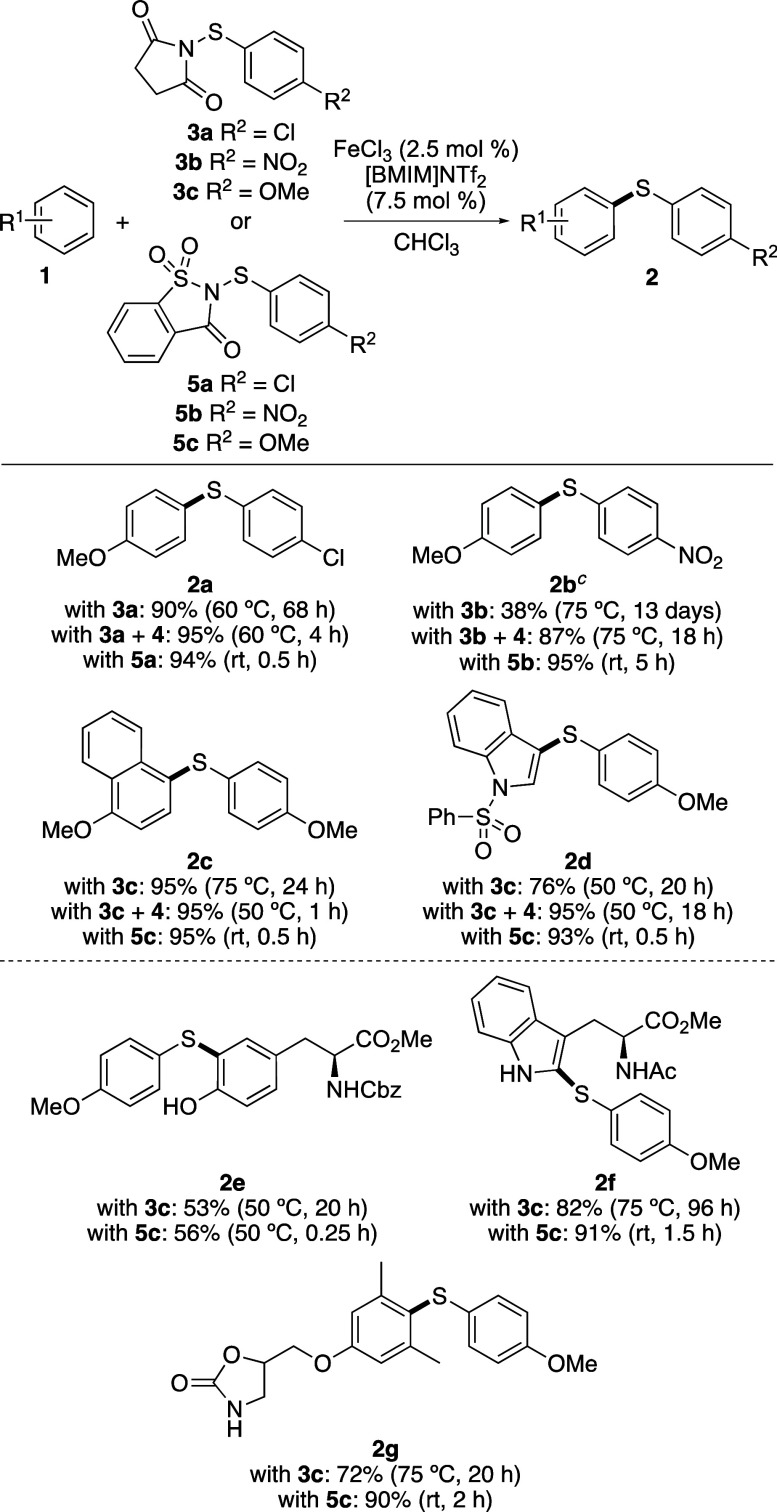
Comparison of *N*-(Arylthio)succinimide and *N*-(Arylthio)saccharin Reagents^,^ Isolated yields. For reactions with diphenyl selenide (**4**), 10 mol % was used. All reactions used Fe(NTf_2_)_3_ (10 mol %).

Following the identification of optimized conditions, a study was next conducted to compare the use of saccharin reagents with that of analogous succinimide compounds (Scheme 1). In particular, targets from our previous study that required higher temperatures and long reaction times with *N*-(arylthio)succinimides were investigated.^13a^ As found for the synthesis of **2a**, the preparation of biaryl thioethers **2b**–**2d** all showed improvement using the dual catalytic process of Fe(NTf_2_)_3_ and diphenyl selenide (**4**) with succinimide reagents. In general, elevated temperatures were still required; however, the reaction times were reduced. In all examples, both the temperature and reaction time were significantly reduced using the corresponding saccharin reagent. For example, the use of Fe(NTf_2_)_3_ and succinimide **3b** for the synthesis of (4-methoxyphenyl)(4′-nitrophenyl)sulfane (**2b**) gave the product in a 38% yield and required a temperature of 75 °C and a reaction time of 13 days. Addition of diphenyl selenide (10 mol %) still entailed a temperature of 75 °C, but this led to an improved yield of 87% and a reaction time of 18 h. Using saccharin reagent **5b**, the reaction was found to proceed at room temperature and was complete after 5 h, resulting in a 95% yield of biaryl thioether **2b**. As well as relatively simple arenes, the iron(III) triflimide-catalyzed thioarylation using saccharin reagents with more complex substrates such as the amino acids, tyrosine and tryptophan, as well as the muscle relaxant, metaxalone, were also investigated.^18^ Compared to the use of succinimide reagent **3c**, the iron(III)-catalyzed reaction of *N*-Cbz-l-tyrosine methyl ester with *N*-(arylthio)saccharin **5c** still required a temperature of 50 °C, but an accelerated reaction was observed (15 min vs 20 h). For both *N*-acetyl-l-tryptophan methyl ester and metaxalone, the use of saccharin reagent **5c** allowed significantly faster reactions at room temperature. The reactions were also cleaner with **5c**, resulting in improved yields of both **2f** and **2g**. Overall, the addition of the Lewis base diphenyl selenide to iron(III) triflimide-catalyzed reactions with succinimide reagents led to faster reactions but still required elevated temperatures. In contrast, the use of iron(III) triflimide in combination with saccharin reagents resulted in accelerated reactions at lower temperatures for nearly all of the substrates investigated.

**Scheme 2:**
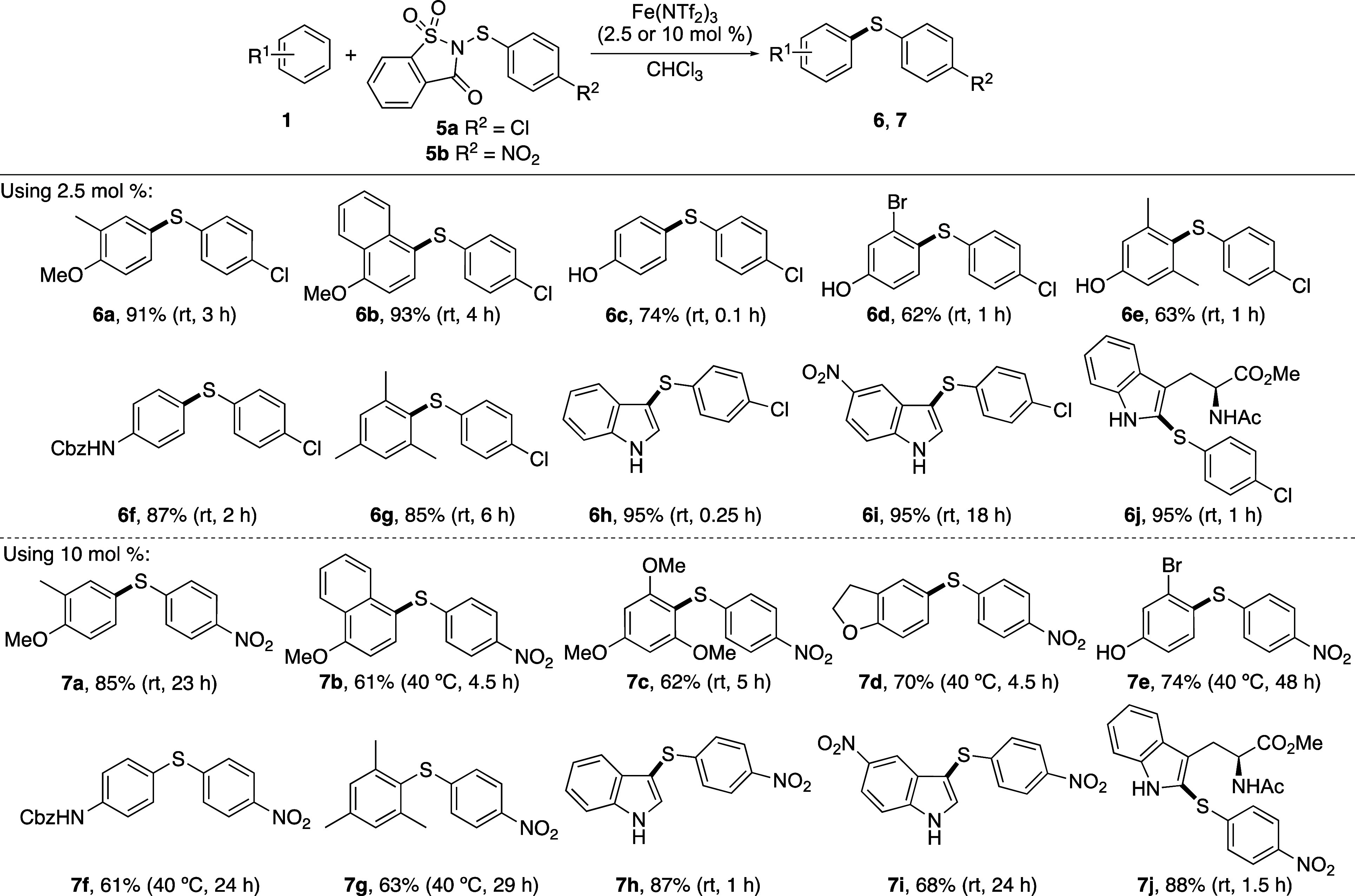
Reaction Scope of Arenes Isolated yields.

One of the key objectives of this project was to overcome the limitations of previous methods and develop a thioarylation reaction that is compatible with the transfer of electron-deficient thioaryl groups. Having identified iron triflimide-catalyzed reactions with saccharin reagents as fast and amenable to mild conditions, the scope of thioarylation using electron-deficient *N*-(arylthio)saccharin reagents **5a** and **5b** was explored using various arenes (Scheme 2). The transfer of the 4-chlorothiophenyl group using **5a** was found to proceed using a low catalyst loading (2.5 mol %) and at room temperature. Under these conditions, the thioarylation of various anisoles, phenols, and Cbz-protected aniline was fast and gave the products (**6a**–**6f**) in good to excellent yields (62–93%). The reaction with the less activated mesitylene required a longer reaction time of 6 h but at room temperature gave the product **6g** in an 85% yield. Thioarylation of indoles was also effective under these conditions and gave products **6h**–**6j** in quantitative yields. As expected, while the reactions with indole and tryptophan were fast (0.25–1 h), the reaction with the less active 5-nitroindole took 18 h to reach completion. For the transfer of the more challenging 4-nitrothiophenyl group using **5b**, a higher catalyst loading (10 mol %), and as commonly observed for less activated arenes, a slightly elevated temperature of 40 °C was necessary. However, iron(III) triflimide-catalyzed thioarylation with anisoles, phenols, protected anilines, mesitylene, and various indoles gave the products in good to excellent yields (61–88%). Of particular note was the room temperature thioarylation of 1,3,5-trimethoxybenzene that despite *ortho*,*ortho*-substituents gave **7c** in a 62% yield after 5 h. In addition, C2-thioarylation of tryptophan with the 4-nitrothiophenyl group was also effective at room temperature and gave **7j** in an 88% yield after 1.5 h. Thus, the combination of iron(III) triflimide-catalyzed activation with more reactive saccharin-derived compounds has overcome the limitations of previous reagents, permitting the rapid transfer of electron-deficient thioaryl groups under mild conditions. Although the use of saccharin-derived reagents in combination with the super Lewis acid iron(III) triflimide has allowed significant advances in reaction conditions and the transfer of electron-deficient aryl thiols, there are still some limitations. For example, this approach still requires activated electron-rich arene substrates. Attempted thioarylation of bromobenzene using **5a** showed no conversion. Similarly, the transfer of alkyl thiols requires further development. In a similar manner to *N*-alkylthiosuccinimides,^13a^ attempted thioalkylation using *N*-alkylthiosaccharin reagents gave low conversions and complex mixtures of products.

**Scheme 3:**
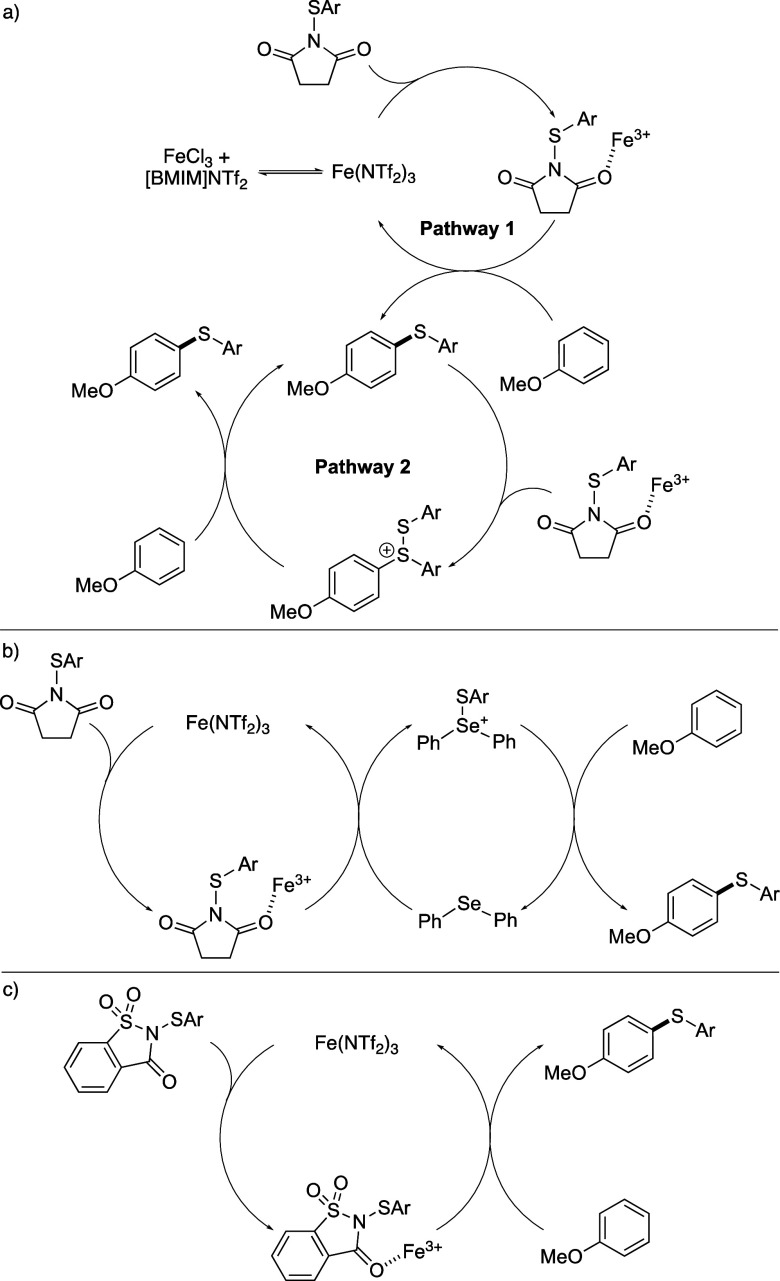
Proposed Reaction Pathways for Iron(III) Triflimide-Catalyzed Thioarylation Reactions

Based on the observed reactivity of the three different iron(III) triflimide-catalyzed thioarylation reactions, reaction pathways have been proposed (Scheme 3). In the original reaction (Scheme 3a),^13a^ ligand exchange between FeCl_3_ and [BMIM]NTf_2_ generates the super Lewis acid, iron(III) triflimide, which activates the succinimide reagent for subsequent reaction with an electron-rich arene, such as anisole (pathway 1).^19^ For thioaryl groups that contain an electron-withdrawing substituent, this is the only pathway in operation and due to electronics is relatively slow. For thioaryl groups that contain an electron-rich substituent, a second pathway becomes available. On initial formation of the thioarylated product via pathway 1, the transformation becomes autocatalytic through the reaction of the biaryl sulfide with the activated *N*-(arylthio)succinimide, resulting in a cationic disulfide intermediate. This can react with the arene, generating the product, and as the cationic disulfide is highly reactive, overall, this gives rise to a significantly faster reaction. These two pathways account for the different reaction conditions required for an electron-deficient product such as **2b** (75 °C and 13 days), and an electron-rich product such as bis(4-methoxyphenyl)sulfane (rt and 2 h).^13a^ When a Lewis base such as diphenyl selenide is added to iron(III) triflimide and an *N*-(arylthio)succinimide reagent, reaction via a thioarylated selenium cation is the main pathway (Scheme 3b). On activation of the *N*-(arylthio)succinimide by iron(III) triflimide, a subsequent reaction with diphenyl selenide forms the selenium cation, and due to the high reactivity of this intermediate, the reaction with an activated arene is fast. As shown by the results in Scheme 1, the combination of a Lewis acid and a Lewis base in a dual catalytic process allows faster reactions than those using iron(III) triflimide. However, the conditions are still highly dependent on the electronics of the transferring thioaryl group, with an electron-rich group notably faster than an electron-deficient group (e.g., **2c** vs **2b**). In contrast to the two previous thioarylation reactions, activation of an *N*-(arylthio)saccharin reagent with only iron(III) triflimide generates an intermediate of suitable reactivity that can undergo a fast thioarylation even during the transfer of electron-deficient thioarenes (Scheme 3c). While this process is still partly influenced by the electronics of the thioaryl group (e.g., **2a** vs **2b**), the dependence is reduced compared with the other processes, permitting faster transfer of electron-deficient thioaryl groups at lower temperatures.

## Conclusions

In conclusion, iron(III) triflimide-catalyzed activation of *N*-(arylthio)saccharin reagents and subsequent reaction with arenes have resulted in a new procedure for the regioselective synthesis of unsymmetrical biaryl sulfides. Previous methods involving activation of succinimide-based reagents required the use of Brønsted or Lewis acids often in combination with a Lewis base.^9,11−13^ Furthermore, the transfer of electron-deficient thioarenes was generally possible only using the most electron-rich arene substrates. This current method involving saccharin-based reagents overcomes these previous limitations. As well as allowing faster thioarylation of challenging substrates under milder conditions than with succinimide reagents, the current method is also effective for the efficient transfer of electron-deficient thioaryl groups with a range of activated arenes. Current work is underway to exploit the products of this process for further functionalization, as well as the development of other novel arene substitution reactions using the super Lewis acid iron(III) triflimide.

## Experimental Section

All reagents and starting materials were obtained from commercial sources and used as received.^20^ All reactions performed at elevated temperatures were heated by using an oil bath. Brine refers to a saturated aqueous solution of sodium chloride. Flash column chromatography was performed by using silica gel 60 (40–63 μm). Aluminum-backed plates precoated with silica gel 60F_254_ were used for thin-layer chromatography and were visualized with a UV lamp or by staining with potassium permanganate. ^1^H NMR spectra were recorded on a NMR spectrometer at either 400 or 500 MHz and data are reported as follows: chemical shift in ppm relative to the solvent as the internal standard (CHCl_3_,: δ 7.26 ppm; DMSO, δ 2.50), multiplicity (s = singlet, d = doublet, t = triplet, q = quartet, m = multiplet or overlap of nonequivalent resonances, integration). The abbreviation br s refers to a broad singlet. ^13^C NMR spectra were recorded on a NMR spectrometer at either 101 or 126 MHz, and data are reported as follows: chemical shift in ppm relative to tetramethylsilane or the solvent as the internal standard (CDCl_3_, δ 77.2 ppm; DMSO-*d*_6,_ δ 39.5), multiplicity with respect to hydrogen (deduced from DEPT experiments, C, CH, CH_2_, or CH_3_). Infrared spectra were recorded on an FTIR spectrometer; wavenumbers are indicated in cm^–1^. Mass spectra were recorded using electron impact (EI) or electrospray (ESI) techniques on a quadrupole time-of-flight (Q-TOF) mass spectrometer. Melting points are uncorrected. Optical rotations were determined as solutions irradiated with the sodium D line (λ = 589 nm) using a polarimeter. [α]_D_ values are given in units of 10^–1^ deg cm^–1^ g^–1^.

### General Procedure A: Preparation of *N*-(Thioaryl)saccharins

A solution of thiol (1.0 equiv) in dry dichloromethane (2 mL/mmol) was added to a stirred solution of *N*-chlorosaccharin (1.0 equiv) in dry dichloromethane (1 mL/mmol) at 0 °C under argon and stirred for 10 min. A solution of triethylamine (1.0 equiv) in dry dichloromethane (1 mL/mmol) was then added dropwise over a period of 10 min. The reaction mixture was stirred at 0 °C for 10 min. The reaction mixture was then diluted with dichloromethane (4 mL/mmol) and washed with water (4 mL/mmol). The aqueous layer was extracted with dichloromethane (2 × 4 mL/mmol), and the combined organic layers were washed with brine (8 mL/mmol). The organic phase was dried (MgSO_4_), filtered, and concentrated *in vacuo*. Purification by recrystallization gave the desired product.

### *N*-(4-Chlorophenylthio)saccharin (**5a**)

The reaction was performed according to general procedure A using *N*-chlorosaccharin (1.09 g, 5.00 mmol), 4-chlorothiophenol (0.723 g, 5.00 mmol), and triethylamine (0.699 mL, 5.00 mmol). Purification by recrystallization (toluene/hexane, 1.5:1) gave *N*-(4-chlorophenylthio)saccharin (**5a**) (1.22 g, 75%) as a white solid. Mp 193–194 °C; IR (neat) 3090, 1743, 1572, 1339, 1220, 1092, 943, 822, 747 cm^–1^; ^1^H NMR (400 MHz, CDCl_3_): δ 8.10 (br d, *J* = 7.5 Hz, 1H), 7.96 (br d, *J* = 7.5 Hz, 1H), 7.91 (td, *J* = 7.5, 1.2 Hz, 1H), 7.84 (td, *J* = 7.5, 1.4 Hz, 1H), 7.80–7.76 (m, 2H), 7.37–7.32 (m, 2H); ^13^C{^1^H} NMR (101 MHz, CDCl_3_): δ 159.5 (C), 138.2 (C), 137.4 (C), 135.7 (CH), 135.0 (2 × CH), 134.6 (CH), 131.5 (C), 129.7 (2 × CH), 127.1 (C), 126.1 (CH), 121.8 (CH); MS (ESI) *m*/*z* 325 (M + H^+^, 100); HRMS (ESI) *m*/*z*: [M + H]^+^ calcd for C_13_H_8_^35^ClNO_3_S_2_ 324.9629; found, 324.9629.

### *N*-(4-Nitrophenylthio)saccharin (**5b**)

The reaction was performed according to general procedure A using *N*-chlorosaccharin (0.435 g, 2.00 mmol), 4-nitrothiophenol (0.310 g, 2.00 mmol), and triethylamine (0.279 mL, 2.00 mmol). Purification by recrystallization (toluene/hexane, 1.5:1) gave *N*-(4-nitrophenylthio)saccharin (**5b**) (0.470 g, 70%) as a white solid. Mp 164–166 °C; IR (neat) 3106, 1749, 1514, 1342, 1189, 941, 839, 737 cm^–1^; ^1^H NMR (400 MHz, CDCl_3_): δ 8.23–8.16 (m, 3H), 8.03 (ddd, *J* = 7.5, 1.5, 0.7 Hz, 1H), 7.99 (td, *J* = 7.5, 1.2 Hz, 1H), 7.92 (td, *J* = 7.5, 1.5 Hz, 1H), 7.70–7.65 (m, 2H); ^13^C{^1^H} NMR (101 MHz, CDCl_3_): δ 159.3 (C), 147.9 (C), 142.6 (C), 138.3 (C), 136.2 (CH), 135.0 (CH), 128.1 (2 × CH), 126.7 (C), 126.4 (CH), 124.6 (2 × CH), 122.0 (CH); MS (ESI) *m*/*z* 359 (M + Na^+^, 100); HRMS (ESI) *m*/*z*: [M + Na]^+^ calcd for C_13_H_8_N_2_NaO_5_S_2_ 358.9767; found, 358.9779.

### *N*-(4-Methoxyphenylthio)saccharin (**5c**)

The reaction was performed according to general procedure A using *N*-chlorosaccharin (0.326 g, 1.50 mmol), 4-methoxythiophenol (0.184 mL, 1.50 mmol), and triethylamine (0.209 mL, 1.50 mmol). Purification by recrystallization (toluene/hexane, 1.5:1) gave *N*-(4-methoxyphenylthio)saccharin (**5c**) (0.346 g, 72%) as a white solid. Mp 166–169 °C; IR (neat) 2941, 1740, 1587, 1346, 1176, 948, 750 cm^–1^; ^1^H NMR (400 MHz, CDCl_3_): δ 8.06 (ddd, *J* = 7.5, 1.3, 0.7 Hz, 1H), 7.94–7.89 (m, 3H), 7.86 (td, *J* = 7.5, 1.3 Hz, 1H), 7.80 (td, *J* = 7.5, 1.3 Hz, 1H), 6.90–6.84 (m, 2H), 3.80 (s, 3H); ^13^C{^1^H} NMR (101 MHz, CDCl_3_): δ 162.4 (C), 159.5 (C), 138.3 (2 × CH and C), 135.4 (CH), 134.4 (CH), 127.4 (C), 125.9 (CH), 123.6 (C), 121.6 (CH), 114.8 (2 × CH), 55.6 (CH_3_); MS (ESI) *m*/*z* 321 (M^+^, 100); HRMS (ESI) *m*/*z*: [M]^+^ calcd for C_14_H_11_NO_4_S_2_ 321.0124; found, 321.0120.

### General Procedure B: Thioarylation of Arenes Using Iron(III) Triflimide (2.5 mol %)

Iron(III) chloride (0.0050 g, 0.0300 mmol, 2.5 mol %) was dissolved in 1-butyl-3-methylimidazolium bis(trifluoromethanesulfonyl)imide (0.026 mL, 0.0900 mmol, 7.5 mol %), stirred for 0.5 h at room temperature, and then added to a suspension of the *N*-(thioaryl)saccharin (0.330 mmol) in chloroform (0.6 mL). To the resultant mixture was added the arene (0.300 mmol), and the reaction mixture was stirred at room temperature for the required time. The reaction mixture was then diluted with dichloromethane (10 mL) and washed with water (10 mL). The aqueous layer was extracted with dichloromethane (2 × 10 mL), and the combined organic layers were washed with brine (20 mL). The organic phase was dried (MgSO_4_), filtered, and concentrated *in vacuo*. Purification by flash column chromatography gave the desired product.

### General Procedure C: Thioarylation of Arenes Using Iron(III) Triflimide (10 mol %)

Iron(III) chloride (0.020 g, 0.12 mmol, 10 mol %) was dissolved in 1-butyl-3-methylimidazolium bis(trifluoromethanesulfonyl)imide (0.10 mL, 0.36 mmol, 30 mol %), stirred for 0.5 h at room temperature, and then added to a suspension of the *N*-(thioaryl)saccharin (0.330 mmol) in chloroform (0.6 mL). To the resultant mixture was added the arene (0.300 mmol), and the reaction mixture was stirred at room temperature or 40 °C for the required time. The reaction mixture was then diluted with dichloromethane (10 mL) and washed with water (10 mL). The aqueous layer was extracted with dichloromethane (2 × 10 mL), and the combined organic layers were washed with brine (20 mL). The organic phase was dried (MgSO_4_), filtered, and concentrated *in vacuo*. Purification by flash column chromatography gave the desired product.

### (4′-Chlorophenyl)(4-methoxyphenyl)sulfane (**2a**)^21^

The reaction was performed according to general procedure B using anisole (33.0 μL, 0.300 mmol) and *N*-(4-chlorophenylthio)saccharin (**5a**) (0.108 g, 0.330 mmol). The reaction mixture was stirred at room temperature for 0.5 h. Purification by flash column chromatography (hexane/dichloromethane, 4:1) gave (4′-chlorophenyl)(4-methoxyphenyl)sulfane (**2a**) (0.0708 g, 94%) as a white solid. Mp 59–61 °C (lit.^21^ 59.2–60.5 °C); ^1^H NMR (400 MHz, CDCl_3_): δ 7.43–7.38 (m, 2H), 7.21–7.16 (m, 2H), 7.10–7.05 (m, 2H), 6.93–6.88 (m, 2H), 3.83 (s, 3H); ^13^C{^1^H} NMR (101 MHz, CDCl_3_): δ 160.2 (C), 137.5 (C), 135.6 (2 × CH), 131.8 (C), 129.5 (2 × CH), 129.2 (2 × CH), 124.0 (C), 115.3 (2 × CH), 55.5 (CH_3_); MS (EI) *m*/*z* 250 (M^+^, 100), 235 (39), 172 (24), 83 (22).

### (4′-Chlorophenyl)(4-methoxyphenyl)sulfane (**2a**),^21^ Large-Scale Reaction

The reaction was performed according to general procedure B using anisole (132 μL, 1.20 mmol) and *N*-(4-chlorophenylthio)saccharin (**5a**) (0.432 g, 1.32 mmol). The reaction mixture was stirred at room temperature for 0.5 h. Purification by flash column chromatography (hexane/dichloromethane, 4:1) gave (4′-chlorophenyl)(4-methoxyphenyl)sulfane (**2a**) (0.271 g, 90%) as a white solid. Spectroscopic data are as described above.

### (4-Methoxyphenyl)(4′-nitrophenyl)sulfane (**2b**)^22^

The reaction was performed according to general procedure C using anisole (0.033 mL, 0.300 mmol), *N*-(4-nitrophenylthio)saccharin (**5b**) (0.121 g, 0.360 mmol), iron(III) chloride (0.0049 g, 0.0300 mmol, 10 mol %), and 1-butyl-3-methylimidazolium bis(trifluoromethanesulfonyl)imide (0.026 mL, 0.0900 mmol, 30 mol %). The reaction mixture was stirred at room temperature for 5 h. Purification by flash column chromatography (40% dichloromethane in hexane) gave (4-methoxyphenyl)(4′-nitrophenyl)sulfane (**2b**) (0.0748 g, 95%) as a yellow solid. Mp 63–65 °C (lit.^22^ 63–65 °C); ^1^H NMR (400 MHz, CDCl_3_): δ 8.07–8.01 (m, 2H), 7.52–7.44 (m, 2H), 7.12–7.05 (m, 2H), 7.02–6.95 (m, 2H), 3.87 (s, 3H); ^13^C{^1^H} NMR (101 MHz, CDCl_3_): δ 161.3 (C), 150.2 (C), 145.2 (C), 137.3 (2 × CH), 125.7 (2 × CH), 124.1 (2 × CH), 120.3 (C), 115.8 (2 × CH), 55.6 (CH_3_); MS (EI) *m*/*z* 284 (M + Na^+^, 100).

### (4-Methoxynaphthalen-1-yl)(4′-methoxyphenyl)sulfane (2c)^23^

The reaction was performed according to general procedure B using 1-methoxynaphthalene (43.5 μL, 0.300 mmol) and *N*-(4-methoxyphenylthio)saccharin (**5c**) (0.106 g, 0.330 mmol). The reaction mixture was stirred at room temperature for 0.5 h. Purification by flash column chromatography (hexane/dichloromethane, 4:1) gave (4-methoxynaphthalen-1-yl)(4′-methoxyphenyl)sulfane (**2c**) (0.0844 g, 95%) as a white solid. Mp 87–88 °C (lit.^23^ 83–85 °C); ^1^H NMR (500 MHz, CDCl_3_): δ 8.36 (dd, *J* = 7.9, 1.0 Hz, 1H), 8.30 (dd, *J* = 7.9, 1.4 Hz, 1H), 7.66 (d, *J* = 8.0 Hz, 1H), 7.56–7.48 (m, 2H), 7.16–7.10 (m, 2H), 6.79 (d, *J* = 8.0 Hz, 1H), 6.78–6.74 (m, 2H), 4.02 (s, 3H), 3.74 (s, 3H); ^13^C{^1^H} NMR (126 MHz, CDCl_3_): δ 158.4 (C), 156.6 (C), 134.7 (C), 133.9 (CH), 130.4 (2 × CH), 128.7 (C), 127.5 (CH), 126.6 (C), 125.9 (CH), 125.8 (CH), 122.7 (C), 122.6 (CH), 114.8 (2 × CH), 104.1 (CH), 55.8 (CH_3_), 55.5 (CH_3_); MS (ESI) *m*/*z* 319 (M + Na^+^, 100).

### [1-(Phenylsulfonyl)indol-3-yl](4′-methoxyphenyl)sulfane (**2d**)^24^

The reaction was performed according to general procedure B using 1-(phenylsulfonyl)indole (0.0772 g, 0.300 mmol) and *N*-(4-methoxyphenylthio)saccharin (**5c**) (0.106 g, 0.330 mmol). The reaction mixture was stirred at room temperature for 0.5 h. Purification by flash column chromatography (50% dichloromethane in hexane) gave [1-(phenylsulfonyl)indol-3-yl](4′-methoxyphenyl)sulfane (**2d**) (0.110 g, 93%) as a white solid. Mp 85–88 °C (lit.^24^ 86–88 °C); ^1^H NMR (400 MHz, CDCl_3_): δ 8.00 (d, *J* = 8.3 Hz, 1H), 7.92–7.87 (m, 2H), 7.70 (s, 1H), 7.59–7.53 (m, 1H), 7.48–7.44 (m, 3H), 7.36–7.32 (m, 1H), 7.23–7.18 (m, 3H), 6.79–6.75 (m, 2H), 3.76 (s, 3H); ^13^C{^1^H} NMR (101 MHz, CDCl_3_): δ 158.9 (C), 138.1 (C), 135.6 (C), 134.2 (CH and C), 131.0 (2 × CH), 129.5 (2 × CH and C), 129.2 (CH), 127.0 (2 × CH), 125.8 (C), 125.5 (CH), 123.9 (CH), 120.5 (CH), 114.9 (2 × CH), 113.9 (CH), 55.5 (CH_3_); MS (ESI) *m*/*z* 418 (M + Na^+^, 100).

### *N*-(Benzyloxycarbonyl)-[3′-(4″-methoxyphenylthio)]-l-tyrosine Methyl Ester (**2e**)

The reaction was performed as described in general procedure B using *N*-(benzyloxycarbonyl)-l-tyrosine methyl ester (0.100 g, 0.300 mmol) and *N*-(4-methoxyphenylthio)saccharin (**5c**) (0.106 g, 0.330 mmol). The reaction mixture was stirred at 50 °C for 0.25 h. Purification by flash column chromatography (dichloromethane/diethyl ether, 100:2) gave *N*-(benzyloxycarbonyl)-[3′-(4″-methoxyphenylthio)]-l-tyrosine methyl ester (**2e**) (0.0836 g, 53%) as a colorless oil. [α]_D_^25^ +40.7 (c 0.1, CHCl_3_); IR (neat) 3408, 2949, 1713, 1591, 1491, 1244, 1026, 826, 754 cm^–1^; ^1^H NMR (400 MHz, CDCl_3_): δ 7.40–7.27 (m, 5H), 7.23 (d, *J* = 2.0 Hz, 1H), 7.15–7.07 (m, 2H), 7.03 (dd, *J* = 8.3, 2.0 Hz, 1H), 6.92 (d, *J* = 8.3 Hz, 1H), 6.78 (d, *J* = 8.8 Hz, 2H), 6.53 (s, 1H), 5.27 (d, *J* = 7.9 Hz, 1H), 5.09 (s, 2H), 4.65–4.58 (m, 1H), 3.73 (s, 3H), 3.64 (s, 3H), 3.06 (dd, *J* = 14.0, 5.6 Hz, 1H), 2.99 (dd, *J* = 14.0, 5.8 Hz, 1H); ^13^C{^1^H} NMR (101 MHz, CDCl_3_): δ 171.9 (C), 159.0 (C), 155.9 (C), 155.7 (C), 136.7 (CH), 136.3 (C), 132.6 (CH), 130.3 (2 × CH), 128.7 (2 × CH), 128.5 (C), 128.3 (CH), 128.2 (2 × CH), 126.0 (C), 118.9 (C), 115.7 (CH), 115.1 (2 × CH), 67.1 (CH_2_), 55.5 (CH_3_), 55.0 (CH), 52.4 (CH_3_), 37.4 (CH_2_); MS (ESI) *m*/*z* 490 (M + Na^+^, 100); HRMS (ESI) *m*/*z*: [M + Na]^+^ calcd for C_25_H_25_NNaO_6_S 490.1295; found, 490.1293.

### *N*-Acetyl-[2′-(4″-methoxyphenylthio)]-l-tryptophan Methyl Ester (**2f**)

The reaction was performed according to general procedure B using methyl *N*-acetyl-l-tryptophanate (0.0781 g, 0.300 mmol) and *N*-(4-methoxyphenylthio)saccharin (**5c**) (0.106 g, 0.330 mmol). The reaction mixture was stirred at room temperature for 1.5 h. Purification by flash column chromatography (50–70% ethyl acetate in hexane) gave *N*-acetyl-[2′-(4″-methoxyphenylthio)]-l-tryptophan methyl ester (**2f**) (0.108 g, 91%) as a white solid. Mp 139–142 °C; [α]_D_^22^ +39.1 (*c* 0.1, CHCl_3_); IR (neat) 3368, 3281, 2949, 1736, 1655, 1491, 1242, 1173, 1028, 824, 743 cm^–1^; ^1^H NMR (400 MHz, CDCl_3_): δ 8.11 (s, 1H), 7.55 (d, *J* = 7.9 Hz, 1H), 7.26 (d, *J* = 8.0 Hz, 1H), 7.22–7.17 (m, 1H), 7.16–7.09 (m, 3H), 6.83–6.78 (m, 2H), 6.04 (d, *J* = 7.7 Hz, 1H), 4.92 (dt, *J* = 7.7, 5.9 Hz, 1H), 3.76 (s, 3H), 3.70 (s, 3H), 3.44 (dd, *J* = 14.5, 5.9 Hz, 1H), 3.37 (dd, *J* = 14.5, 5.9 Hz, 1H), 1.86 (s, 3H); ^13^C{^1^H} NMR (101 MHz, CDCl_3_): δ 172.4 (C), 169.9 (C), 159.1 (C), 136.9 (C), 130.5 (2 × CH), 128.2 (C), 125.9 (C), 125.8 (C), 123.5 (CH), 120.3 (CH), 119.1 (CH), 115.8 (C), 115.2 (2 × CH), 111.1 (CH), 55.6 (CH_3_), 53.0 (CH), 52.7 (CH_3_), 27.5 (CH_2_), 23.3 (CH_3_); MS (ESI) *m*/*z* 421 (M + Na^+^, 100); HRMS (ESI) *m*/*z*: [M + Na]^+^ calcd for C_21_H_22_N_2_NaO_4_S 421.1192; found, 421.1194.

### 5-{[4′-(4-Methoxyphenylthio)-3′,5′-dimethylphenoxy]methyl}oxazolidin-2″-one (**2g**)^8d^

The reaction was performed according to general procedure B using metaxalone (0.0664 g, 0.300 mmol) and *N*-(4-methoxyphenylthio)saccharin (**5c**) (0.106 g, 0.330 mmol). The reaction mixture was stirred at room temperature for 2 h. Purification by flash column chromatography (70–100% ethyl acetate in hexane) gave 5-{[4′-(4-methoxyphenylthio)-3′,5′-dimethylphenoxy]methyl}oxazolidin-2″-one (**2g**) (0.0969 g, 90%) as a white solid. Mp 38–41 °C (lit.^8d^ 40.3–42.5 °C); ^1^H NMR (400 MHz, CDCl_3_): δ 6.89–6.83 (m, 2H), 6.78–6.69 (m, 4H), 6.26 (s, 1H), 5.00–4.92 (m, 1H), 4.12 (d, *J* = 4.9 Hz, 2H), 3.78–3.71 (m, 4H), 3.61–3.54 (m, 1H), 2.39 (s, 6H); ^13^C{^1^H} NMR (101 MHz, CDCl_3_): δ 160.3 (C), 158.3 (C), 157.5 (C), 145.6 (2 × C), 129.0 (C), 127.5 (2 × CH), 124.1 (C), 114.8 (2 × CH), 114.5 (2 × CH), 74.6 (CH), 67.9 (CH_2_), 55.4 (CH_3_), 42.8 (CH_2_), 22.3 (2 × CH_3_); MS (ESI) *m*/*z* 382 (M + Na^+^, 100).

### (4′-Chlorophenyl)(1-methoxy-2-methylphenyl)sulfane (**6a**)

The reaction was performed according to general procedure B using *N*-(4-chlorophenylthio)saccharin (**5a**) (0.108 g, 0.330 mmol) and 2-methylmethoxybenzene (37.2 μL, 0.300 mmol). The reaction mixture was stirred at room temperature for 3 h. Purification by flash column chromatography (20% dichloromethane in hexane) gave (0.0818 g, 91%) as a white solid. Mp 80–82 °C; IR (neat) 3062, 2956, 1591, 1493, 1474, 1464, 1454, 1252 cm^–1^; ^1^H NMR (400 MHz, CDCl_3_): δ 7.30 (d, *J* = 8.2 Hz, 1H), 7.26 (s, 1H), 7.19 (d, *J* = 8.0 Hz, 2H), 7.07 (d, *J* = 8.0 Hz, 2H), 6.82 (d, *J* = 8.2 Hz, 1H), 3.85 (s, 3H), 2.20 (s, 3H); ^13^C{^1^H} NMR (101 MHz, CDCl_3_): δ 158.5 (C), 137.9 (C), 136.5 (CH), 133.2 (CH), 131.5 (C), 129.2 (2 × CH), 129.1 (2 × CH), 128.4 (C), 123.0 (C), 110.9 (CH), 55.6 (CH_3_), 16.3 (CH_3_); MS (ESI) *m*/*z* 264 (M^+^, 100); HRMS (ESI) *m*/*z*: [M]^+^ calcd for C_14_H_13_^35^ClOS 264.0376; found, 264.0372.

### (4′-Chlorophenyl)(1-methoxynaphthalen-4-yl)sulfane (**6b**)

The reaction was performed according to general procedure B using *N*-(4-chlorophenylthio)saccharin (**5a**) (0.108 g, 0.330 mmol) and 1-methoxynaphthalene (43.5 μL, 0.300 mmol). The reaction mixture was stirred at room temperature for 4 h. Purification by flash column chromatography (5% diethyl ether in hexane) gave (4′-chlorophenyl)(1-methoxynaphthalen-4-yl)sulfane (**6b**) (0.0836 g, 93%) as a white solid. Mp 87–90 °C; IR (neat) 3043, 2912, 1568, 1490, 1444, 1250 cm^–1^; ^1^H NMR (400 MHz, CDCl_3_): δ 8.36–8.25 (m, 2H), 7.80 (d, *J* = 8.1 Hz, 1H), 7.56–7.49 (m, 2H), 7.11 (dt, *J* = 8.6, 2.8 Hz, 2H), 6.95 (dt, *J* = 8.6, 2.8 Hz, 2H), 6.84 (d, *J* = 8.1 Hz, 1H), 4.05 (s, 3H); ^13^C{^1^H} NMR (101 MHz, CDCl_3_): δ 157.5 (C), 137.8 (C), 136.2 (CH), 135.2 (C), 131.0 (C), 129.0 (2 × CH), 128.0 (CH), 127.9 (2 × CH), 126.8 (C), 126.0 (CH), 125.9 (CH), 122.8 (CH), 119.6 (C), 104.2 (CH), 55.8 (CH_3_); MS (ESI) *m*/*z* 301 (M + H^+^, 100); HRMS (ESI) *m*/*z*: [M + H]^+^ calcd for C_17_H_14_^35^ClOS 301.0454; found, 301.0450.

### (4-Hydroxyphenyl)(4′-chlorophenyl)sulfane (**6c**)^25^

The reaction was performed according to general procedure B using phenol (0.0282 g, 0.300 mmol) and *N*-(4-chlorophenylthio)saccharin (**5a**) (0.108 g, 0.330 mmol). The reaction mixture was stirred at room temperature for 0.1 h. Purification by flash column chromatography (10–20% ethyl acetate in hexane) gave (4-hydroxyphenyl)(4′-chlorophenyl)sulfane (**6c**) (0.0526 g, 74%) as a white solid. Mp 64–66 °C (lit.^25^ 65–66 °C); ^1^H NMR (400 MHz, CDCl_3_): δ 7.39–7.33 (m, 2H), 7.23–7.17 (m, 2H), 7.12–7.06 (m, 2H), 6.87–6.81 (m, 2H), 5.02 (s, 1H); ^13^C{^1^H} NMR (101 MHz, CDCl_3_): δ 156.2 (C), 137.3 (C), 135.8 (2 × CH), 131.9 (C), 129.6 (2 × CH), 129.2 (2 × CH), 124.3 (C), 116.8 (2 × CH); MS (ESI) *m*/*z* 236 (M^+^, 100).

### (2-Bromo-4-hydroxyphenyl)(4′-chlorophenyl)sulfane (**6d**)

The reaction was performed according to general procedure B using 3-bromophenol (0.0320 mL, 0.300 mmol) and *N*-(4-chlorophenylthio)saccharin (**5a**) (0.108 g, 0.330 mmol). The reaction mixture was stirred at room temperature for 1 h. Purification by flash column chromatography (5–10% ethyl acetate in hexane) gave (2-bromo-4-hydroxyphenyl)(4′-chlorophenyl)sulfane (**6d**) (0.0586 g, 62%) as a white solid. Mp 103–104 °C; IR (neat) 3346, 2918, 1590, 1572, 1465, 1212, 1089, 1010 cm^–1^; ^1^H NMR (400 MHz, CDCl_3_): δ 7.28 (d, *J* = 8.5 Hz, 1H), 7.28–7.22 (m, 2H), 7.18 (d, *J* = 2.7 Hz, 1H), 7.16–7.10 (m, 2H), 6.77 (dd, *J* = 8.5, 2.7 Hz, 1H), 5.26 (s, 1H); ^13^C{^1^H} NMR (101 MHz, CDCl_3_): δ 156.5 (C), 136.0 (CH), 135.0 (C), 132.7 (C), 130.7 (2 × CH), 129.5 (2 × CH), 128.8 (C), 126.3 (C), 120.9 (CH), 115.9 (CH); MS (ESI) *m*/*z* 316 (M^+^, 100); HRMS (ESI) *m*/*z*: [M]^+^ calcd for C_12_H_8_^81^Br^35^ClOS 315.9140; found, 315.9140.

### (2,6-Dimethyl-4-hydroxyphenyl)(4′-chlorophenyl)sulfane (**6e**)

The reaction was performed according to general procedure B using 3,5-dimethylphenol (0.0369 g, 0.300 mmol) and *N*-(4-chlorophenylthio)saccharin (**5a**) (0.0977 g, 0.300 mmol). The reaction mixture was stirred at room temperature for 1 h. Purification by flash column chromatography (10% ethyl acetate in hexane) gave (2,6-dimethyl-4-hydroxyphenyl)(4′-chlorophenyl)sulfane (**6e**) (0.0504 g, 63%) as a white solid. Mp 103–104 °C; IR (neat) 3261, 2921, 1584, 1473, 1299, 1160, 1090, 1008 cm^–1^; ^1^H NMR (400 MHz, CDCl_3_): δ 7.17–7.11 (m, 2H), 6.86–6.79 (m, 2H), 6.69 (s, 2H), 4.98 (br s, 1H), 2.36 (s, 6H); ^13^C{^1^H} NMR (101 MHz, CDCl_3_): δ 156.5 (C), 146.1 (2 × C), 137.4 (C), 130.3 (C), 129.1 (2 × CH), 126.5 (2 × CH), 121.3 (C), 115.6 (2 × CH), 22.0 (2 × CH_3_); MS (ESI) *m*/*z* 264 (M^+^, 100); HRMS (ESI) *m*/*z*: [M]^+^ calcd for C_14_H_13_^35^ClOS 264.0370; found, 264.0373.

### Benzyl [4-(4′-chlorophenylthio)phenyl]carbamate (**6f**)

The reaction was performed according to general procedure B using *N*-(benzyloxycarbonyl)aniline (0.0681 g, 0.300 mmol) and *N*-(4-chlorophenylthio)saccharin (**5a**) (0.108 g, 0.330 mmol). The reaction mixture was stirred at room temperature for 2 h. Purification by flash column chromatography (20% diethyl ether in hexane) gave benzyl [4-(4′-chlorophenylthio)phenyl]carbamate (**6f**) (0.0968 g, 87%) as a white solid. Mp 125–126 °C; IR (neat) 3282, 3066, 1696, 1591, 1519, 1250, 1065, 812 cm^–1^; ^1^H NMR (400 MHz, CDCl_3_): δ 7.43–7.32 (m, 9H), 7.25–7.19 (m, 2H), 7.17–7.11 (m, 2H), 6.72 (br s, 1H), 5.21 (s, 2H); ^13^C{^1^H} NMR (101 MHz, CDCl_3_): δ 153.2 (C), 138.1 (C), 136.2 (C), 136.0 (C), 134.0 (2 × CH), 132.4 (C), 130.6 (2 × CH), 129.3 (2 × CH), 128.8 (2 × CH), 128.6 (CH), 128.5 (2 × CH), 128.2 (C), 119.6 (2 × CH), 67.4 (CH_2_); MS (ESI) *m*/*z* 370 (M + H^+^, 100); HRMS (ESI) *m*/*z*: [M + H]^+^ calcd for C_20_H_17_^35^ClNO_2_S 370.0663; found, 370.0667.

### Mesityl-(4′-chlorophenyl)sulfane (**6g**)^26^

The reaction was performed according to general procedure B using mesitylene (0.0420 mL, 0.300 mmol) and *N*-(4-chlorophenylthio)saccharin (**5a**) (0.108 g, 0.330 mmol). The reaction mixture was stirred at room temperature for 6 h. Purification by flash column chromatography (hexane) gave mesityl-(4′-chlorophenyl)sulfane (**6g**) (0.0754 g, 85%) as a white solid. Mp 73–75 °C (lit.^26^ 78–80 °C); ^1^H NMR (400 MHz, CDCl_3_): δ 7.17–7.11 (m, 2H), 7.02 (s, 2H), 6.87–6.81 (m, 2H), 2.38 (s, 6H), 2.33 (s, 3H); ^13^C{^1^H} NMR (101 MHz, CDCl_3_): δ 143.8 (2 × C), 139.7 (C), 137.2 (C), 130.4 (C), 129.6 (2 × CH), 129.1 (2 × CH), 126.8 (2 × CH), 126.7 (C), 21.8 (2 × CH_3_), 21.3 (CH_3_); MS (ESI) *m*/*z* 262 (M^+^, 100).

### 3-(4′-Chlorophenylthio)indole (**6h**)^27^

The reaction was performed according to general procedure B using indole (0.0351 g, 0.300 mmol) and *N*-(4-chlorophenylthio)saccharin (**5a**) (0.0977 g, 0.300 mmol). The reaction mixture was stirred at room temperature for 0.25 h. Purification by flash column chromatography (10% ethyl acetate in hexane) gave 3-(4′-chlorophenylthio)indole (**6h**) (0.0743 g, 95%) as a white solid. Mp 132–134 °C (lit.^27^ 134–135 °C); ^1^H NMR (400 MHz, CDCl_3_): δ 8.40 (br s, 1H), 7.58 (dd, *J* = 8.0, 1.2 Hz, 1H), 7.49 (d, *J* = 2.6 Hz, 1H), 7.45 (dt, *J* = 8.2, 1.0 Hz, 1H), 7.29 (ddd, *J* = 8.2, 7.1, 1.2 Hz 1H), 7.19 (ddd, *J* = 8.0, 7.1, 1.0 Hz, 1H), 7.15–7.10 (m, 2H), 7.06–7.00 (m, 2H); ^13^C{^1^H} NMR (101 MHz, CDCl_3_): δ 138.0 (C), 136.7 (C), 130.8 (CH), 130.7 (C), 128.94 (C), 128.89 (2 × CH), 127.3 (2 × CH), 123.4 (CH), 121.2 (CH), 119.7 (CH), 111.8 (CH), 102.6 (C); MS (ESI) *m*/*z* 260 (M + H^+^, 100).

### 3-(4′-Chlorophenylthio)-5-nitroindole (**6i**)^27^

The reaction was performed according to general procedure B using 5-nitroindole (0.0486 g, 0.300 mmol) and *N*-(4-chlorophenylthio)saccharin (**5a**) (0.108 g, 0.330 mmol). The reaction mixture was stirred at room temperature for 18 h. Purification by flash column chromatography (40% ethyl acetate in hexane) gave 3-(4′-chlorophenylthio)-5-nitroindole (**6i**) (0.0869 g, 95%) as a yellow solid. Mp 213–215 °C (lit.^27^ 215–217 °C); ^1^H NMR (400 MHz, CDCl_3_): δ 8.84 (br s, 1H), 8.54 (br s, 1H), 8.19 (dd, *J* = 8.5, 2.3 Hz, 1H), 7.67 (d, *J* = 2.3 Hz, 1H), 7.52 (d, *J* = 8.5 Hz, 1H), 7.16 (d, *J* = 8.6 Hz, 2H), 7.04 (d, *J* = 8.6 Hz, 2H); ^13^C{^1^H} NMR (101 MHz, CDCl_3_): δ 143.2 (C), 139.6 (C), 136.5 (C), 133.8 (CH), 131.6 (C), 129.2 (2 × CH), 128.8 (C), 127.8 (2 × CH), 119.1 (CH), 116.9 (CH), 112.1 (CH), 106.4 (C); MS (ESI) *m*/*z* 304 (M^+^, 100).

### *N*-Acetyl-[2′-(4″-chlorophenylthio)]-l-tryptophan Methyl Ester (**6j**)

The reaction was performed according to general procedure B using methyl *N*-acetyl-l-tryptophanate (0.0781 g, 0.300 mmol) and *N*-(4-chlorophenylthio)saccharin (**5a**) (0.108 g, 0.330 mmol). The reaction mixture was stirred at room temperature for 1 h. Purification by flash column chromatography (60–80% ethyl acetate in hexane) gave *N*-acetyl-[2′-(4″-methoxyphenylthio)]-l-tryptophan methyl ester (**6j**) (0.114 g, 95%) as a white solid. Mp 78–79 °C; [α]_D_^19^ +60.7 (*c* 0.1, CHCl_3_); IR (neat) 3250, 2951, 1733, 1653, 1475, 1216, 1089, 1009, 814 cm^–1^; ^1^H NMR (400 MHz, CDCl_3_): δ 8.24 (br s, 1H), 7.60 (br d, *J* = 8.0 Hz, 1H), 7.31 (br d, *J* = 8.2 Hz, 1H), 7.28–7.13 (m, 4H), 7.01–6.95 (m, 2H), 6.00 (d, *J* = 7.8 Hz, 1H), 4.91 (dt, *J* = 7.8, 5.9 Hz, 1H), 3.68 (s, 3H), 3.42 (dd, *J* = 14.5, 5.9 Hz, 1H), 3.33 (dd, *J* = 14.5, 5.9 Hz, 1H), 1.85 (s, 3H); ^13^C{^1^H} NMR (101 MHz, CDCl_3_): δ 172.3 (C), 169.9 (C), 137.2 (C), 135.0 (C), 132.4 (C), 129.6 (2 × CH), 128.3 (2 × CH), 128.1 (C), 124.1 (CH), 123.0 (C), 120.5 (CH), 119.4 (CH), 118.0 (C), 111.3 (CH), 53.0 (CH), 52.7 (CH_3_), 27.6 (CH_2_), 23.3 (CH_3_); MS (ESI) *m*/*z* 401 ([M – H]^−^, 100); HRMS (ESI) *m*/*z*: [M – H]^−^ calcd for C_20_H_18_^35^ClN_2_O_3_S 401.0732; found, 401.0732.

### (4′-Nitrophenyl)(3-methyl-4-methoxyphenyl)sulfane (**7a**)

The reaction was performed as described in general procedure C using 2-methylanisole (28.5 μL, 0.230 mmol) and *N*-(4-nitrophenylthio)saccharin (**5b**) (0.100 g, 0.300 mmol). The reaction mixture was stirred at room temperature for 23 h. Purification by flash column chromatography (0–40% dichloromethane in hexane) gave (4′-nitrophenyl)(3-methyl-4-methoxyphenyl)sulfane (**7a**) (0.0540 g, 85%) as a yellow solid. Mp 123–125 °C; IR (neat) 2917, 1575, 1503, 1334, 1248, 1082, 1029 cm^–1^; ^1^H NMR (400 MHz, CDCl_3_): δ 8.05–7.98 (m, 2H), 7.38 (dd, *J* = 8.4, 2.4 Hz, 1H), 7.31 (d, *J* = 2.4 Hz, 1H), 7.12–7.05 (m, 2H), 6.90 (d, *J* = 8.4 Hz, 1H), 3.89 (s, 3H), 2.23 (s, 3H); ^13^C{^1^H} NMR (101 MHz, CDCl_3_): δ 159.4 (C), 150.5 (C), 145.0 (C), 137.7 (CH), 134.8 (CH), 129.0 (C), 125.6 (2 × CH), 124.0 (2 × CH), 119.4 (C), 111.3 (CH), 55.6 (CH_3_), 16.3 (CH_3_); MS (ESI) *m*/*z* 275 (M^+^, 100); HRMS (ESI) *m*/*z*: [M]^+^ calcd for C_14_H_13_NO_3_S 275.0611; found, 275.0610.

### (4-Methoxynaphthalen-1-yl)(4′-nitrophenyl)sulfane (**7b**)^28^

The reaction was performed as described in general procedure C using 1-methoxynaphthalene (33.0 μL, 0.230 mmol) and *N*-(4-nitrophenylthio)saccharin (**5b**) (0.100 g, 0.300 mmol). The reaction mixture was stirred at 40 °C for 4.5 h. Purification by flash column chromatography (50% dichloromethane in hexane) gave (4-methoxynaphthalen-1-yl)(4′-nitrophenyl)sulfane (**7b**) (0.0430 g, 61%) as a yellow solid. Spectroscopic data were consistent with the literature.^28^ Mp 161–166 °C; ^1^H NMR (400 MHz, CDCl_3_): δ 8.40–8.34 (m, 1H), 8.19–8.14 (m, 1H), 7.99–7.94 (m, 2H), 7.85 (d, *J* = 8.0 Hz, 1H), 7.58–7.51 (m, 2H), 7.03–6.97 (m, 2H), 6.88 (d, *J* = 8.0 Hz, 1H), 4.08 (s, 3H); ^13^C{^1^H} NMR (101 MHz, CDCl_3_): δ 158.2 (C), 149.8 (C), 145.0 (C), 137.0 (CH), 135.1 (C), 128.4 (CH), 126.9 (C), 126.2 (CH), 125.4 (3 × CH), 124.0 (2 × CH), 123.1 (CH), 117.0 (C), 104.3 (CH), 55.9 (CH_3_); MS (ESI) *m*/*z* 312 (M + H^+^, 100).

### (4′-Nitrophenyl)(2,4,6-trimethoxyphenyl)sulfane (**7c**)^29^

The reaction was performed as described in general procedure C using 1,3,5-trimethoxybenzene (0.0385 g, 0.230 mmol) and *N*-(4-nitrophenylthio)saccharin (**5b**) (0.100 g, 0.300 mmol). The reaction mixture was stirred at room temperature for 5 h. Purification by flash column chromatography (80% dichloromethane in hexane) gave (4′-nitrophenyl)(2,4,6-trimethoxyphenyl)sulfane (**7c**) (0.0454 g, 62%) as a light-yellow solid. Mp 131–135 °C (lit.^29^ 132–135 °C); ^1^H NMR (400 MHz, CDCl_3_): δ 8.03–7.98 (m, 2H), 7.08–7.02 (m, 2H), 6.24 (s, 2H), 3.90 (s, 3H), 3.81 (s, 6H); ^13^C{^1^H} NMR (101 MHz, CDCl_3_): δ 163.9 (C), 162.6 (2 × C), 149.6 (C), 144.8 (C), 125.0 (2 × CH), 123.9 (2 × CH), 96.1 (C), 91.4 (2 × CH), 56.5 (2 × CH_3_), 55.7 (CH_3_); MS (ESI) *m*/*z* 322 (M + H^+^, 100).

### (2,3-Dihydro-1-benzofuran-5-yl)(4′-nitrophenyl)sulfane (**7d**)

The reaction was performed as described in general procedure C using 2,3-dihydrobenzofuran (26 μL, 0.23 mmol) and *N*-(4-nitrophenylthio)saccharin (**5b**) (0.10 g, 0.30 mmol). The reaction mixture was stirred at 40 °C for 4.5 h. Purification by flash column chromatography (0-50% dichloromethane in hexane) gave (2,3-dihydro-1-benzofuran-5-yl)(4′-nitrophenyl)sulfane (**7d**) (0.044 g, 70%) as a yellow solid. Mp 98–100 °C; IR (neat) 3097, 2897, 1575, 1506, 1332, 1230, 1084 cm^–1^; ^1^H NMR (400 MHz, CDCl_3_): δ 8.06–7.99 (m, 2H), 7.36 (br s, 1H), 7.32 (d, *J* = 8.2 Hz, 1H), 7.12–7.06 (m, 2H), 6.85 (d, *J* = 8.2 Hz, 1H), 4.66 (t, *J* = 8.8 Hz, 2H), 3.26 (t, *J* = 8.8 Hz, 2H); ^13^C{^1^H} NMR (101 MHz, CDCl_3_): δ 162.1 (C), 150.6 (C), 145.0 (C), 136.4 (CH), 132.5 (CH), 129.7 (C), 125.6 (2 × CH), 124.0 (2 × CH), 119.6 (C), 111.0 (CH), 72.0 (CH_2_), 29.5 (CH_2_); MS (ESI) *m*/*z* 273 (M^+^, 100); HRMS (ESI) *m*/*z*: [M]^+^ calcd for C_14_H_11_NO_3_S 273.0454; found, 273.0446.

### (2-Bromo-4-hydroxyphenyl)(4′-nitrophenyl)sulfane (**7e**)

The reaction was performed as described in general procedure C using 3-bromophenol (24.0 μL, 0.230 mmol) and *N*-(4-nitrophenylthio)saccharin (**5b**) (0.116 g, 0.350 mmol). The reaction mixture was stirred at 40 °C for 48 h. Purification by flash chromatography (100% dichloromethane) gave (2-bromo-4-hydroxyphenyl)(4′-nitrophenyl)sulfane (**7e**) (0.0543 g, 74%) as a yellow solid. Mp 161–165 °C; IR (neat) 3423, 1568, 1497, 1331, 1283, 1086 cm^–1^; ^1^H NMR (400 MHz, DMSO-*d*_6_): δ 10.61 (br s, 1H), 8.16–8.07 (m, 2H), 7.60 (d, *J* = 8.4 Hz, 1H), 7.26 (d, *J* = 2.4 Hz, 1H), 7.18–7.10 (m, 2H), 6.93 (dd, *J* = 8.4, 2.4 Hz, 1H); ^13^C{^1^H} NMR (101 MHz, DMSO-*d*_6_): δ 160.5 (C), 147.9 (C), 144.8 (C), 139.2 (CH), 131.1 (C), 125.4 (2 × CH), 124.3 (2 × CH), 120.9 (CH), 118.1 (C), 116.8 (CH); MS (ESI) *m*/*z* 350 (M + Na^+^, 100); HRMS (ESI) *m*/*z*: [M + Na]^+^ calcd for C_12_H_8_^81^BrNNaO_3_S 349.9280; found, 349.9274.

### Benzyl [4-(4′-Nitrophenylthio)phenyl]carbamate (**7f**)

The reaction was performed as described in general procedure C using *N*-(benzyloxycarbonyl)aniline (0.0520 g, 0.230 mmol) and *N*-(4-nitrophenylthio)saccharin (**5b**) (0.100 g, 0.300 mmol). The reaction mixture was stirred at 40 °C for 24 h. Purification by flash column chromatography (10–20% ethyl acetate in hexane) gave benzyl [4-(4′-nitrophenylthio)phenyl] carbamate (**7f**) (0.0527 g, 61%) as a light-yellow solid. Mp 132–135 °C; IR (neat) 3281, 1697, 1512, 1335, 1229, 1063, 826 cm^–1^; ^1^H NMR (400 MHz, CDCl_3_): δ 8.06–8.00 (m, 2H), 7.54–7.46 (m, 4H), 7.43–7.32 (m, 5H), 7.14–7.08 (m, 2H), 7.02 (br s, 1H), 5.22 (s, 2H); ^13^C{^1^H} NMR (101 MHz, CDCl_3_): δ 153.2 (C), 149.4 (C), 145.2 (C), 139.7 (C), 136.4 (2 × CH), 135.8 (C), 128.8 (2 × CH), 128.6 (2 × CH), 128.4 (2 × CH), 126.1 (2 × CH), 124.1 (2 × CH), 123.6 (C), 119.9 (CH), 67.4 (CH_2_); MS (ESI) *m*/*z* 379 ([M – H]^−^, 100); HRMS (ESI) *m*/*z*: [M – H]^−^ calcd for C_20_H_16_N_2_O_4_S 379.0758; found, 379.0755.

### Mesityl-(4′-nitrophenyl)sulfane (**7g**)^26^

The reaction was performed as described in general procedure C using mesitylene (31.8 μL, 0.230 mmol) and *N*-(4-nitrophenylthio)saccharin (**5b**) (0.100 g, 0.300 mmol). The reaction mixture was stirred at 40 °C for 29 h. Purification by flash column chromatography (20% dichloromethane in hexane) gave mesityl-(4′-nitrophenyl)sulfane (**7g**) (0.0398 g, 63%) as a light-yellow solid. Mp 83–86 °C (lit.^26^ 86–88 °C); ^1^H NMR (400 MHz, CDCl_3_): δ 8.03 (d, *J* = 9.0 Hz, 2H), 7.06 (s, 2H), 6.97 (d, *J* = 9.0 Hz, 2H), 2.36 (s, 9H); ^13^C{^1^H} NMR (101 MHz, CDCl_3_): δ 148.8 (C), 144.9 (C), 143.8 (2 × C), 140.7 (C), 129.9 (2 × CH), 124.9 (2 × CH), 124.8 (C), 124.2 (2 × CH), 21.6 (2 × CH_3_), 21.3 (CH_3_); MS (ESI) *m*/*z* 274 (M + H^+^, 100).

### 1*H*-Indol-3-yl(4′-nitrophenyl)sulfane (**7h**)^30^

The reaction was performed as described in general procedure C using indole (0.0269 g, 0.230 mmol) and *N*-(4-nitrophenylthio)saccharin (**5b**) (0.100 g, 0.300 mmol). The reaction mixture was stirred at room temperature for 1 h. Purification by flash column chromatography (10–30% diethyl ether in hexane) gave 1*H*-indol-3-yl(4′-nitrophenyl)sulfane (**7h**) (0.0541 g, 87%) as an orange solid. Mp 123–126 °C (lit.^30^ 121–122 °C); ^1^H NMR (400 MHz, CDCl_3_): δ 8.74 (br s, 1H), 8.02–7.97 (m, 2H), 7.57–7.49 (m, 3H), 7.35–7.29 (m, 1H), 7.24–7.18 (m, 1H), 7.17–7.10 (m, 2H); ^13^C{^1^H} NMR (101 MHz, CDCl_3_): δ 150.1 (C), 144.9 (C), 136.7 (C), 131.4 (CH), 128.5 (C), 125.2 (2 × CH), 124.0 (2 × CH), 123.6 (CH), 121.5 (CH), 119.2 (CH), 112.1 (CH), 100.0 (C); MS (ESI) *m*/*z* 271 (M + H^+^, 100).

### 1*H*-Indol-3-yl(5-nitro)(4′-nitrophenyl)sulfane (**7i**)

The reaction was performed as described in general procedure C using 5-nitroindole (0.0374 g, 0.230 mmol) and *N*-(4-nitrophenylthio)saccharin (**5b**) (0.100 g, 0.300 mmol). The reaction mixture was stirred at room temperature for 24 h. Purification by flash column chromatography (0–80% dichloromethane in hexane) gave 1*H*-indol-3-yl(5-nitro)(4′-nitrophenyl)sulfane (**7i**) (0.0492 g, 68%) as a yellow solid. Mp 242–244 °C; IR (neat) 3291, 1577, 1505, 1473, 1330, 1081, 736 cm^–1^; ^1^H NMR (400 MHz, DMSO-*d*_6_): δ 8.23 (d, *J* = 2.2 Hz, 1H), 8.20–8.17 (m, 1H), 8.11 (dd, *J* = 9.0, 2.2 Hz, 1H), 8.10–8.03 (m, 2H), 7.74 (d, *J* = 9.0 Hz, 1H), 7.26–7.18 (m, 2H); ^13^C{^1^H} NMR (101 MHz, DMSO-*d*_6_): δ 148.5 (C), 144.8 (C), 141.9 (C), 140.2 (C), 137.5 (CH), 127.8 (C), 125.3 (2 × CH), 124.2 (2 × CH), 117.9 (CH), 114.6 (CH), 113.6 (C), 100.0 (CH); MS (ESI) *m*/*z* 338 (M + Na^+^, 100); HRMS (ESI) *m*/*z*: [M + Na]^+^ calcd for C_14_H_9_N_3_NaO_4_S 338.0206; found, 338.0206.

### *N*-Acetyl-[2′-(4″-nitrophenylthio)]-l-tryptophan Methyl Ester (**7j**)

The reaction was performed as described in general procedure C using methyl *N*-acetyl-l-tryptophanate (0.060 g, 0.23 mmol) and *N*-(4-nitrophenylthio)saccharin (**5b**) (0.10 g, 0.30 mmol). The reaction mixture was stirred at room temperature for 1.5 h. Purification by flash chromatography (30–60% ethyl acetate in hexane) gave *N*-acetyl-[2′-(4″-nitrophenylthio)]-l-tryptophan methyl ester (**7j**) (0.084 g, 88%) as a yellow solid. Mp 84–90 °C; [α]_D_^20^ +55.1 (*c* 0.1, CHCl_3_); IR (neat) 3248, 1735, 1655, 1576, 1509, 1435, 1334, 740 cm^–1^; ^1^H NMR (400 MHz, CDCl_3_): δ 8.97 (br s, 1H), 8.01–7.95 (m, 2H), 7.61 (d, *J* = 8.2 Hz, 1H), 7.35 (d, *J* = 8.2 Hz, 1H), 7.30–7.22 (m, 1H), 7.19–7.12 (m, 1H), 7.06–6.99 (m, 2H), 6.11 (d, *J* = 8.1 Hz, 1H), 4.90–4.83 (m, 1H), 3.63 (s, 3H), 3.38 (dd, *J* = 14.3, 6.0 Hz 1H), 3.27 (dd, *J* = 14.3, 5.8 Hz, 1H), 1.83 (s, 3H); ^13^C{^1^H} NMR (101 MHz, CDCl_3_): δ 172.2 (C), 170.0 (C), 146.9 (C), 145.7 (C), 137.5 (C), 127.9 (C), 125.8 (2 × CH), 124.5 (CH), 124.3 (2 × CH), 120.6 (CH), 120.2 (C), 119.5 (CH), 119.1 (C), 111.7 (CH), 53.0 (CH), 52.7 (CH_3_), 27.7 (CH_2_), 23.2 (CH_3_); MS (ESI) *m*/*z* 414 (M + H^+^, 100); HRMS (ESI) *m*/*z*: [M + H]^+^ calcd for C_20_H_20_N_3_O_5_S 414.1118; found, 414.1119.

## Data Availability

The data underlying this study are available in the published article and its online Supporting Information.

## Supporting Information Available

^1^H and ^13^C NMR spectra of all compounds (PDF)
