# Supplementary material for: Iron-Catalyzed Thioarylation of Arenes Using Saccharin-Derived Reagents
Source: J Org Chem. 2025 Apr 17;90(16):5564–73. doi: 10.1021/acs.joc.5c00250 (PMC12038848; doi:10.1021/acs.joc.5c00250)

Supporting Information for:

Iron-Catalyzed Thioarylation of Arenes using Saccharin-Derived Reagents

Lachlan J. N. Waddell,† Oluwajuwon A. M. Okunade,† Amy C. Dodds,† Michael C. H. Lok,†

R. Nisha Khanizeman‡ and Andrew Sutherland*†

†School of Chemistry, The Joseph Black Building, University of Glasgow, Glasgow G12 8QQ, United Kingdom. ‡GSK Medicines Research Centre, Gunnels Wood Road, Stevenage, SG1 2NY, UK. Email: Andrew.Sutherland@glasgow.ac.uk

Table of Contents

1. ^{1}H and ^{13}C NMR Spectra of all Compounds S2–S61

1. ^{1}H and ^{13}C NMR Spectra of all Compounds

^{1}H NMR (400 MHz, CDCl$_3$)

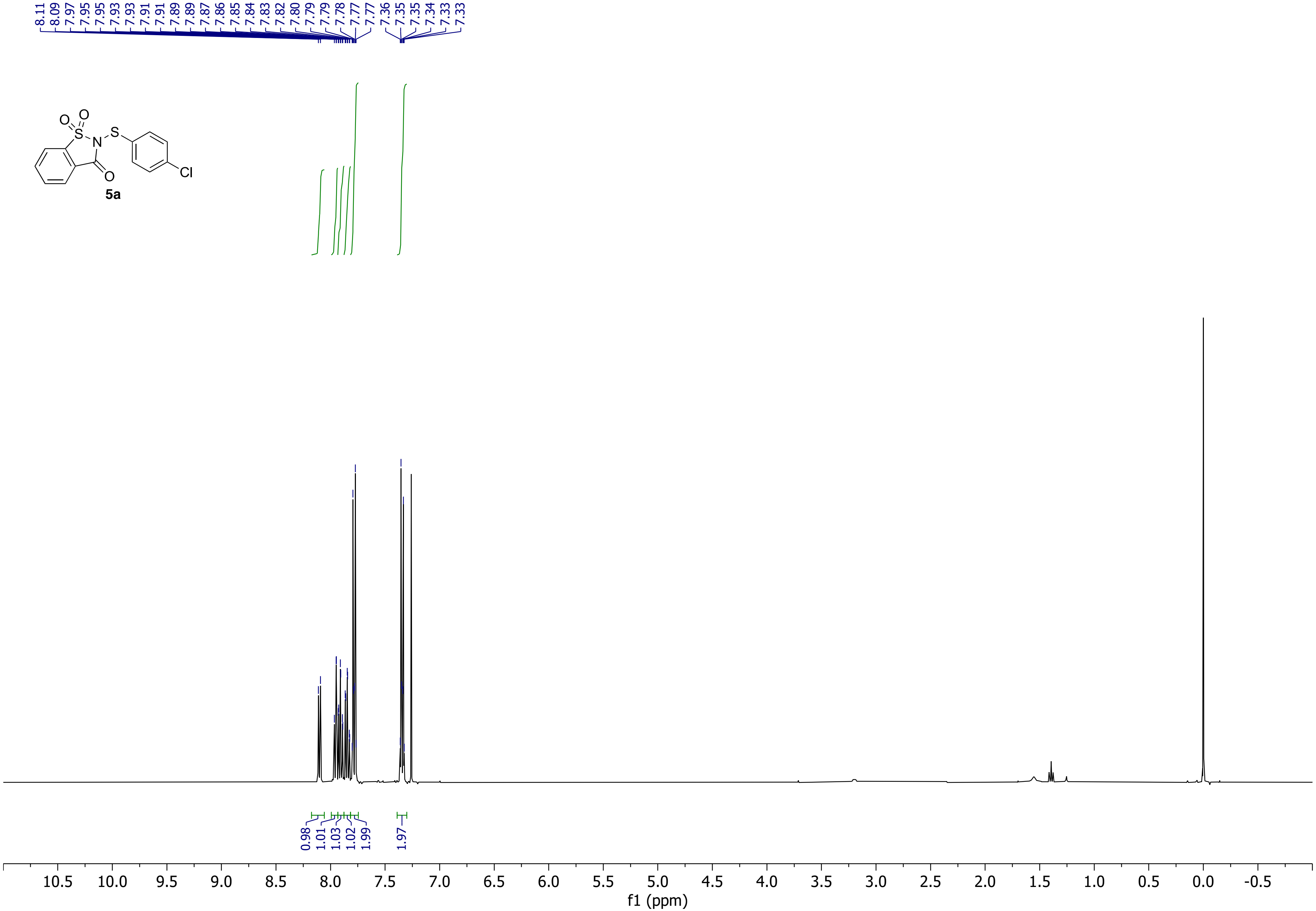

$^{13}C\{^1H\}$ NMR (101 MHz, $CDCl_3$)

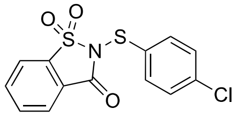

5a

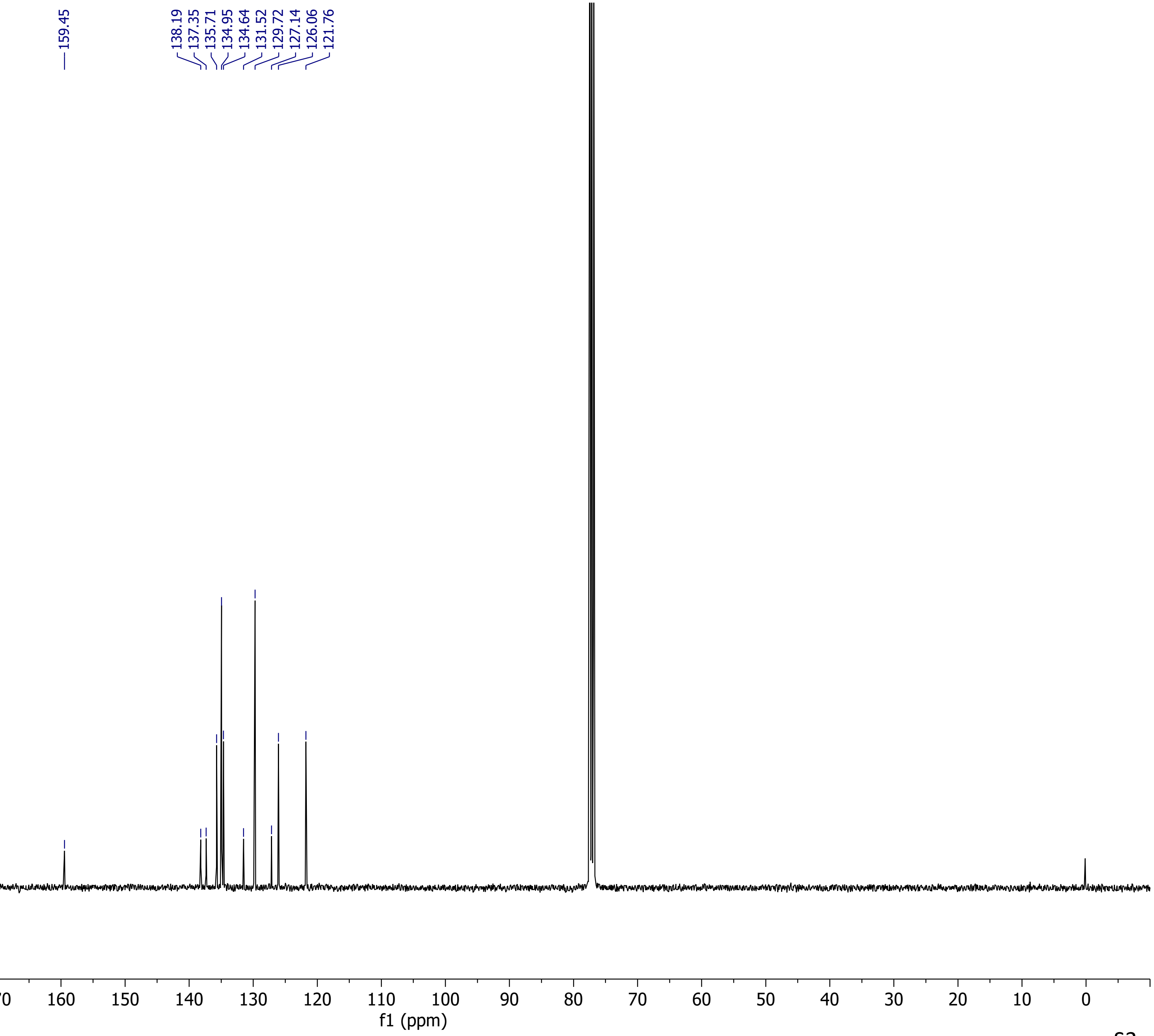

^{1}H NMR (400 MHz, CDCl$_3$)

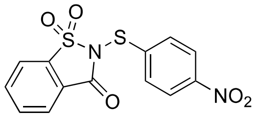

5b

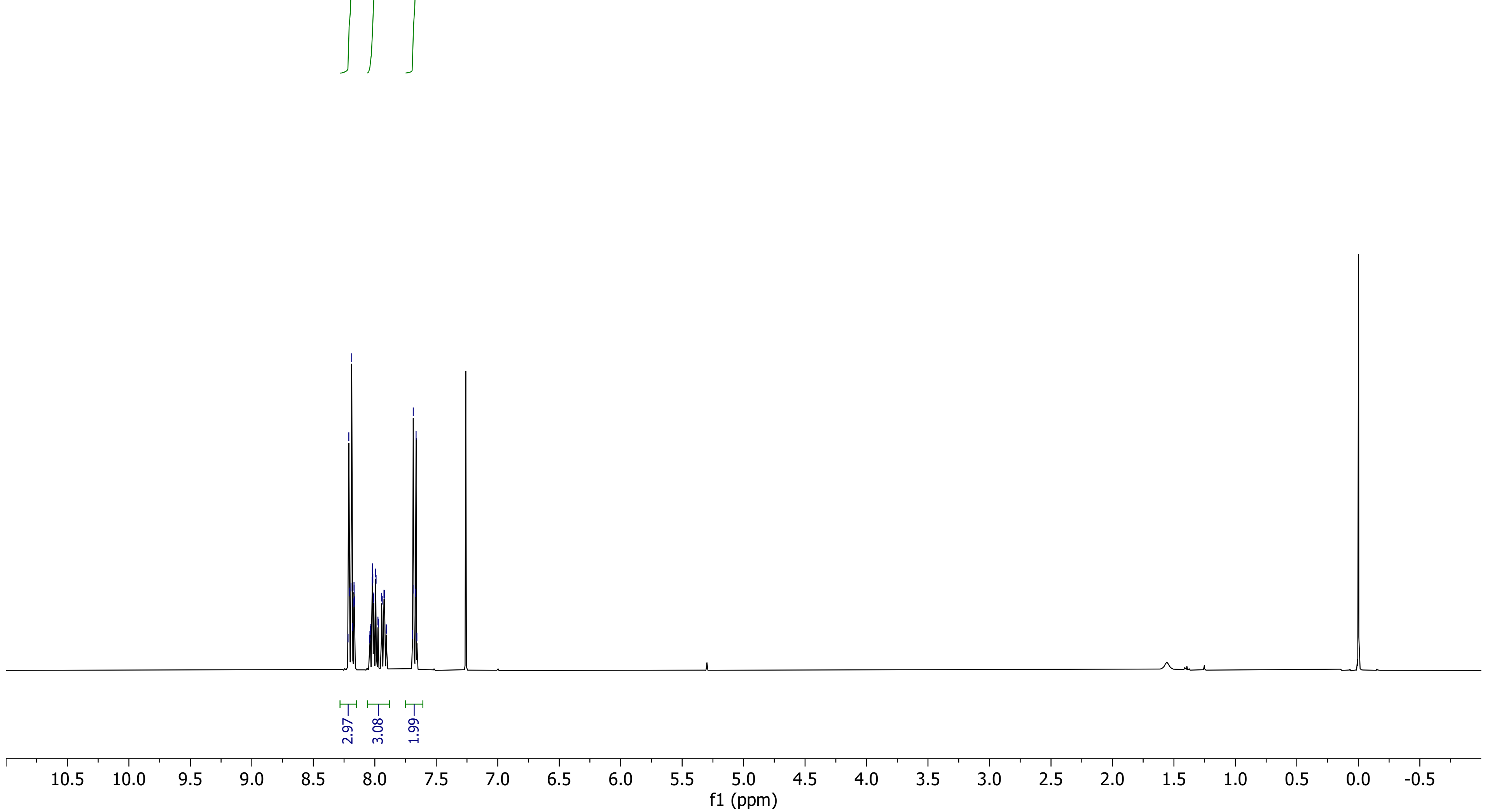

$^{13}C\{^{1}H\}$ NMR (101 MHz, $CDCl_3$)

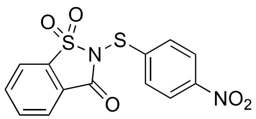

5b

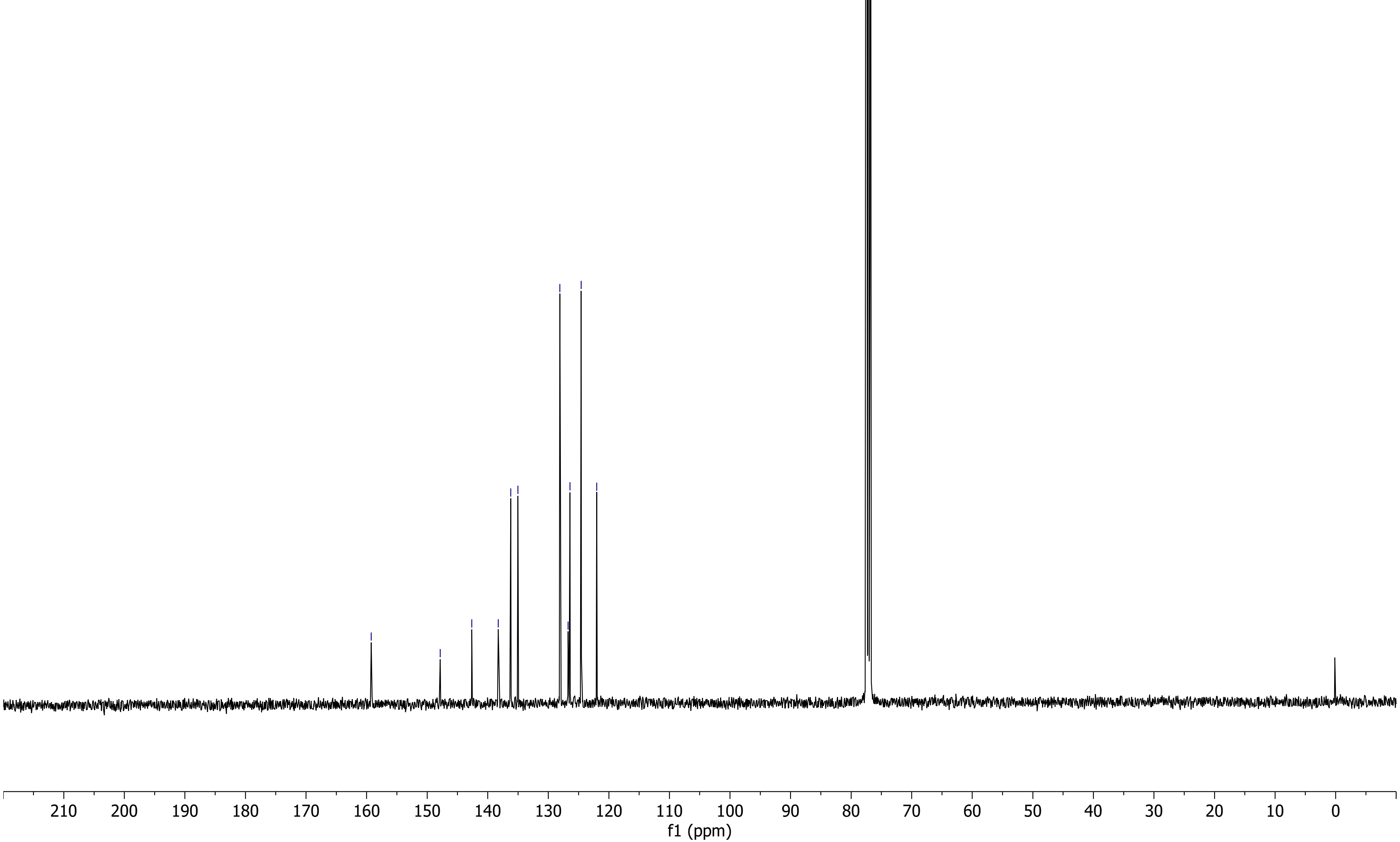

^{1}H NMR (400 MHz, $CDCl_3$)

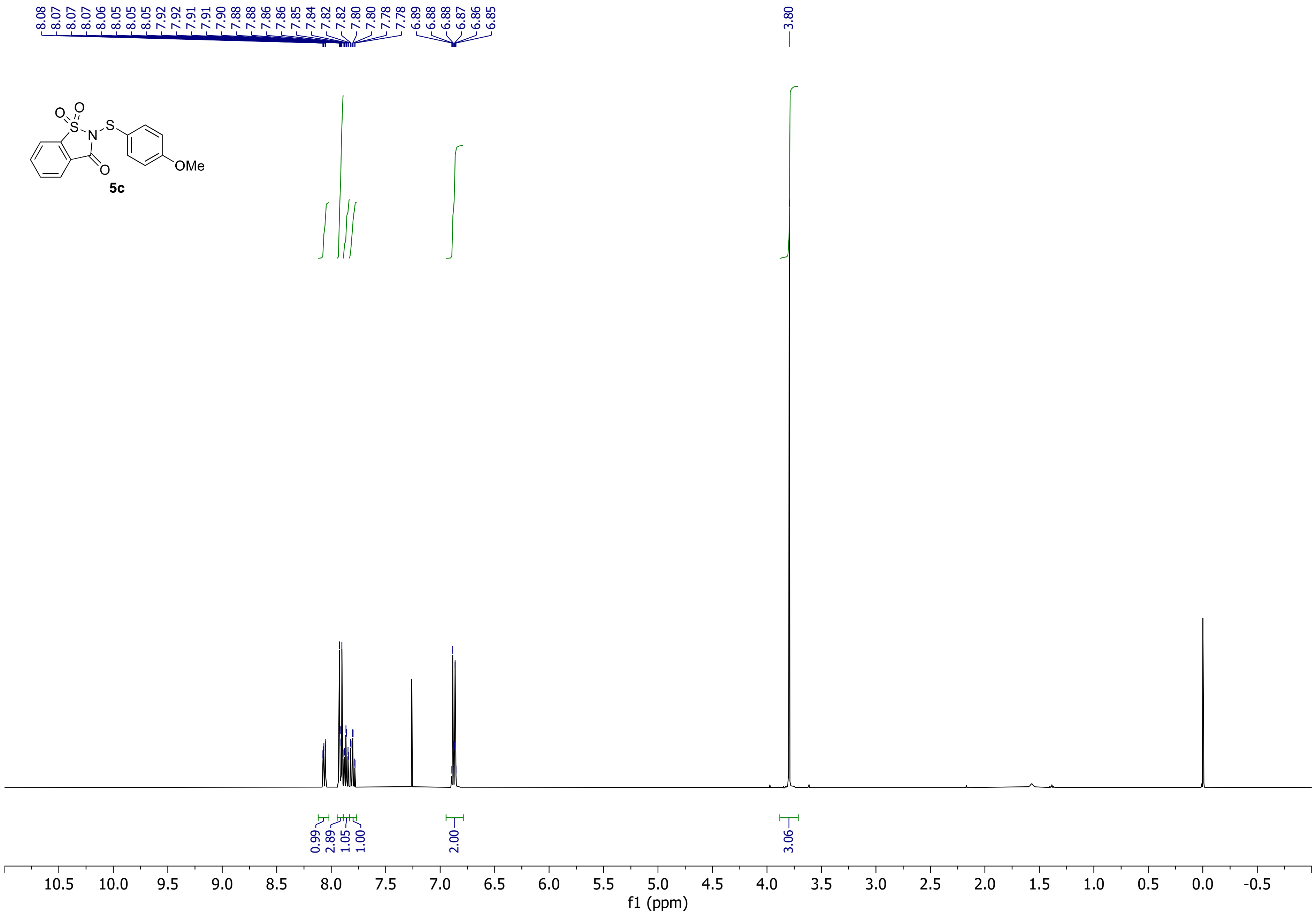

$^{13}C\{^{1}H\}$ NMR (101 MHz, $CDCl_3$)

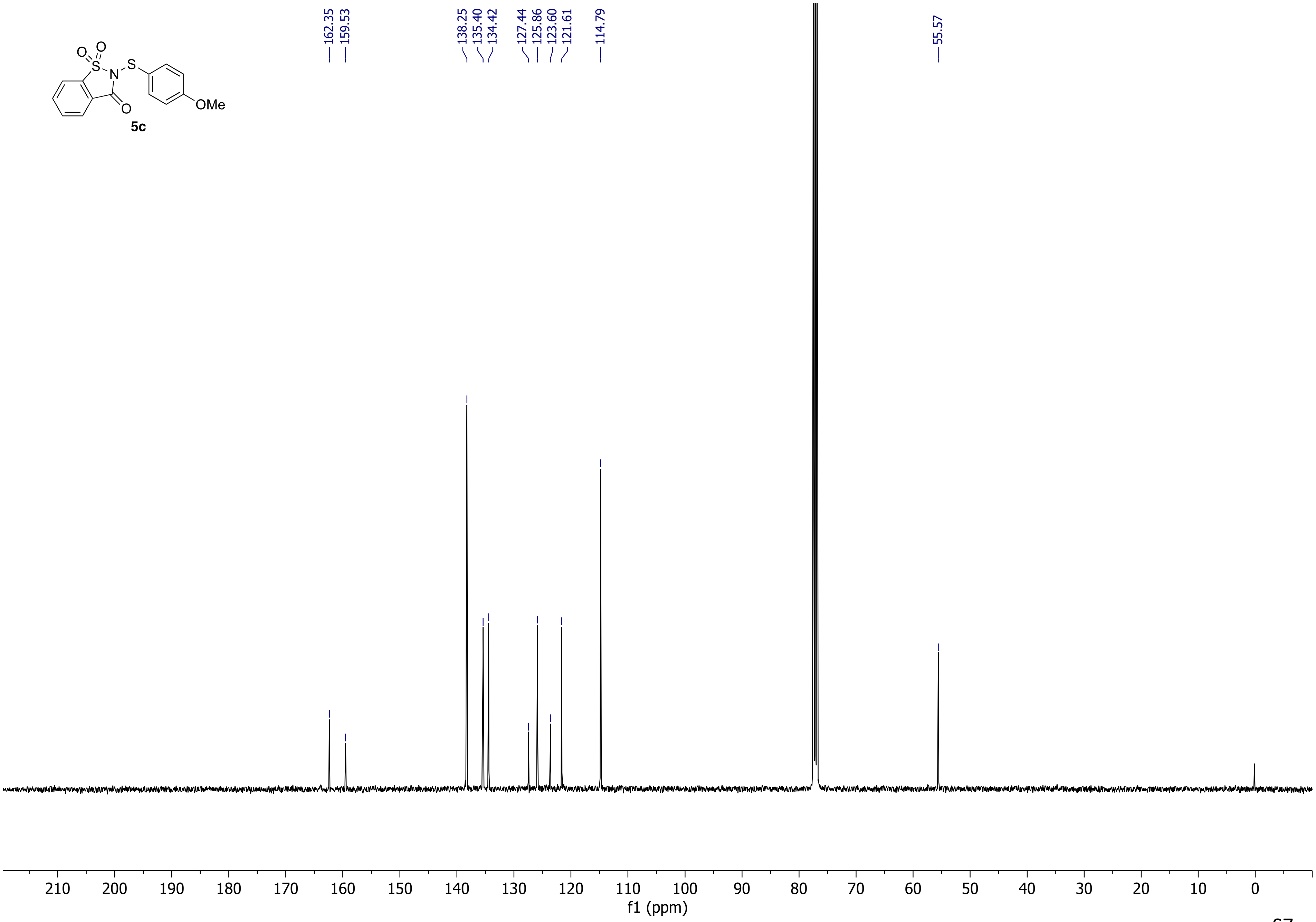

^{1}H NMR (400 MHz, $CDCl_3$)

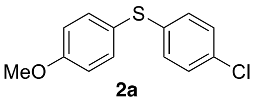

2a

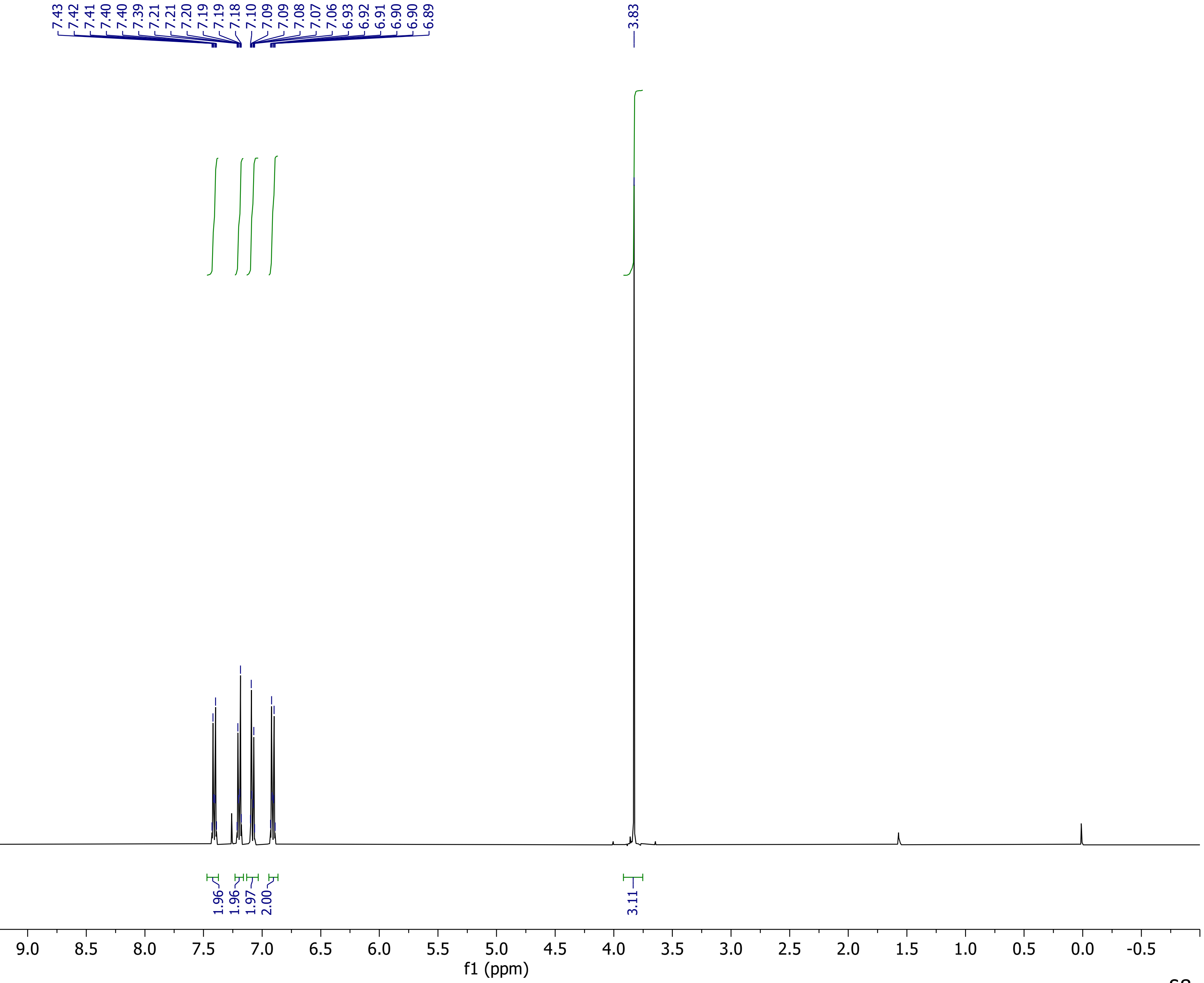

$^{13}C\{^{1}H\}$ NMR (101 MHz, $CDCl_3$)

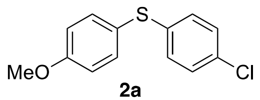

2a

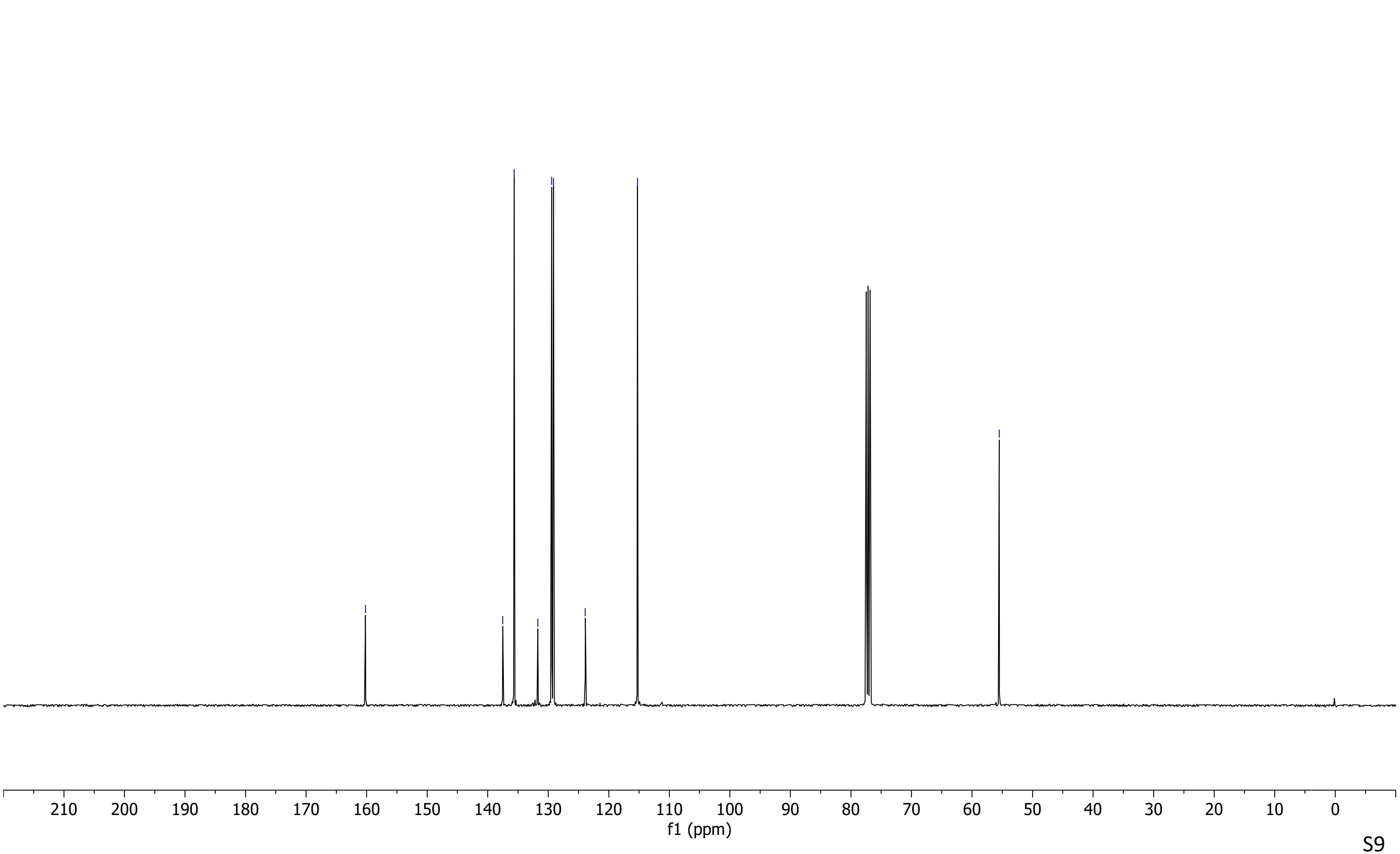

^{1}H NMR (400 MHz, $CDCl_3$)

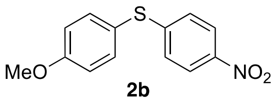

2b

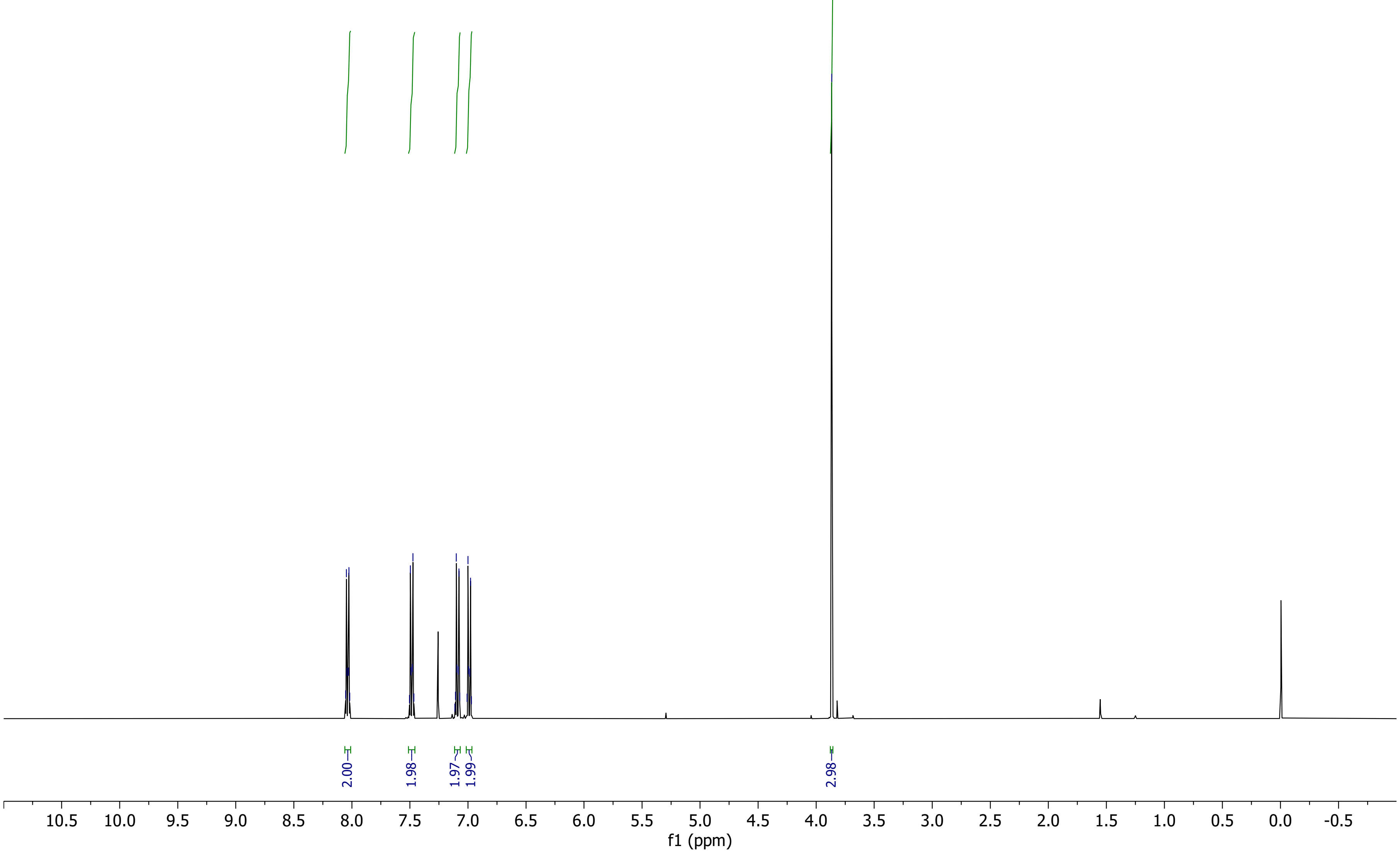

$^{13}C\{^{1}H\}$ NMR (101 MHz, $CDCl_3$)

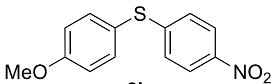

2b

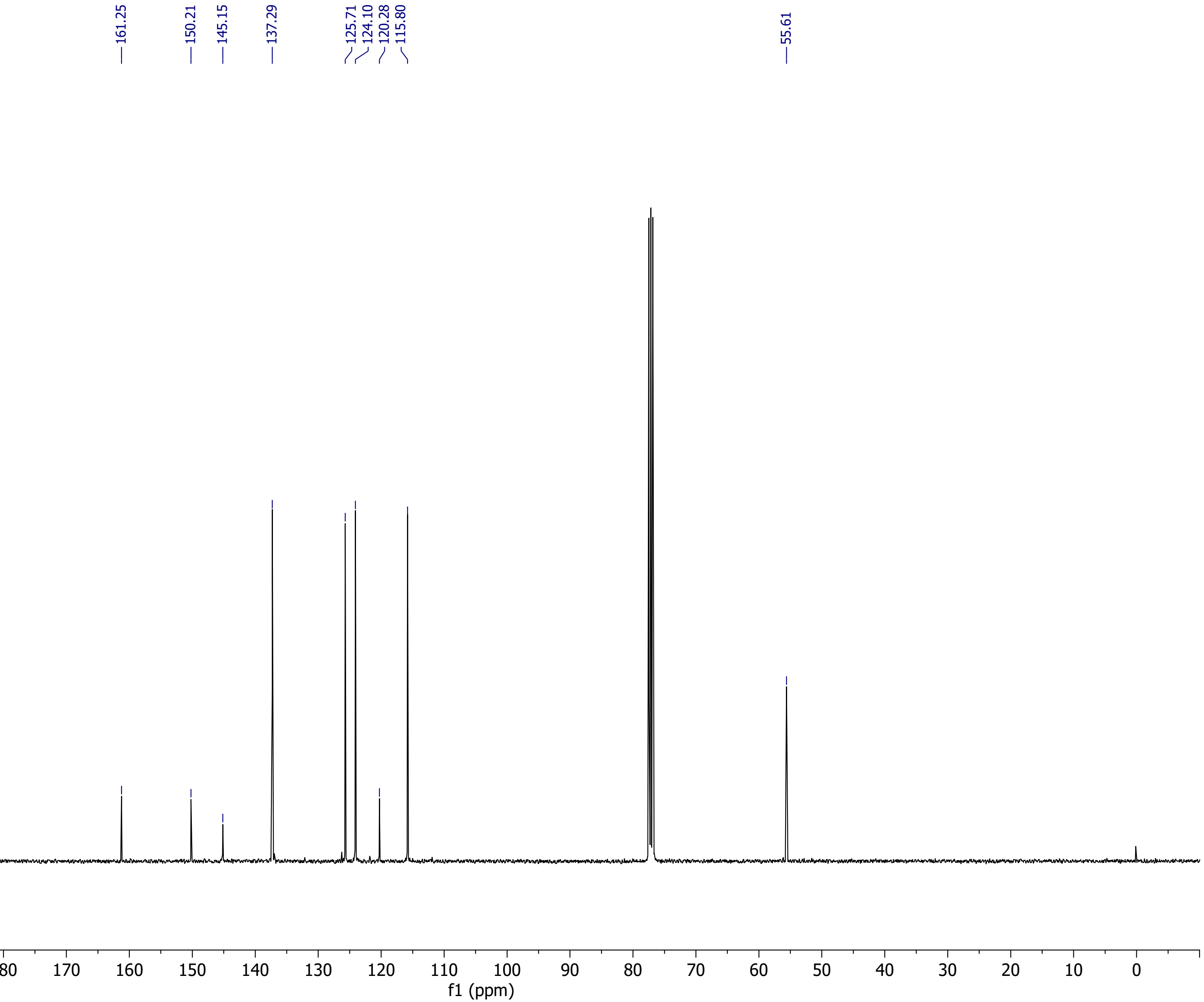

^{1}H NMR (500 MHz, $CDCl_3$)

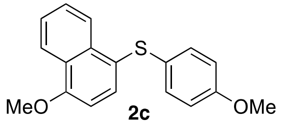

2c

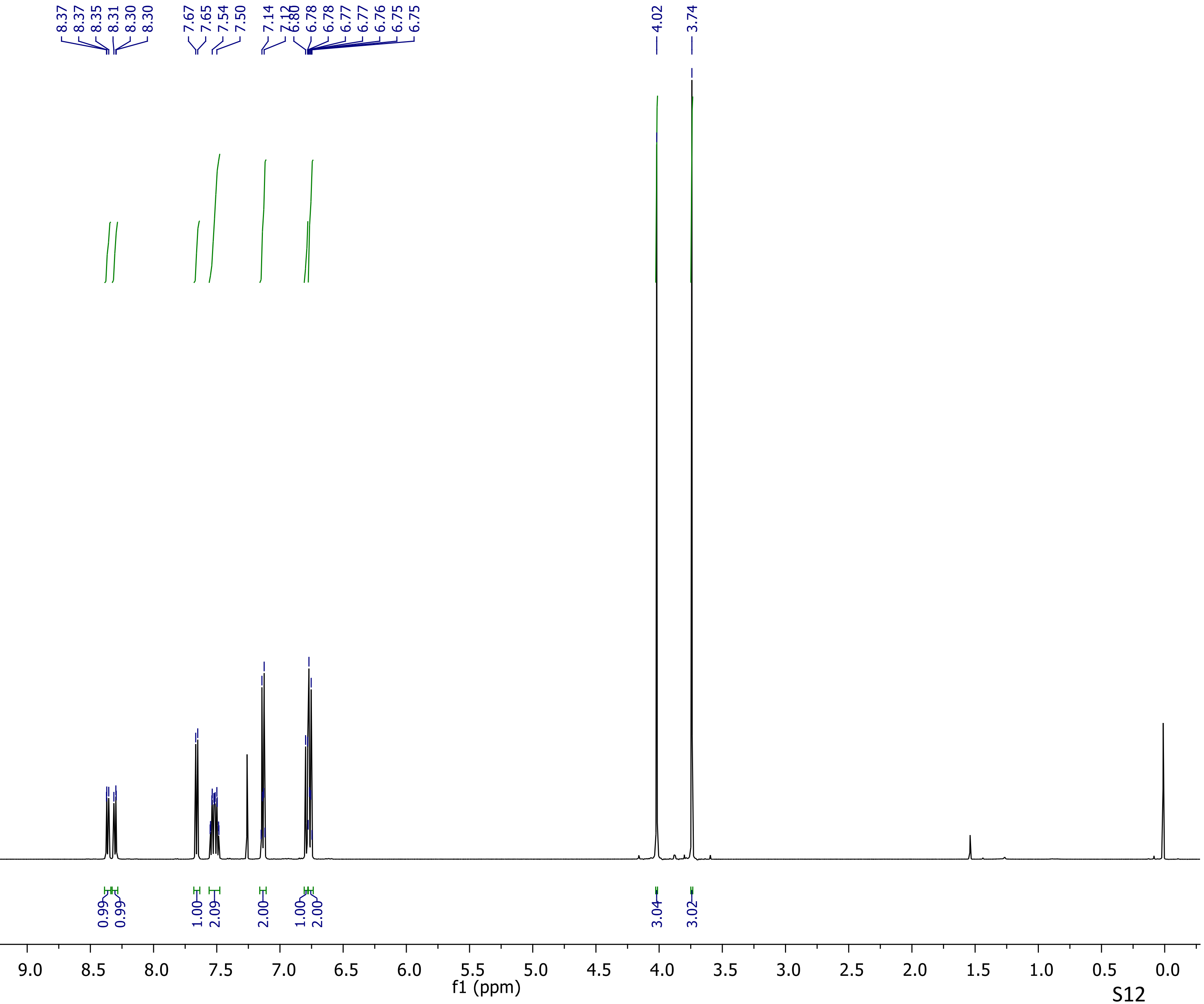

$^{13}C\{^{1}H\}$ NMR (126 MHz, $CDCl_3$)

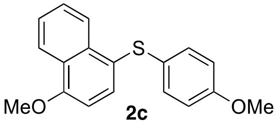

2c

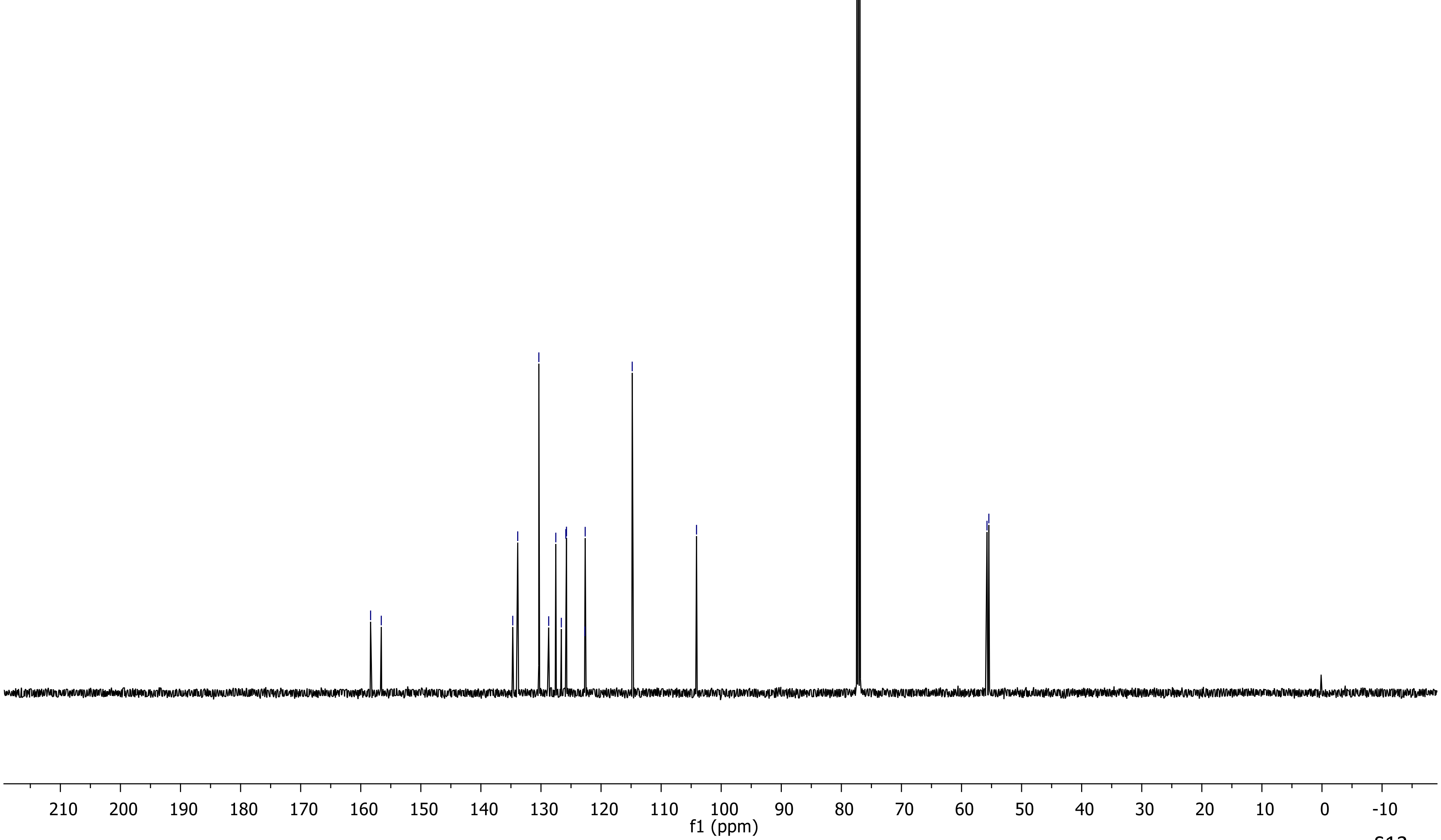

^{1}H NMR (400 MHz, $CDCl_3$)

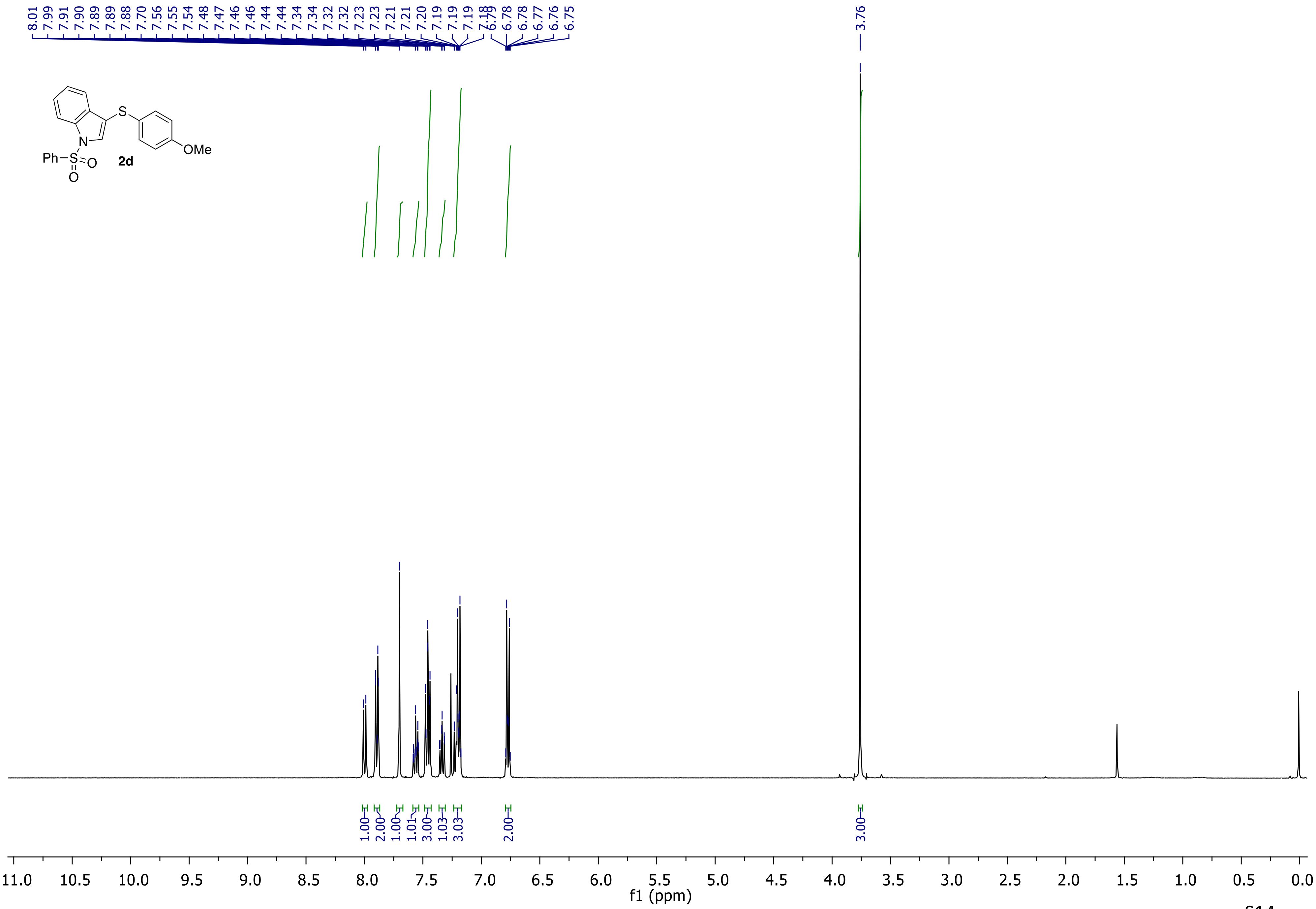

$^{13}C\{^{1}H\}$ NMR (101 MHz, $CDCl_3$)

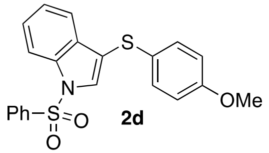

2d

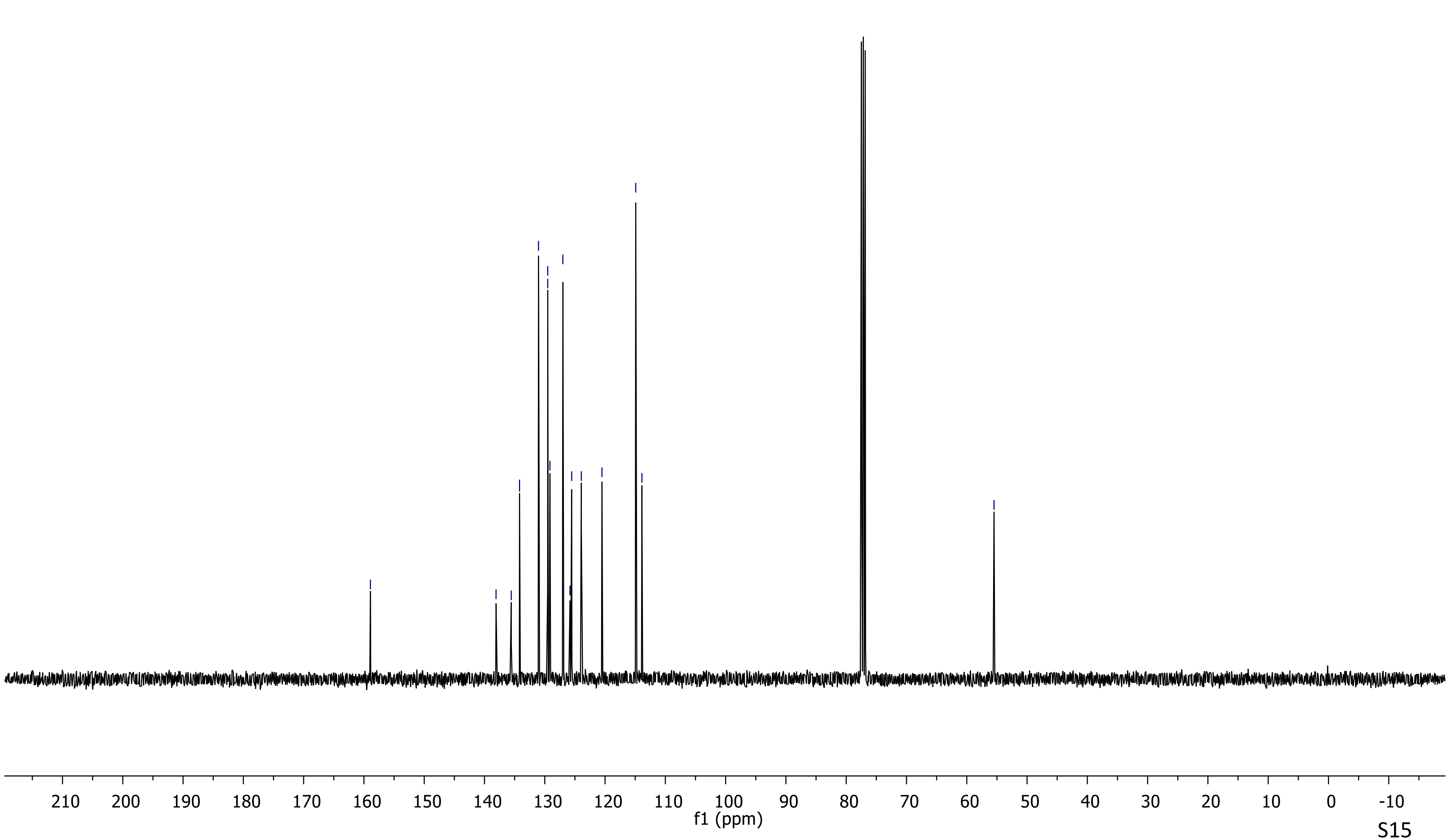

^{1}H NMR (400 MHz, $CDCl_3$)

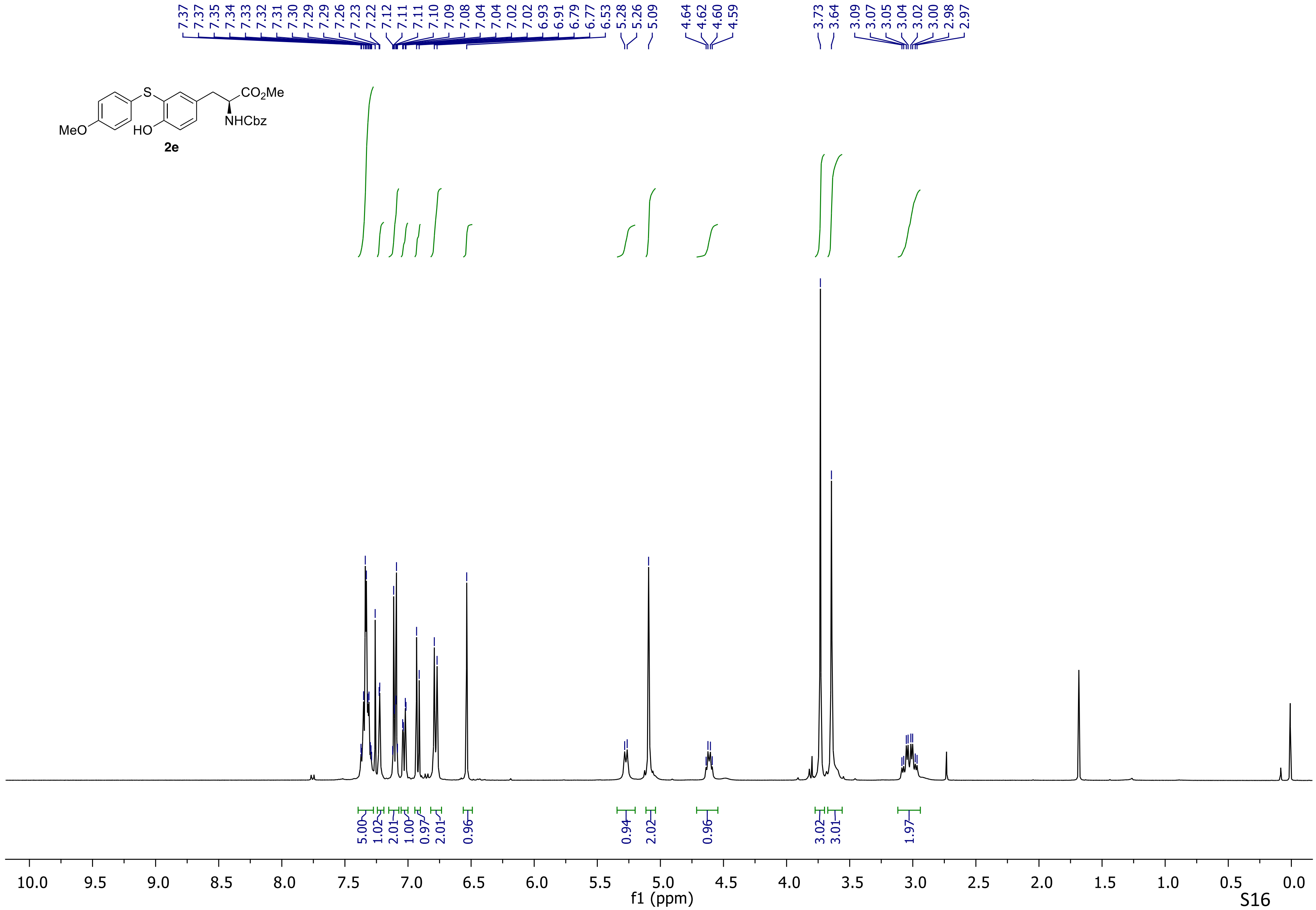

$^{13}C\{^{1}H\}$ NMR (101 MHz, CDCl$_3$)

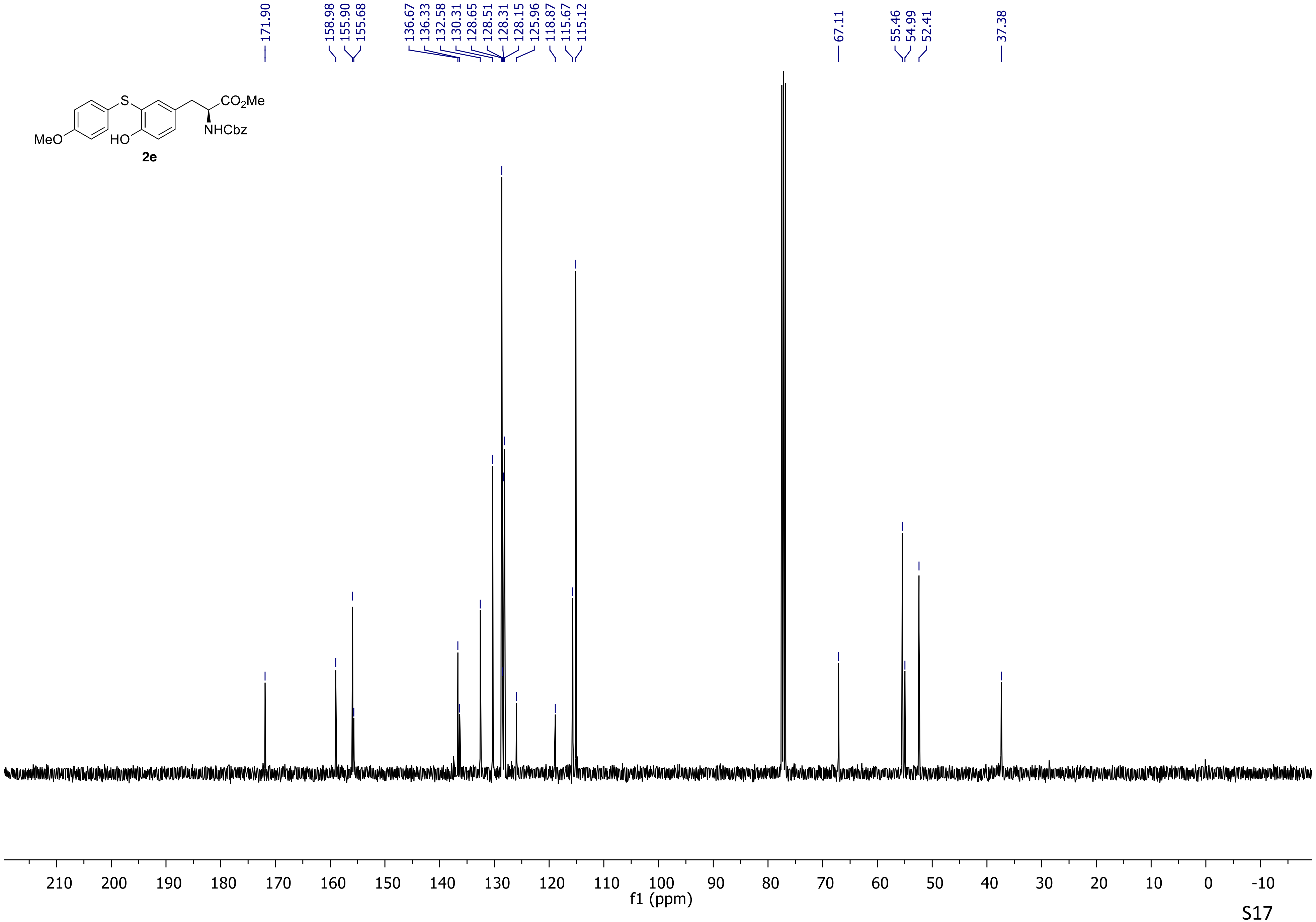

^{1}H NMR (400 MHz, CDCl$_3$)

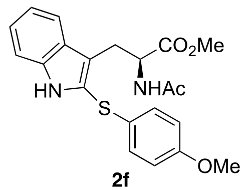

2f

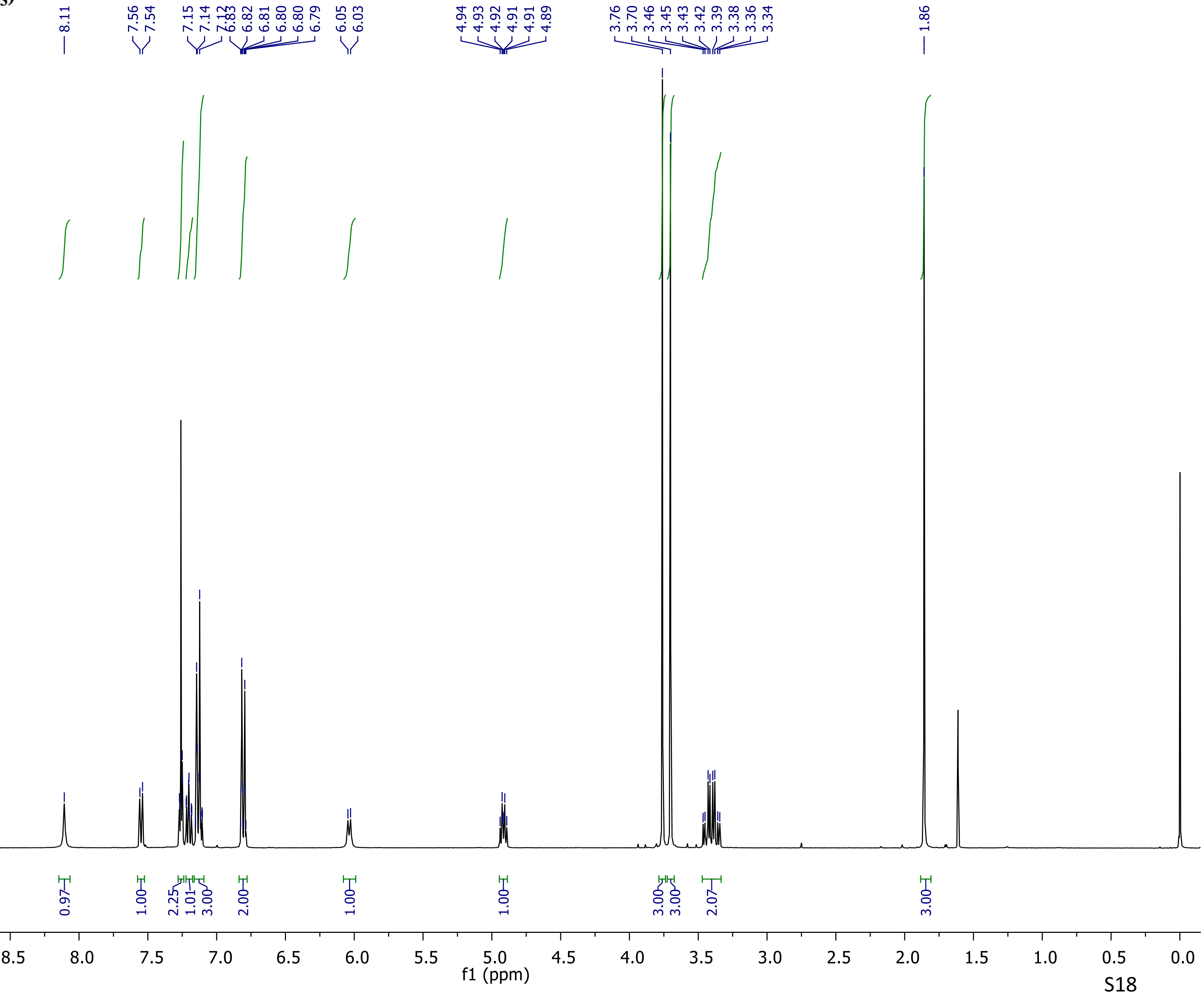

$^{13}C\{^1H\}$ NMR (101 MHz, $CDCl_3$)

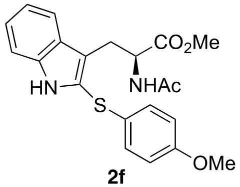

2f

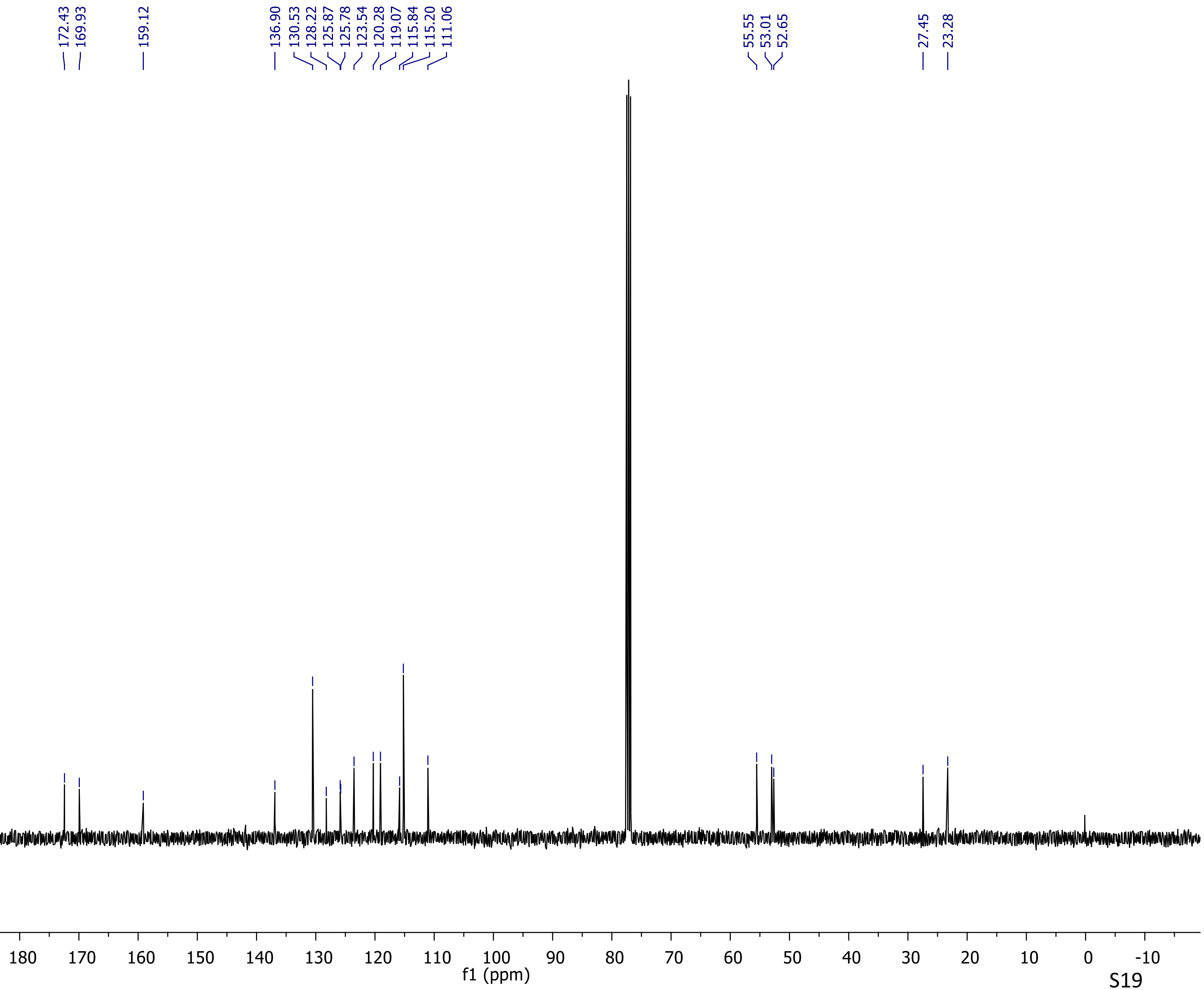

^{1}H NMR (400 MHz, $CDCl_3$)

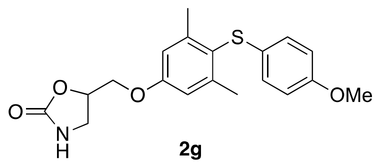

2g

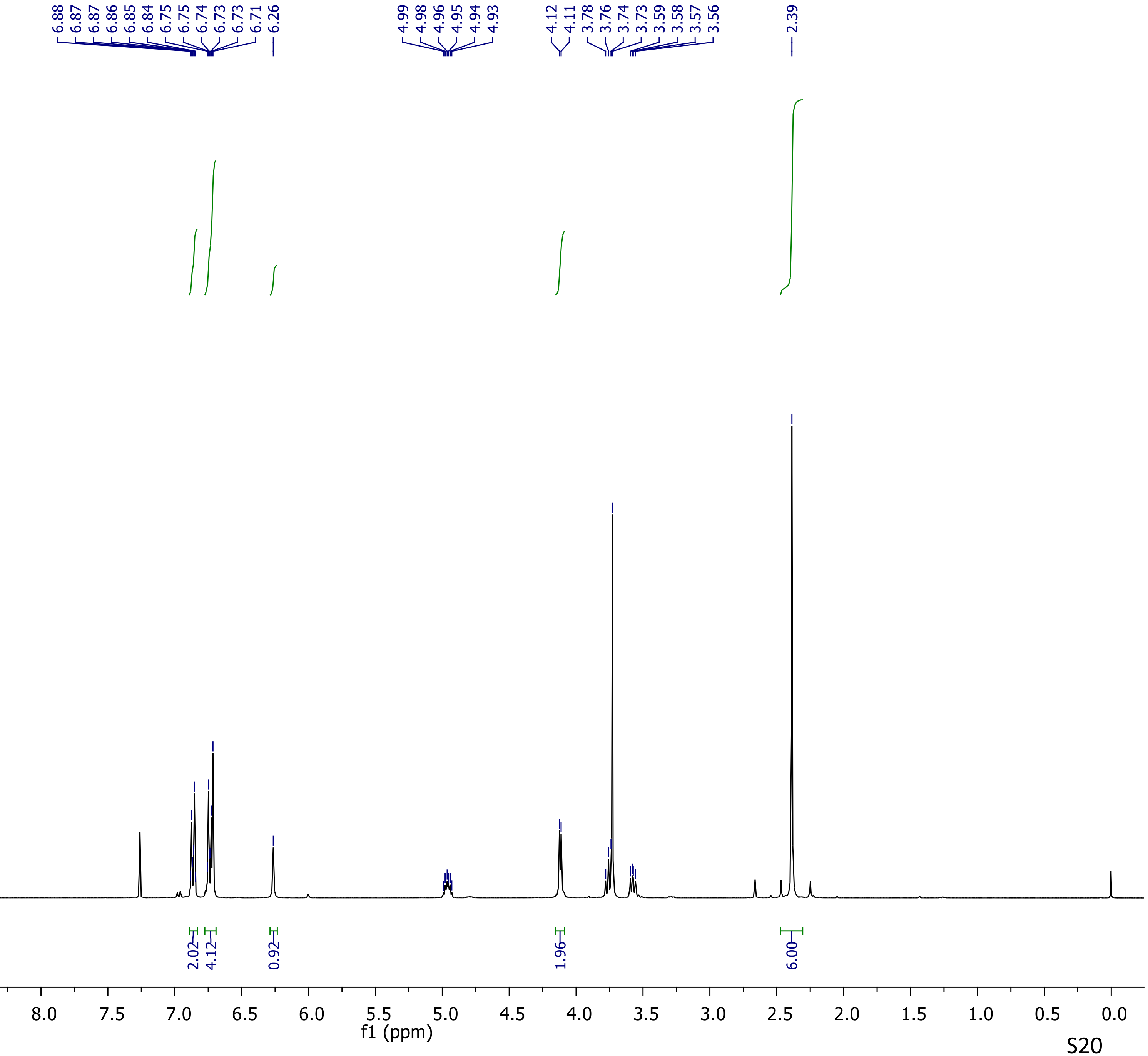

$^{13}C\{^1H\}$ NMR (101 MHz, $CDCl_3$)

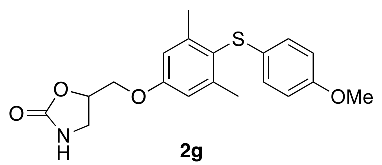

2g

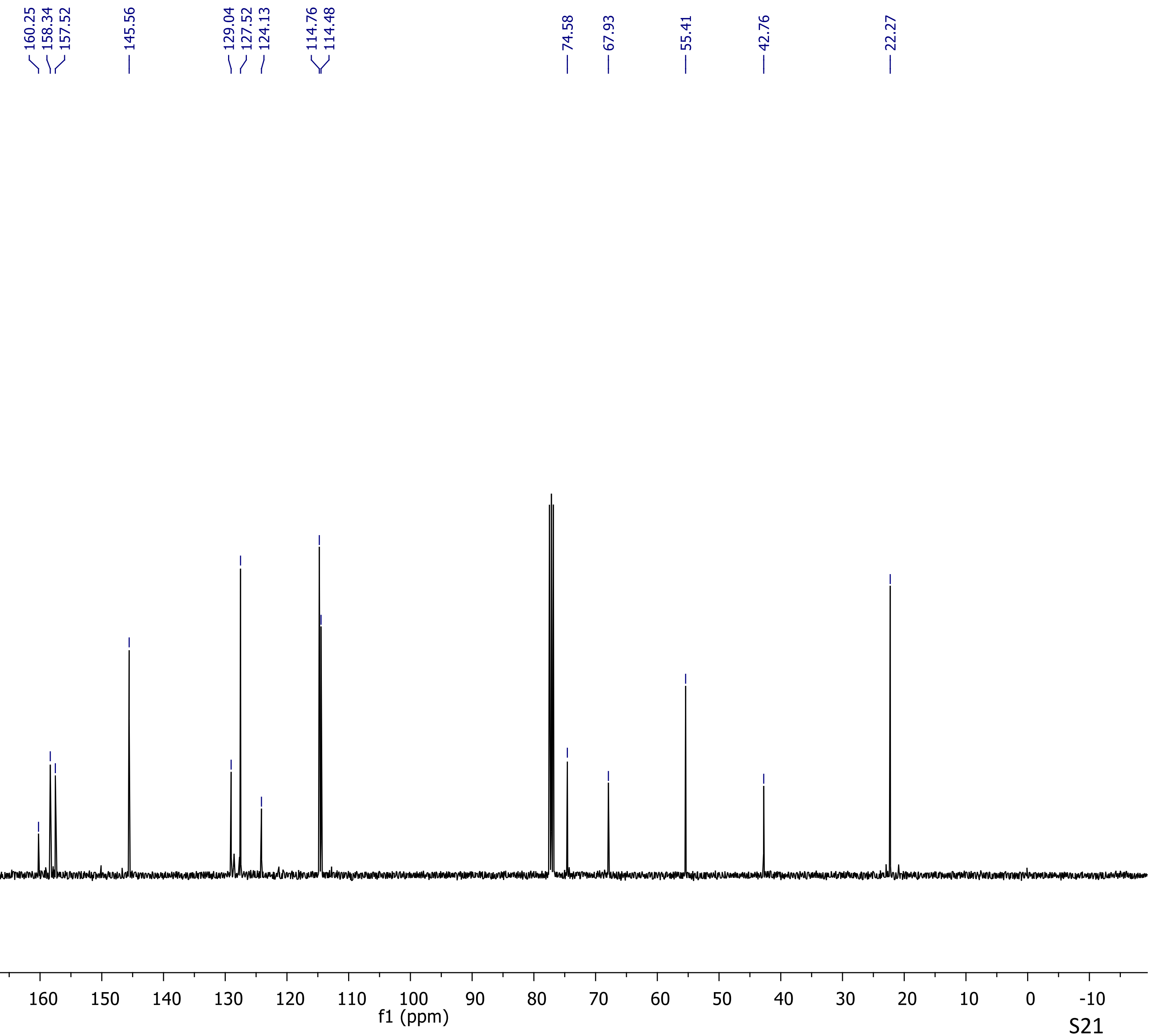

^{1}H NMR (400 MHz, $CDCl_3$)

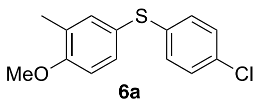

6a

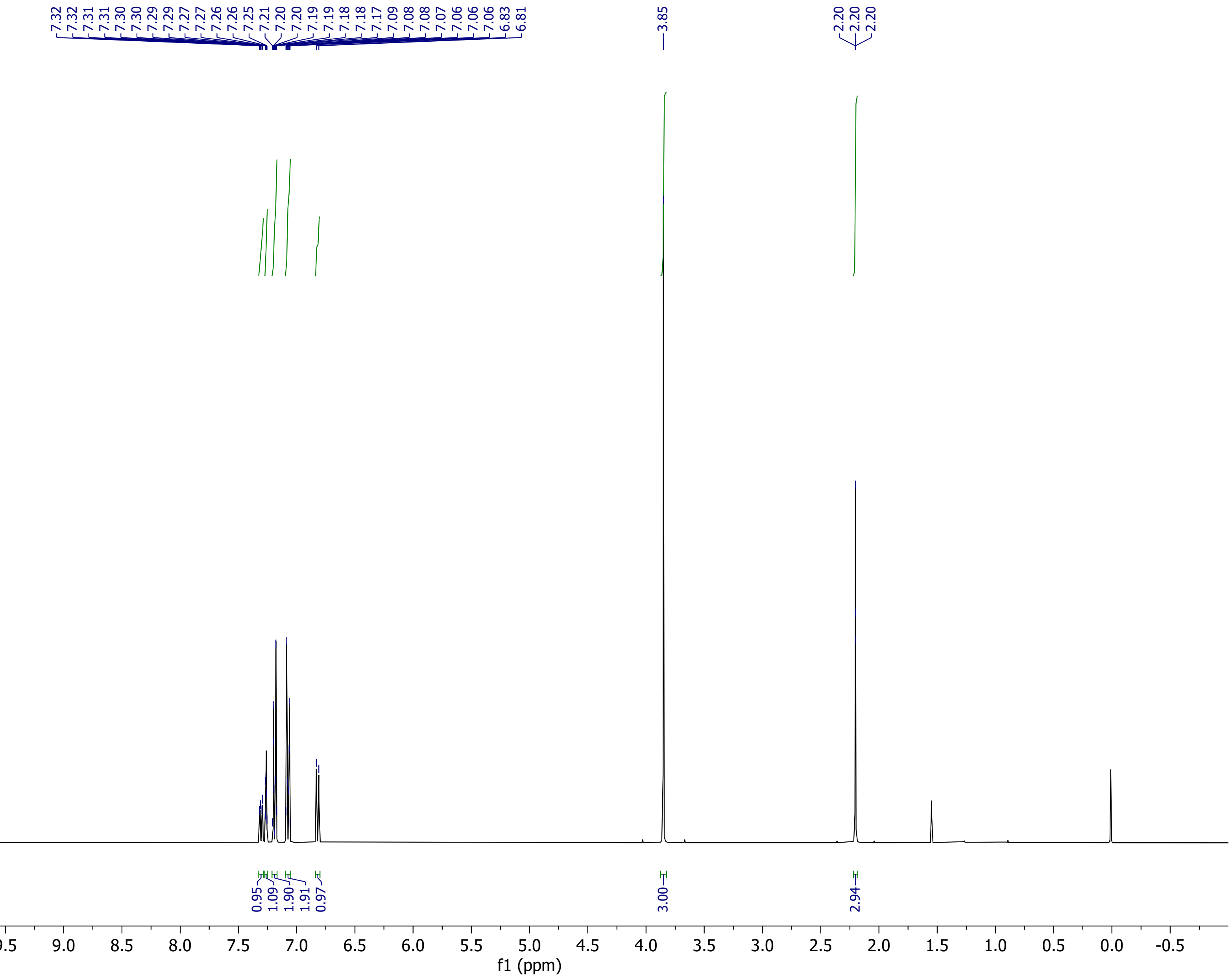

$^{13}C\{^{1}H\}$ NMR (101 MHz, $CDCl_3$)

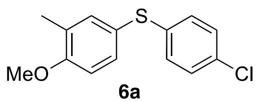

6a

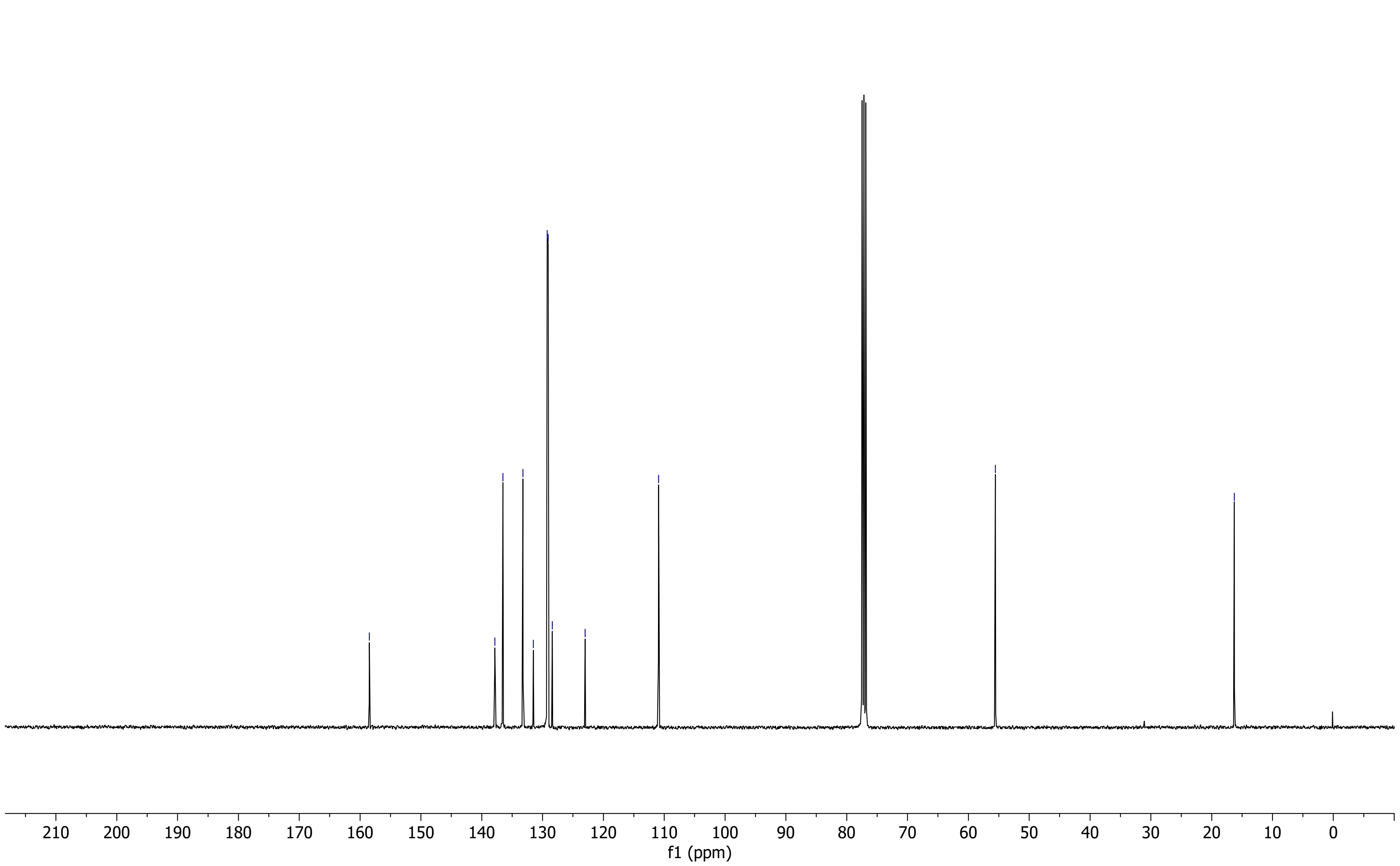

^{1}H NMR (400 MHz, $CDCl_3$)

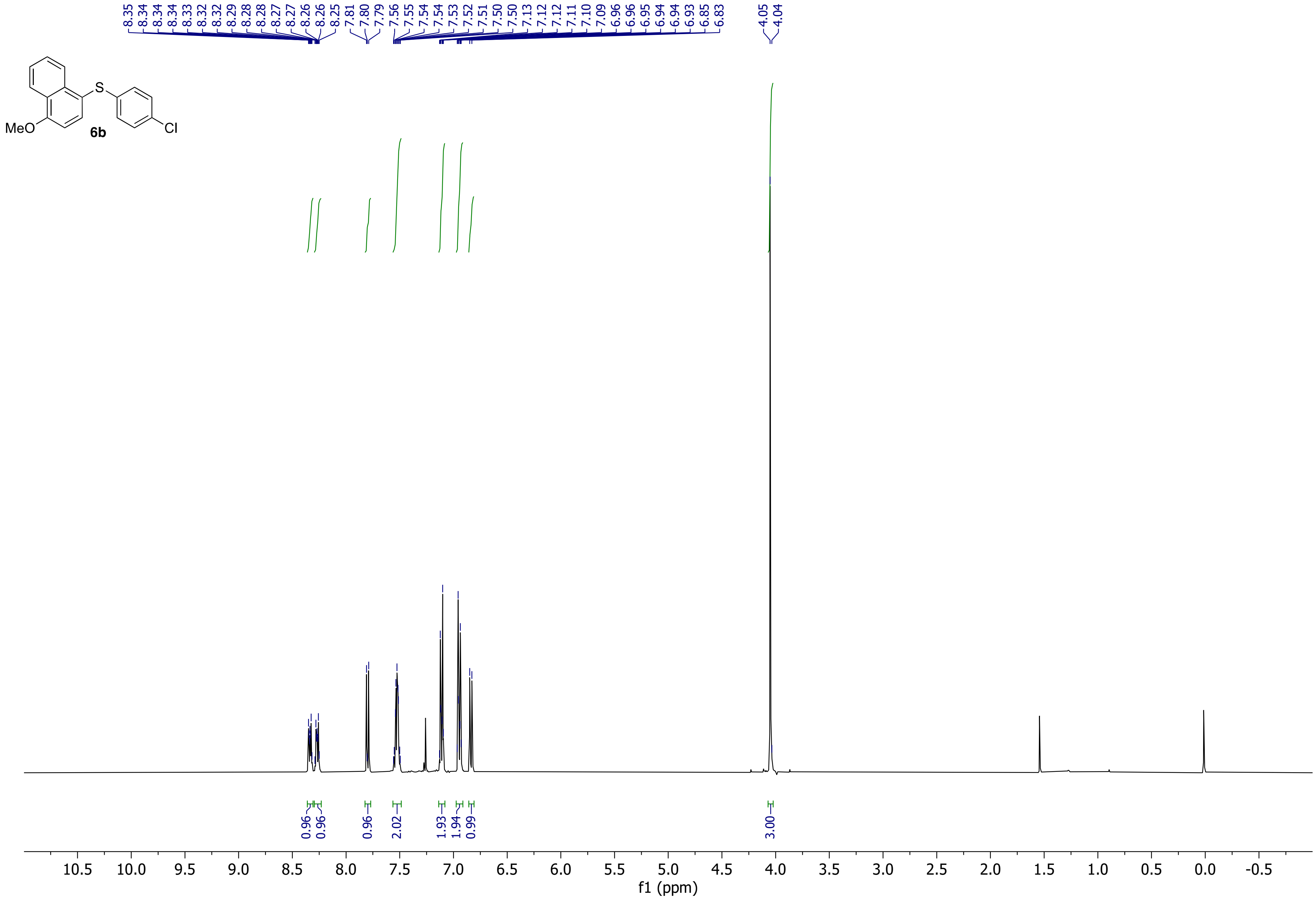

$^{13}C\{^{1}H\}$ NMR (101 MHz, $CDCl_3$)

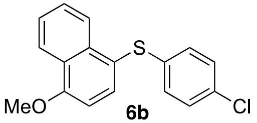

6b

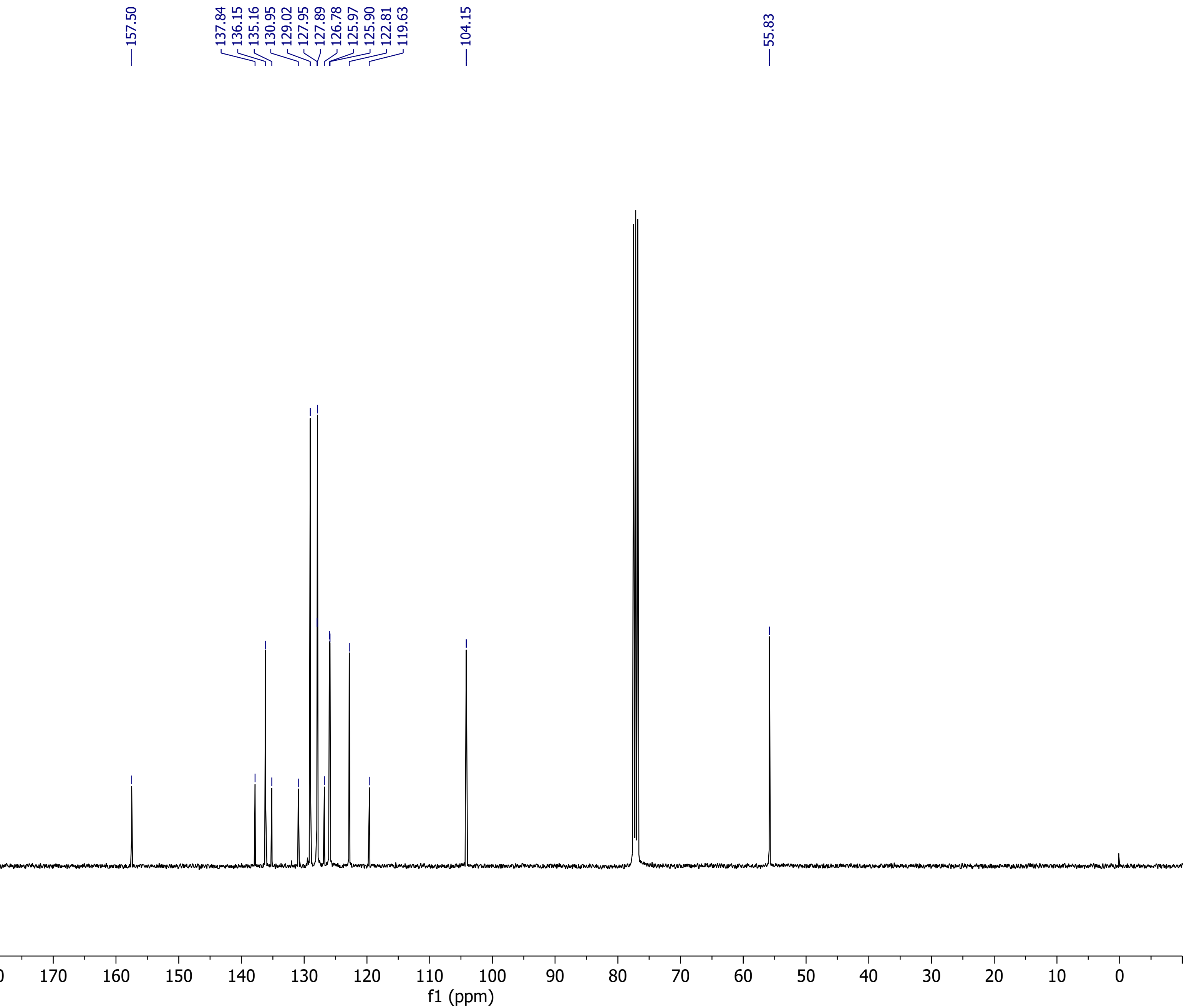

^{1}H NMR (400 MHz, $CDCl_3$)

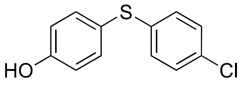

6c

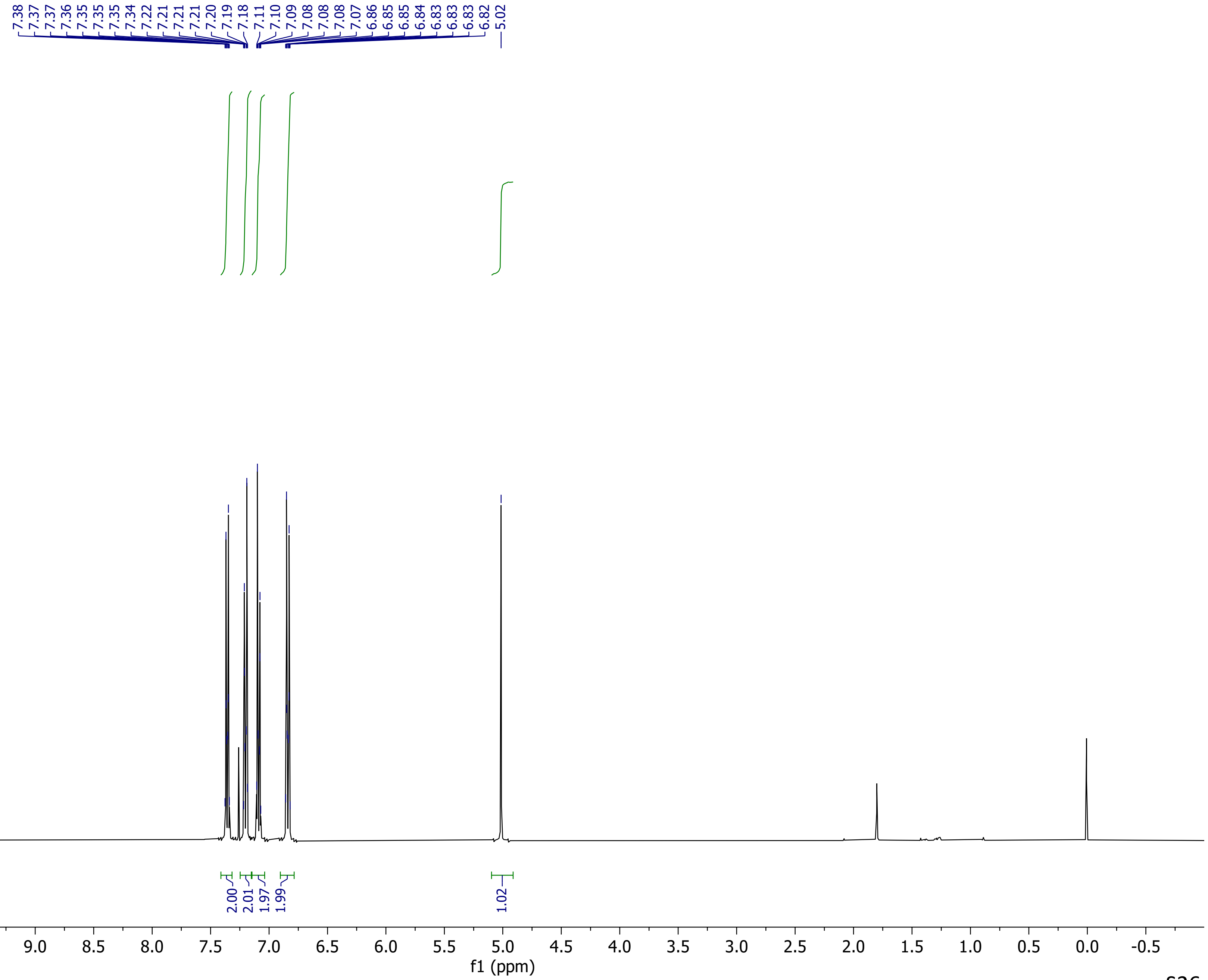

$^{13}C\{^1H\}$ NMR (101 MHz, $CDCl_3$)

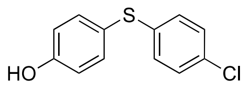
6c

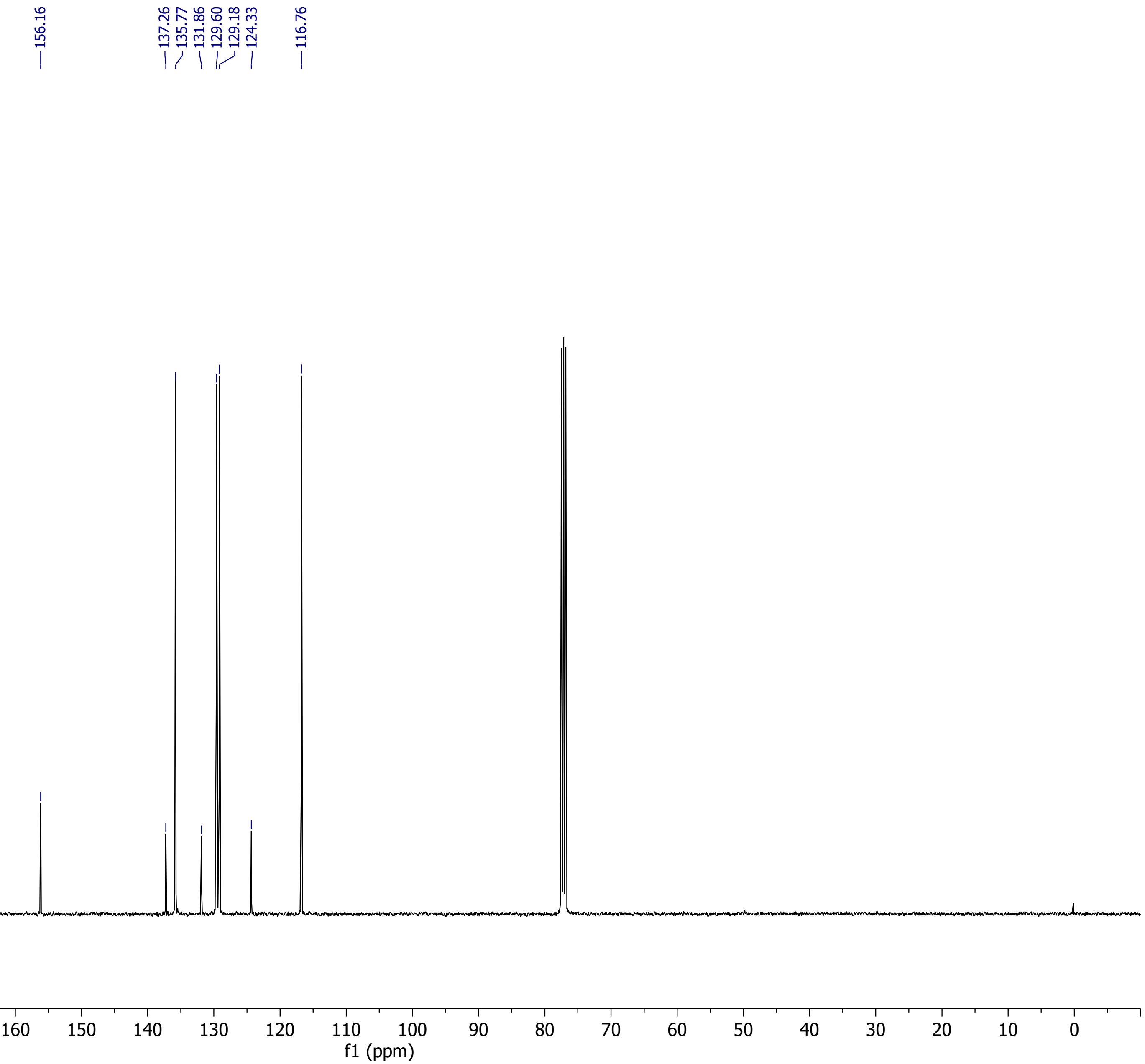

^{1}H NMR (400 MHz, $CDCl_3$)

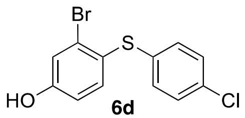

6d

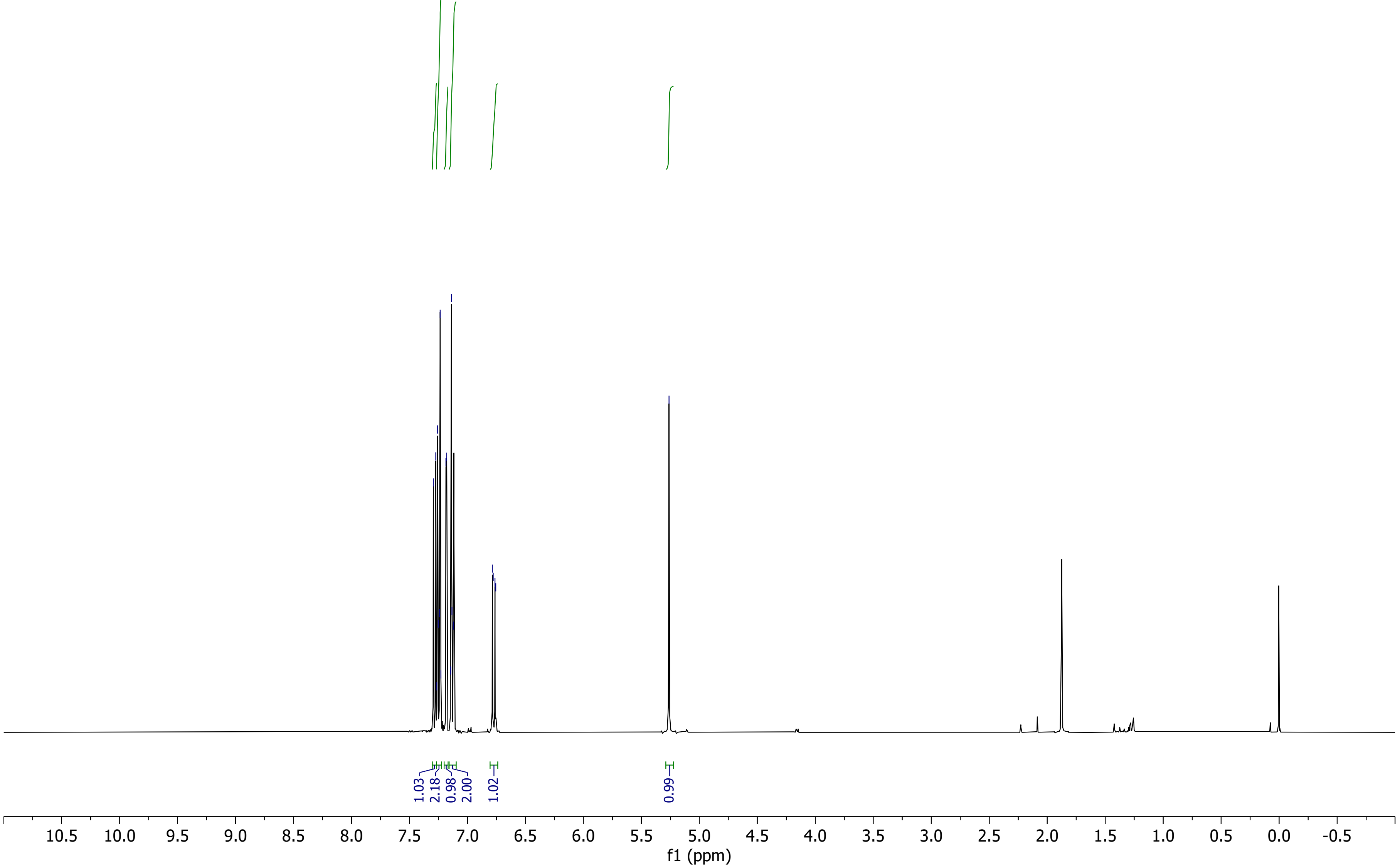

$^{13}C\{^{1}H\}$ NMR (101 MHz, $CDCl_3$)

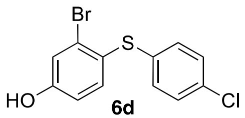

6d

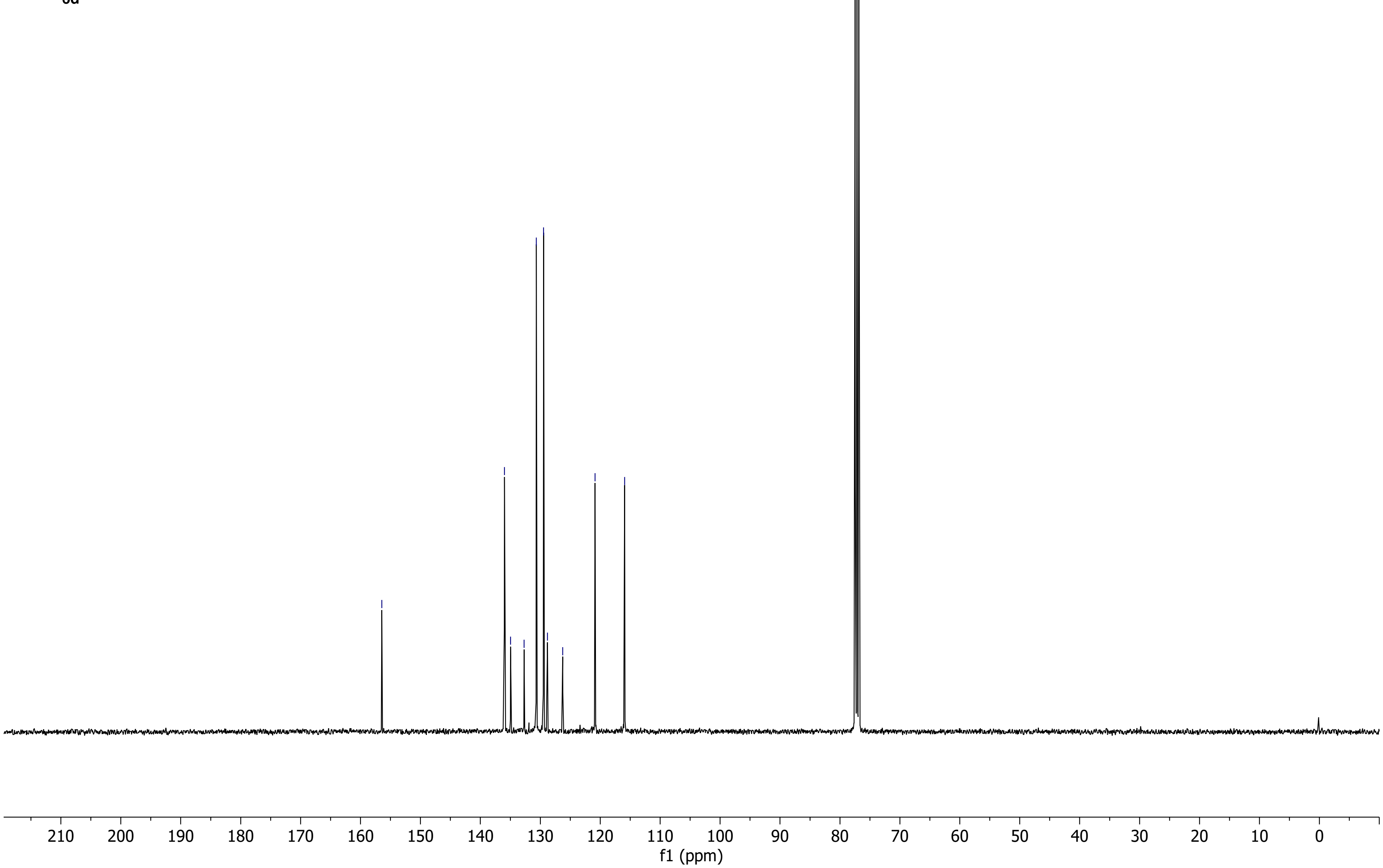

^{1}H NMR (400 MHz, $CDCl_3$)

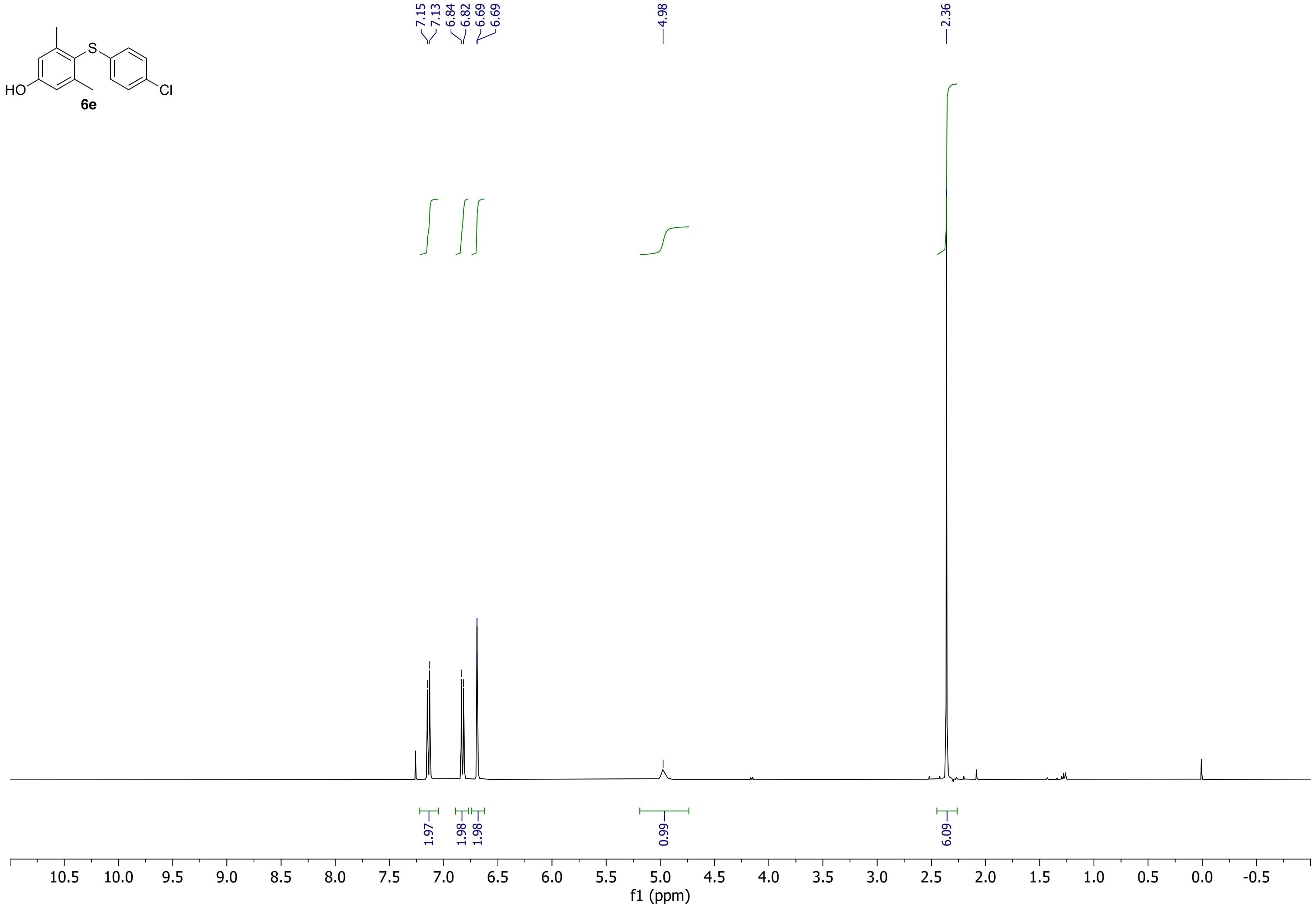

$^{13}C\{^{1}H\}$ NMR (101 MHz, $CDCl_3$)

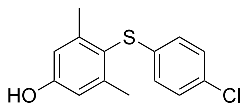

6e

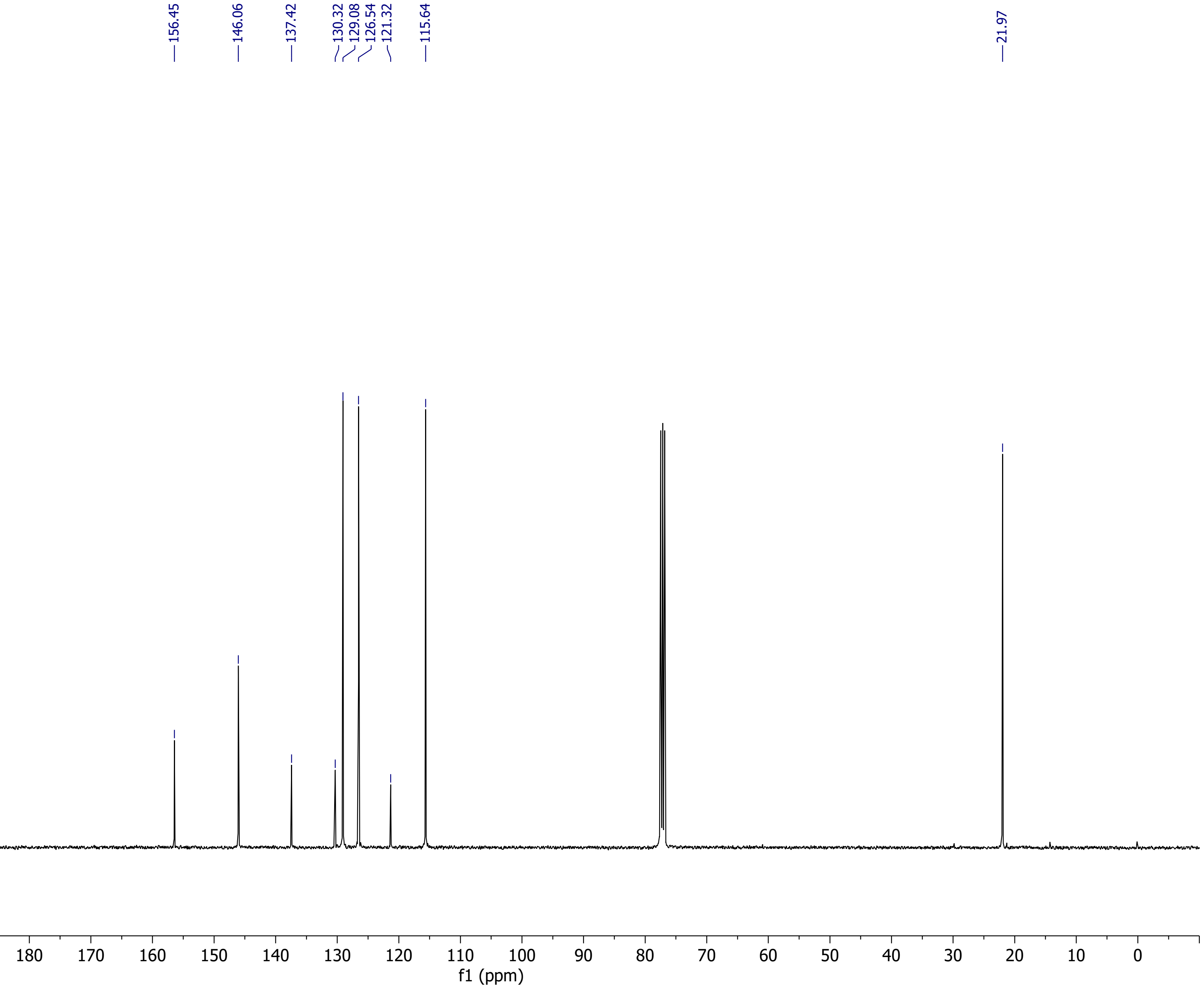

^{1}H NMR (400 MHz, CDCl$_3$)

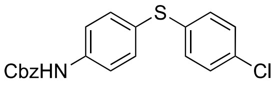

6f

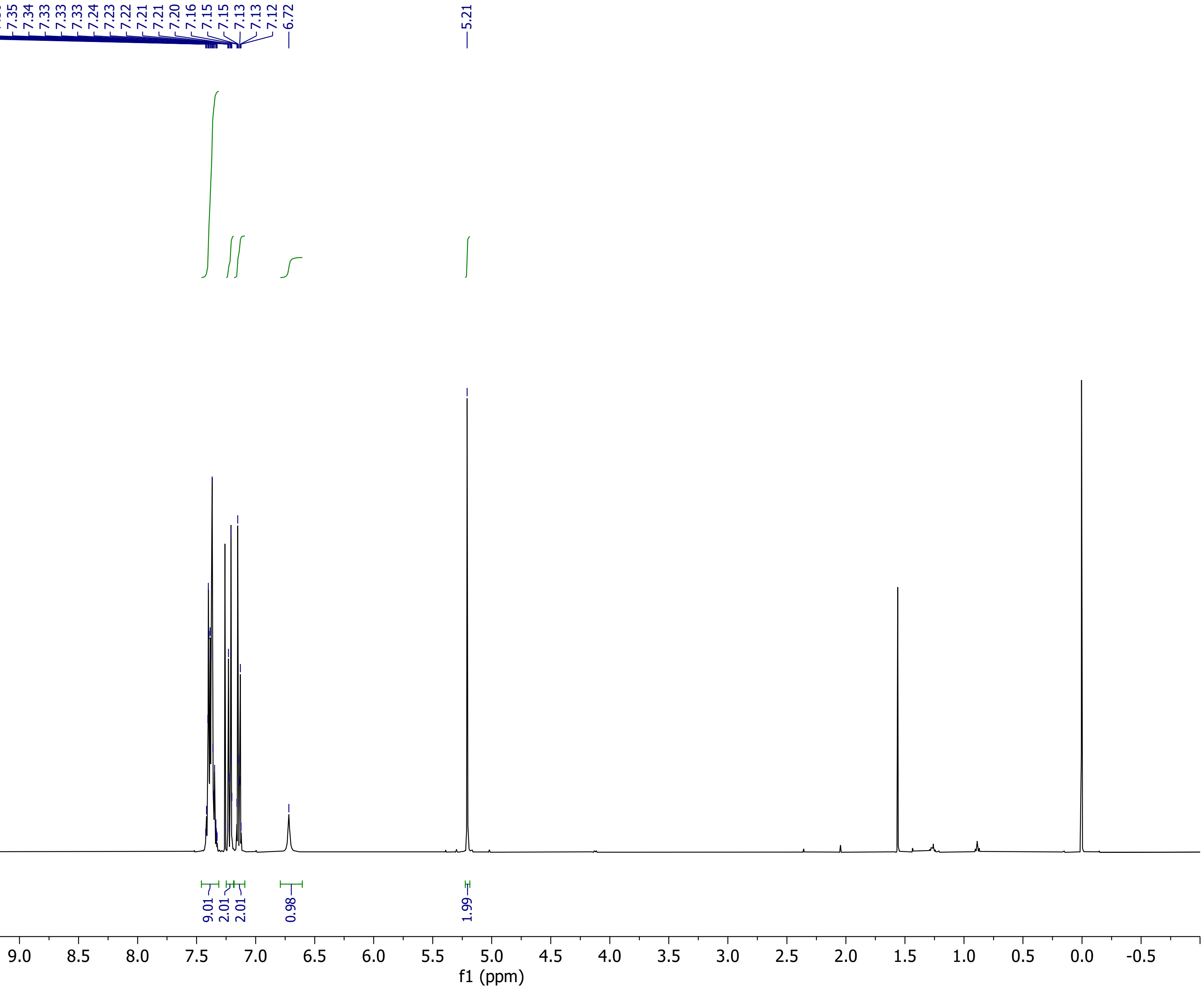

$^{13}C\{^1H\}$ NMR (101 MHz, $CDCl_3$)

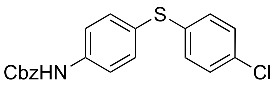

6f

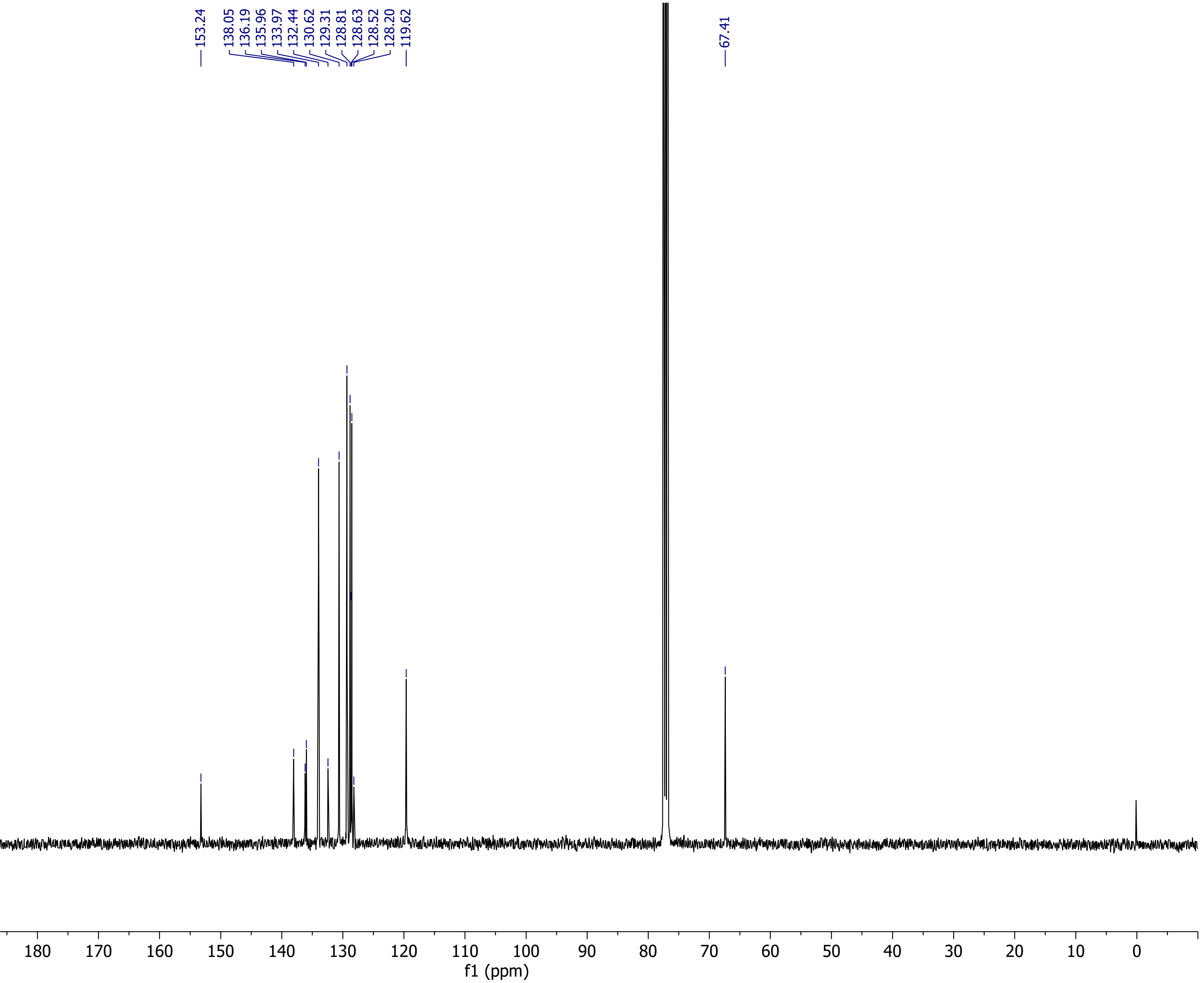

^{1}H NMR (400 MHz, CDCl$_3$)

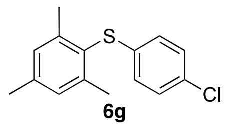

6g

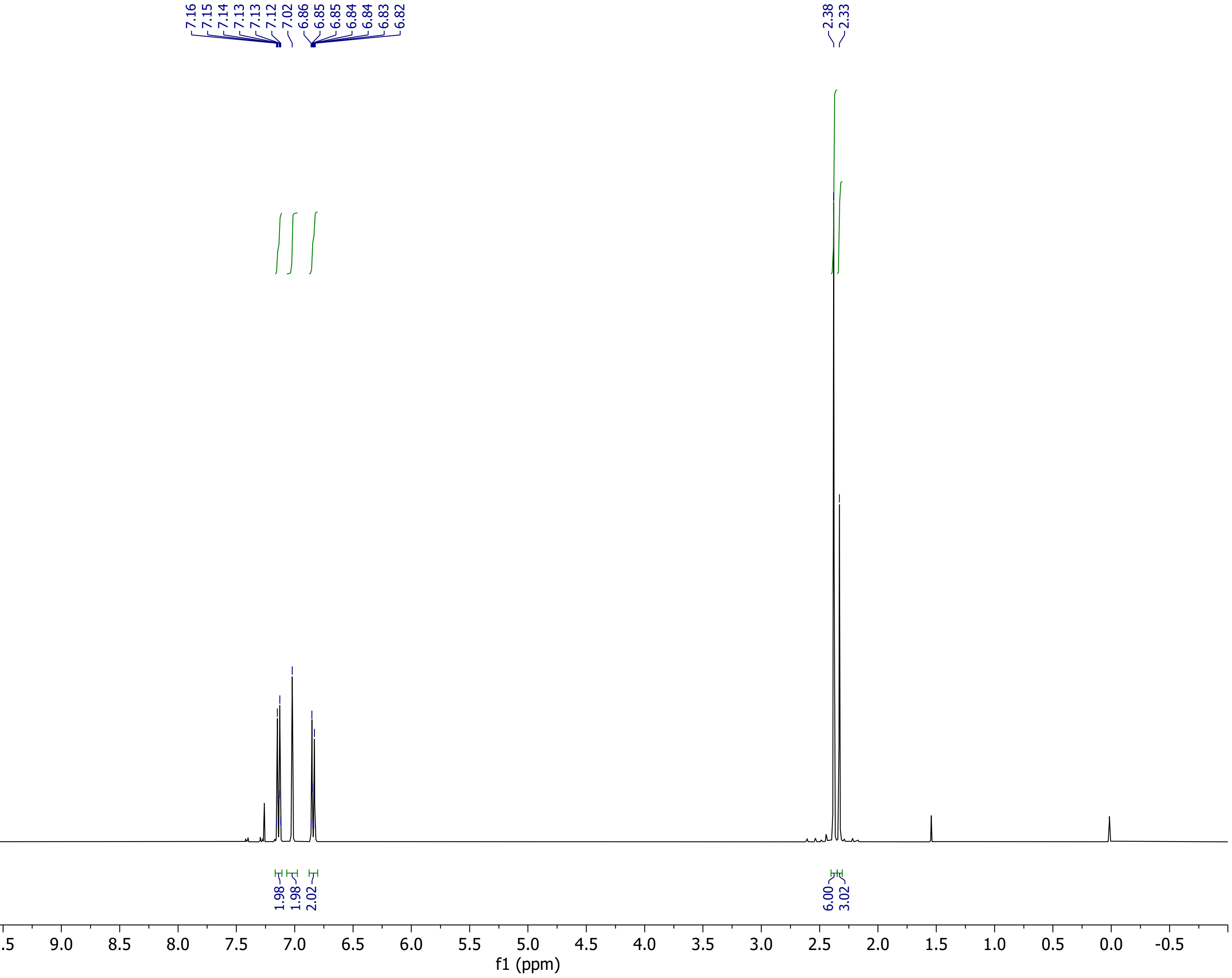

$^{13}C\{^{1}H\}$ NMR (101 MHz, $CDCl_3$)

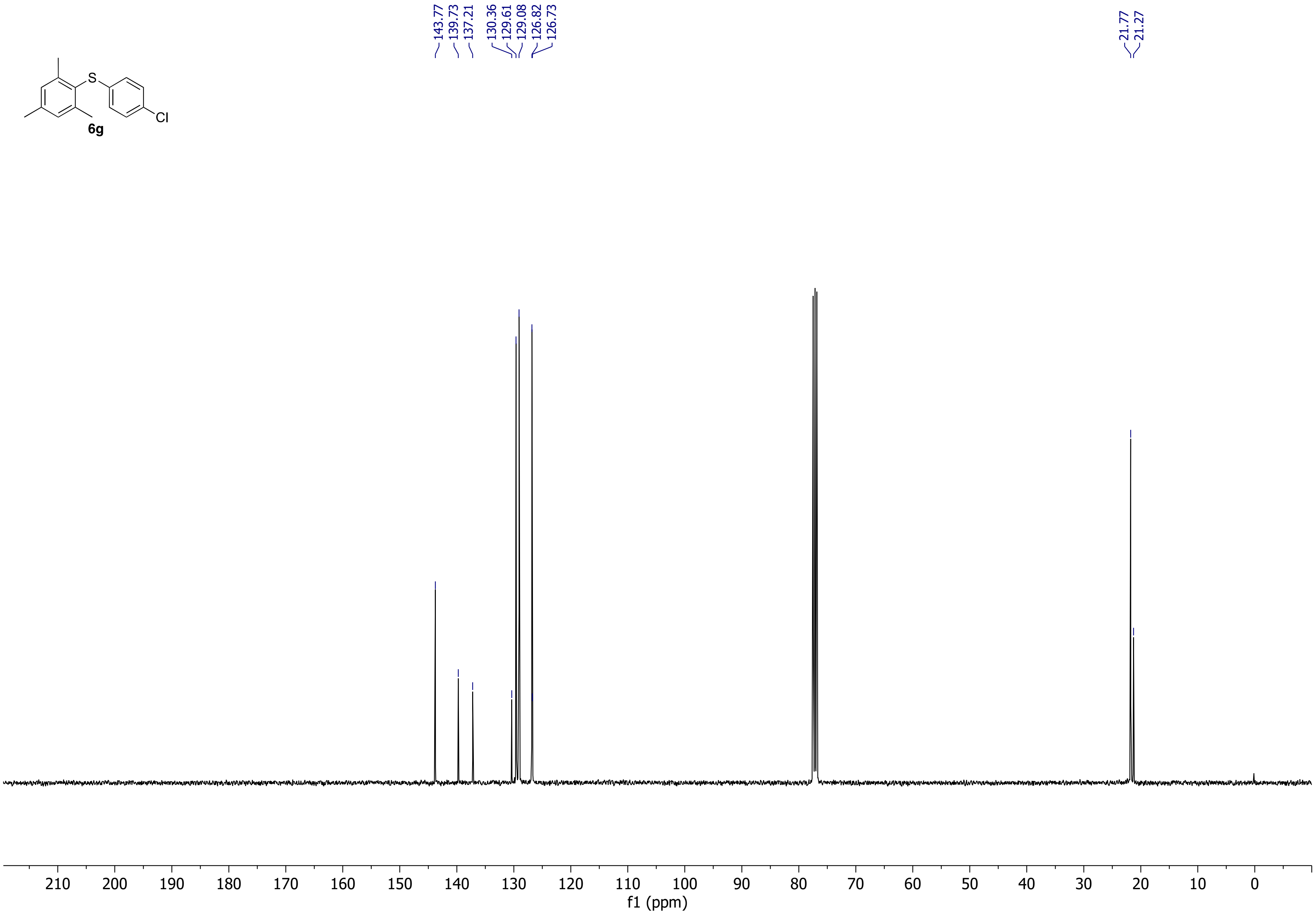

^{1}H NMR (400 MHz, $CDCl_3$)

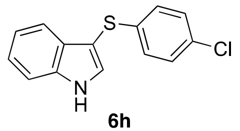

6h

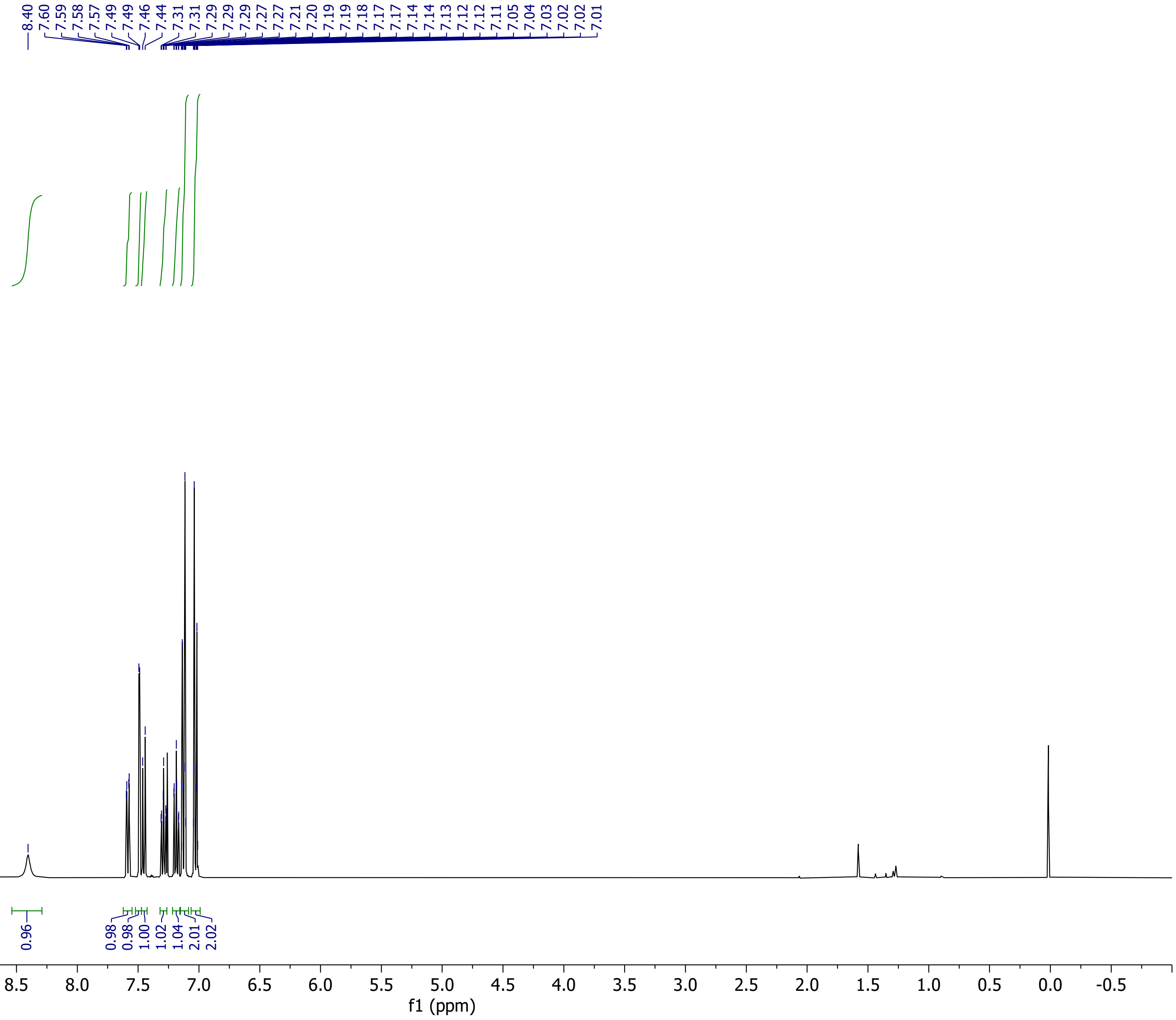

$^{13}C\{^{1}H\}$ NMR (101 MHz, $CDCl_3$)

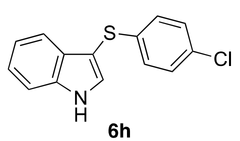

6h

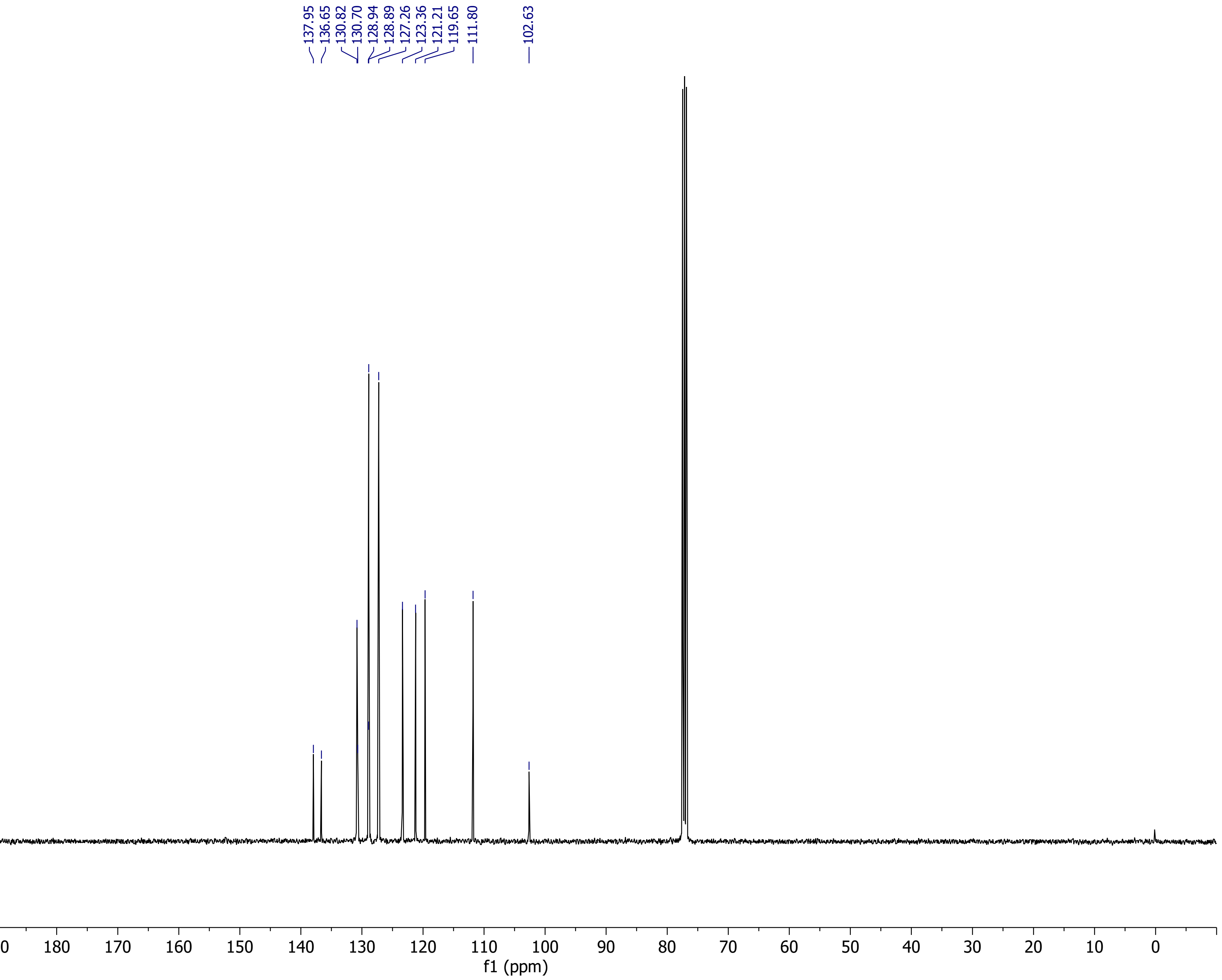

^{1}H NMR (400 MHz, $CDCl_3$)

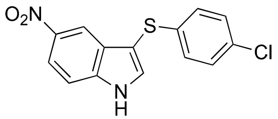

6i

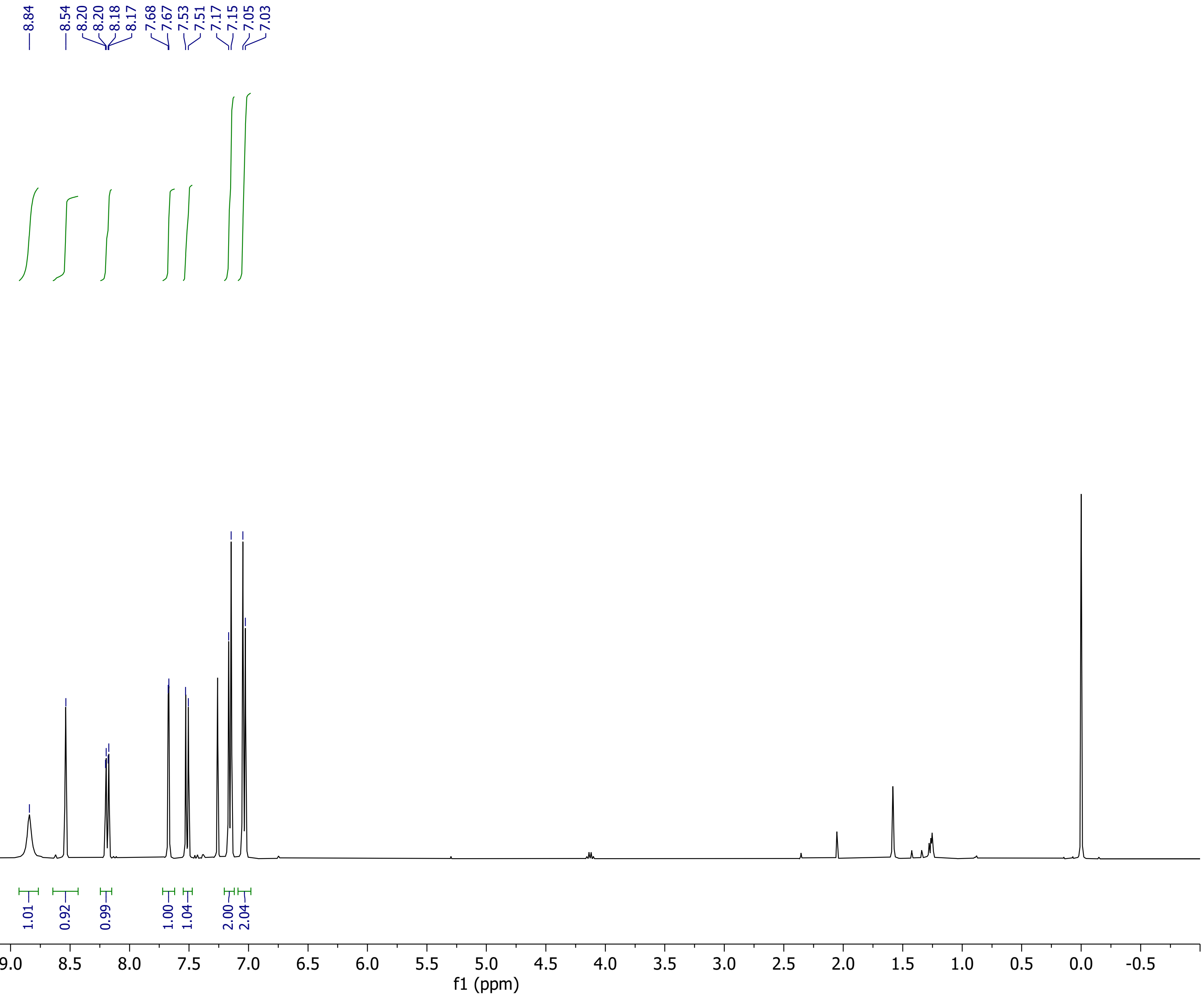

$^{13}C\{^{1}H\}$ NMR (101 MHz, $CDCl_3$)

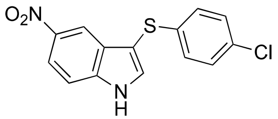

6i

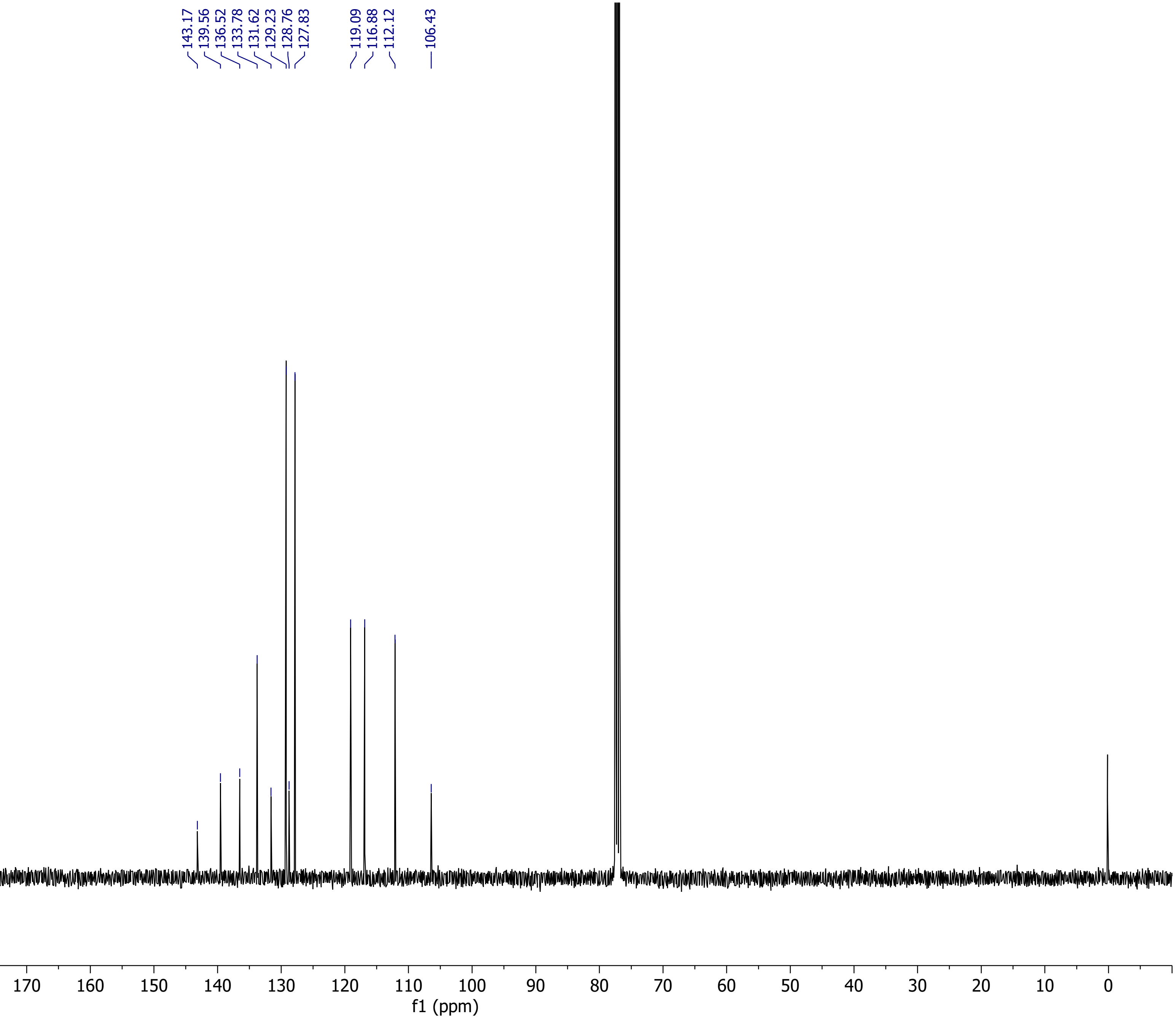

^{1}H NMR (400 MHz, $CDCl_3$)

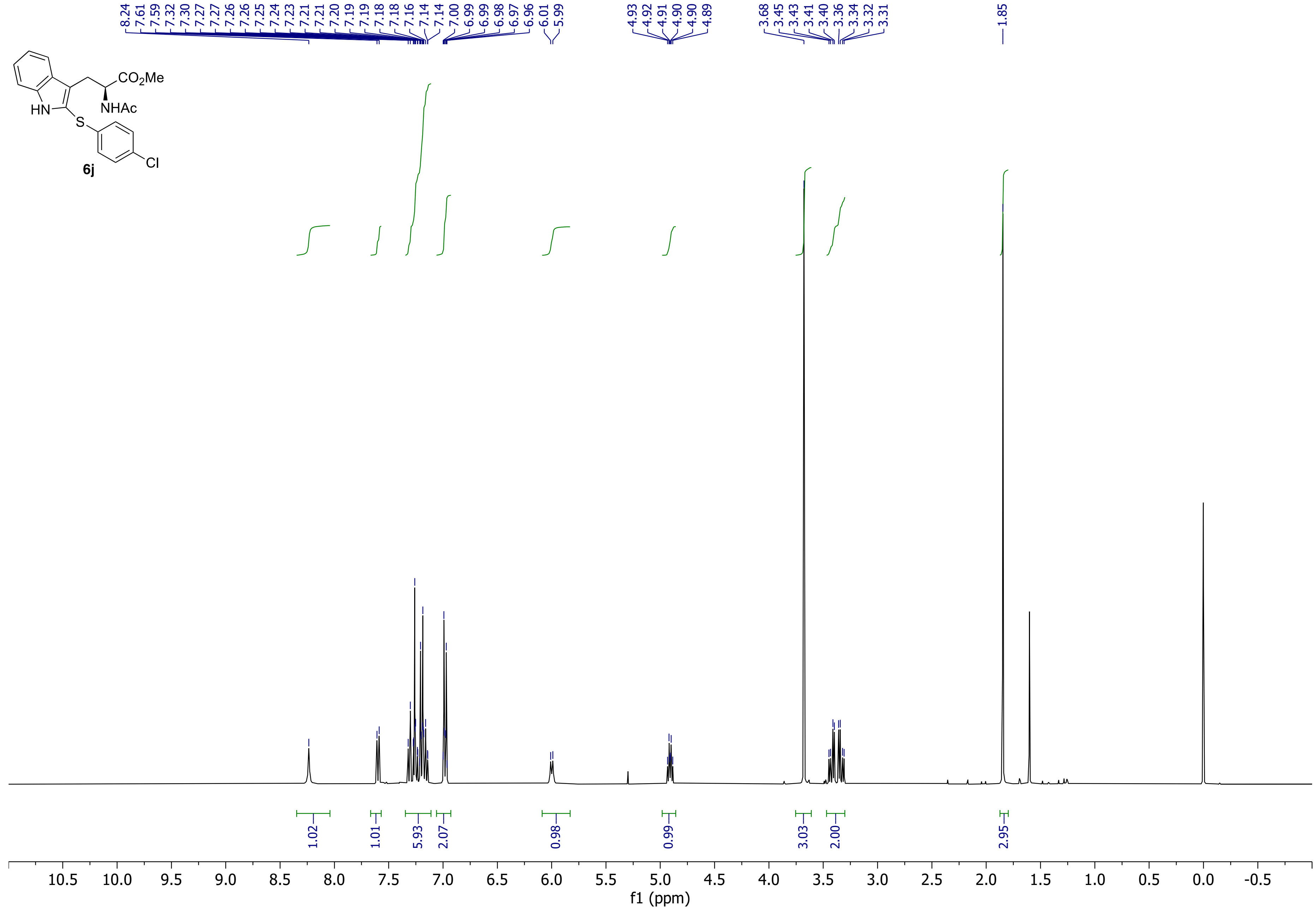

$^{13}C\{^1H\}$ NMR (101 MHz, CDCl$_3$)

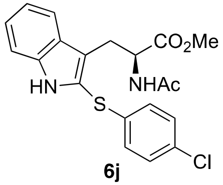

6j

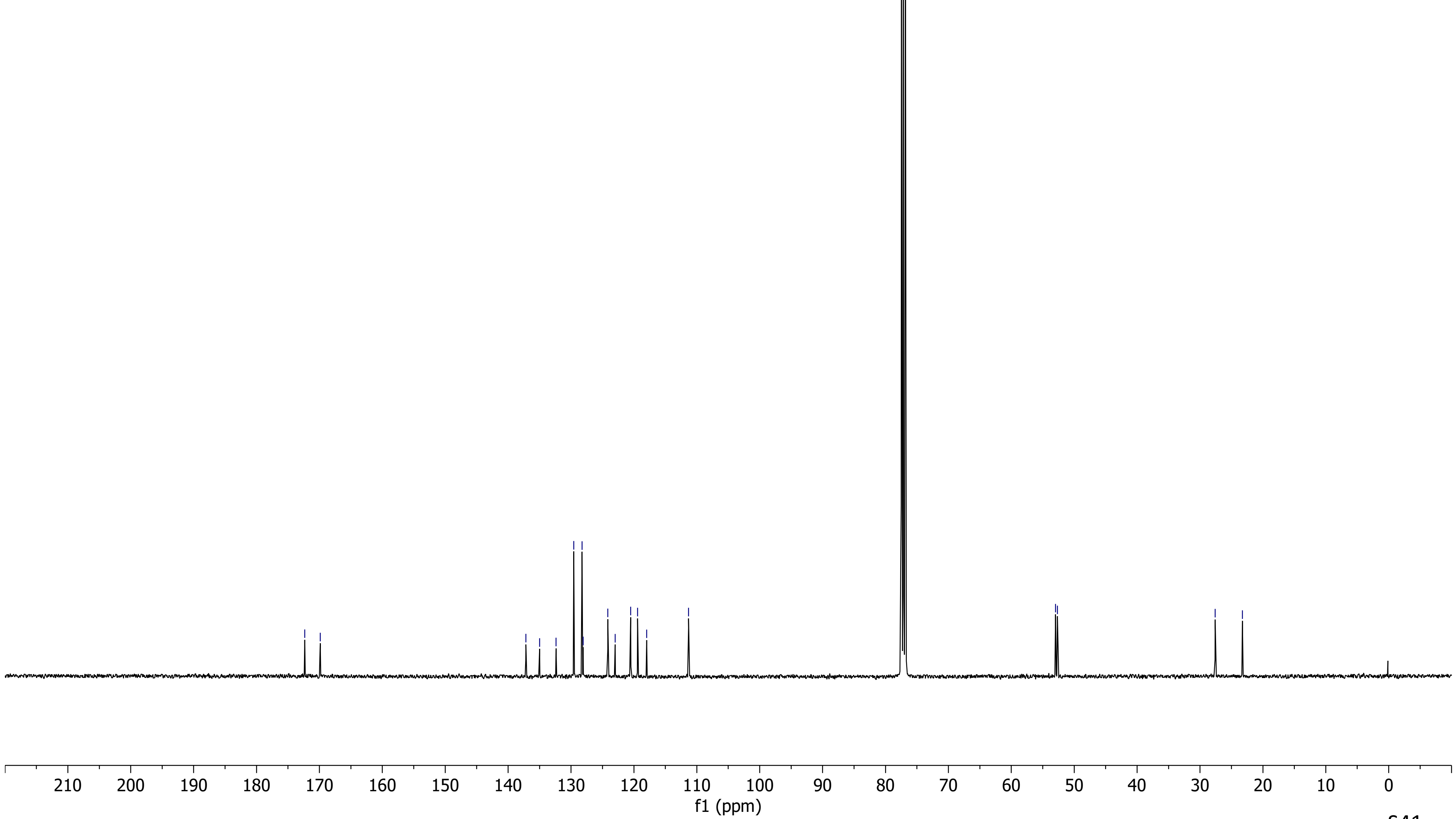

^{1}H NMR (400 MHz, $CDCl_3$)

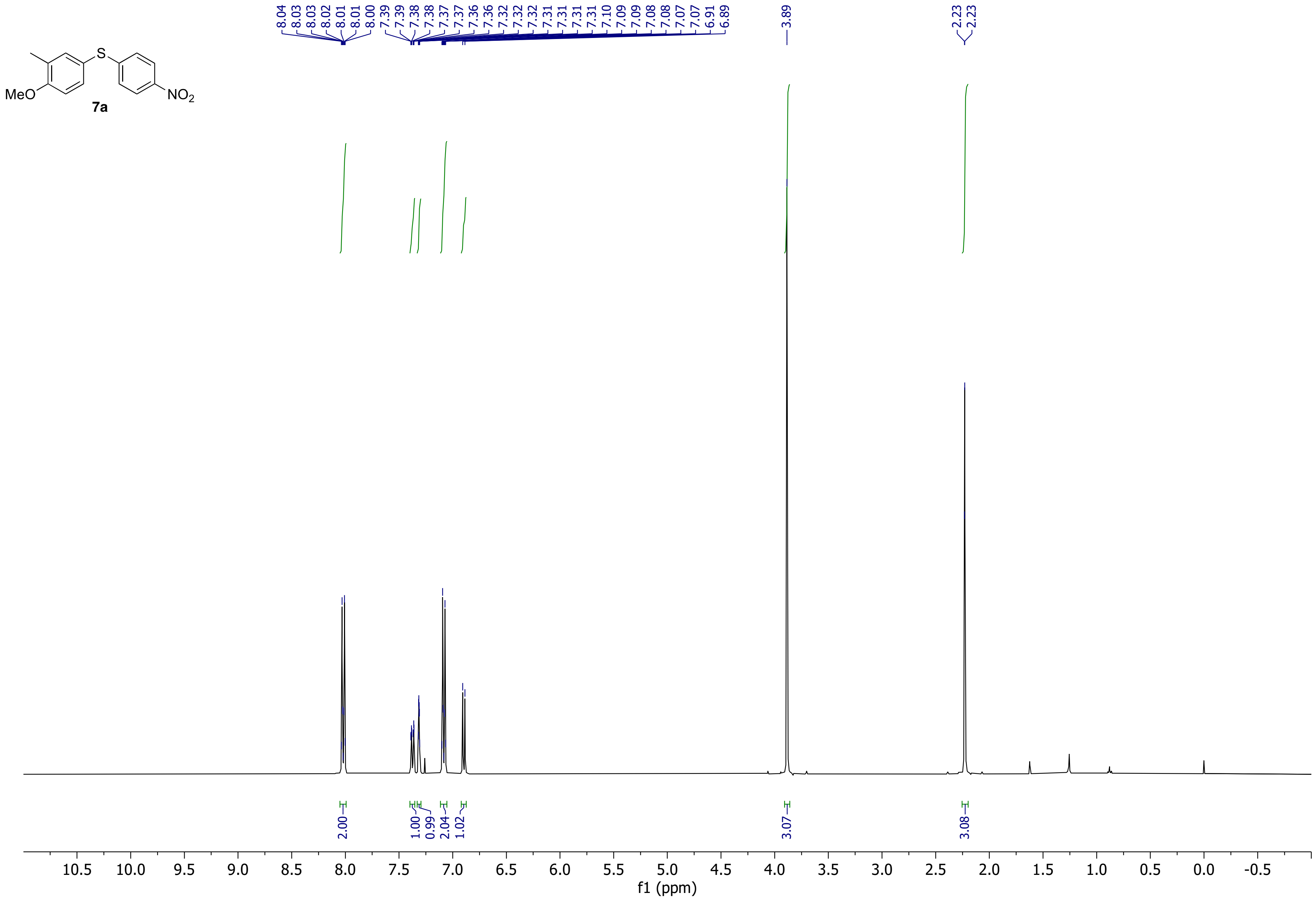

$^{13}C\{^{1}H\}$ NMR (101 MHz, $CDCl_3$)

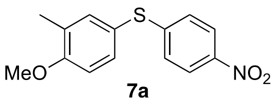

7a

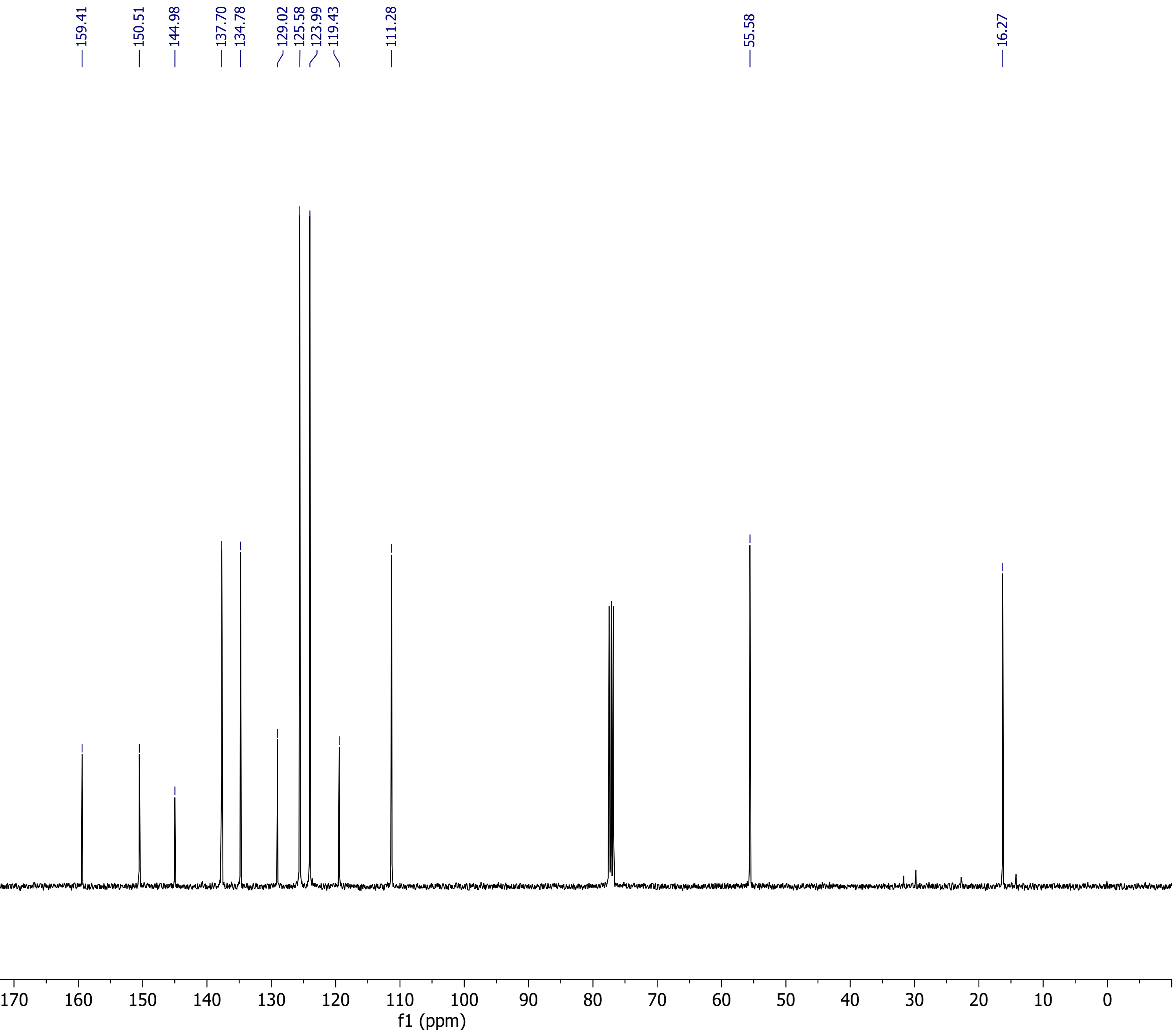

^{1}H NMR (400 MHz, CDCl$_3$)

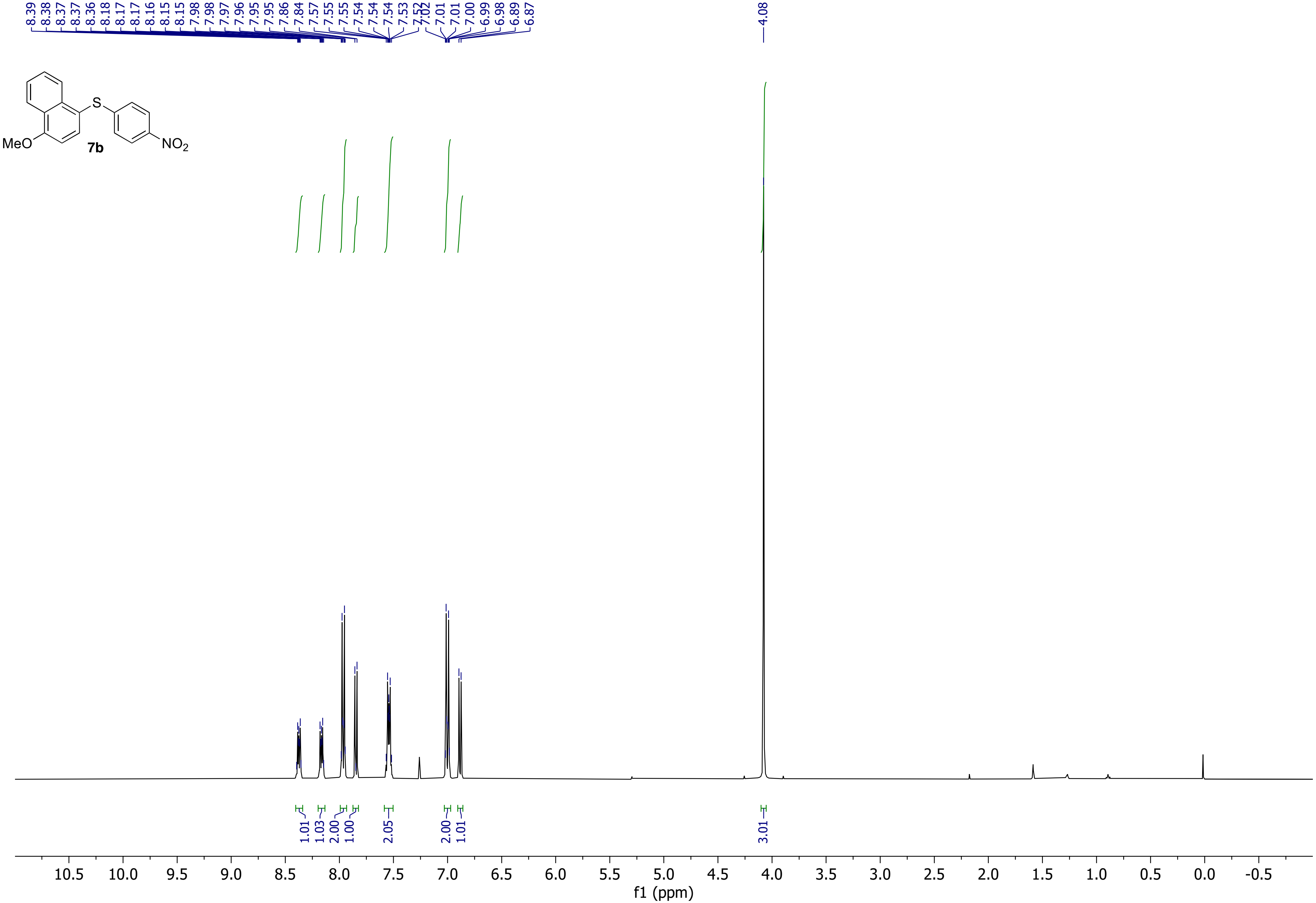

$^{13}C\{^1H\}$ NMR (101 MHz, $CDCl_3$)

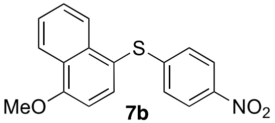

7b

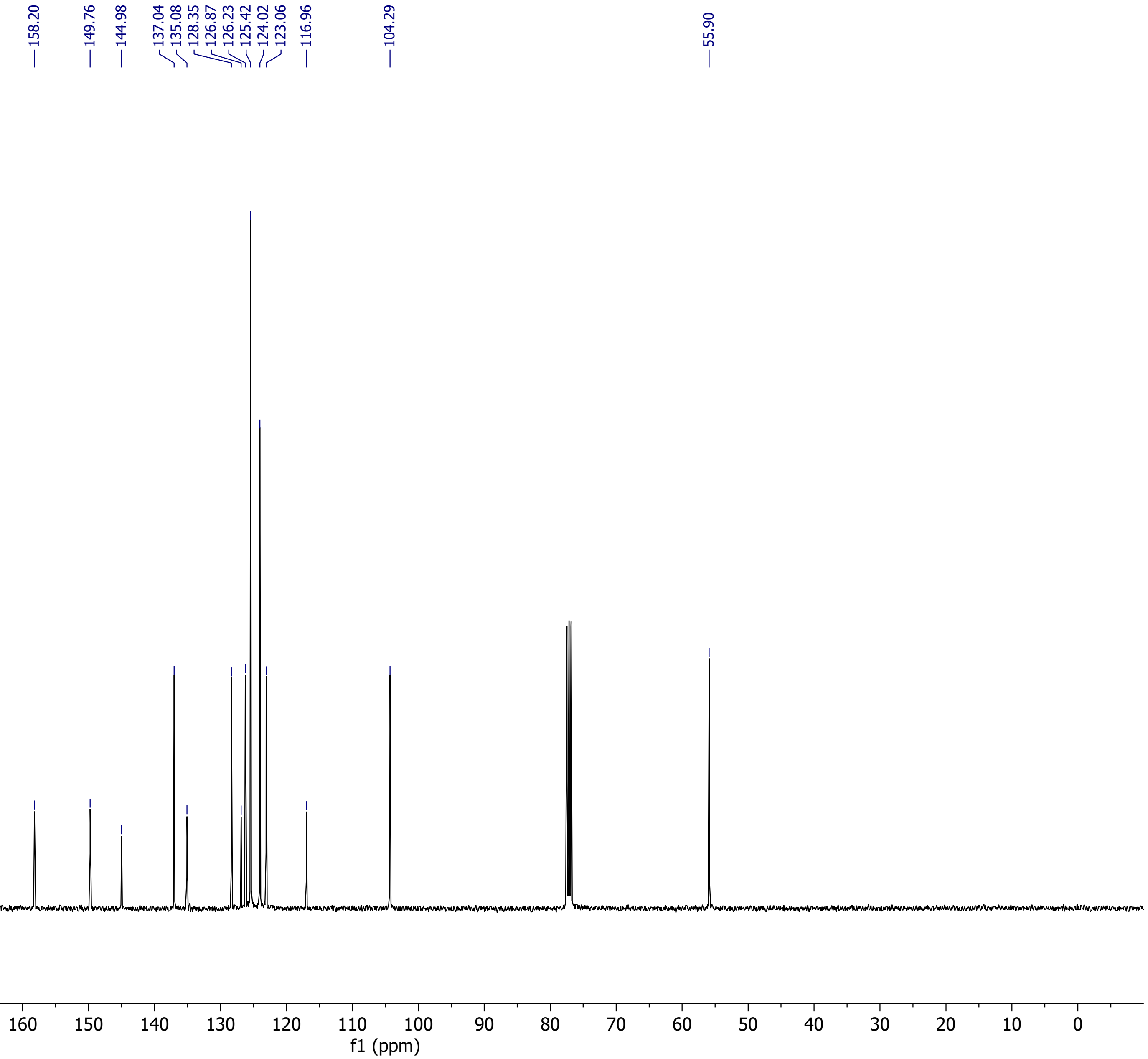

^{1}H NMR (400 MHz, CDCl$_3$)

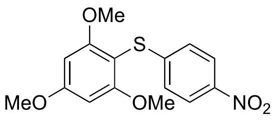

7c

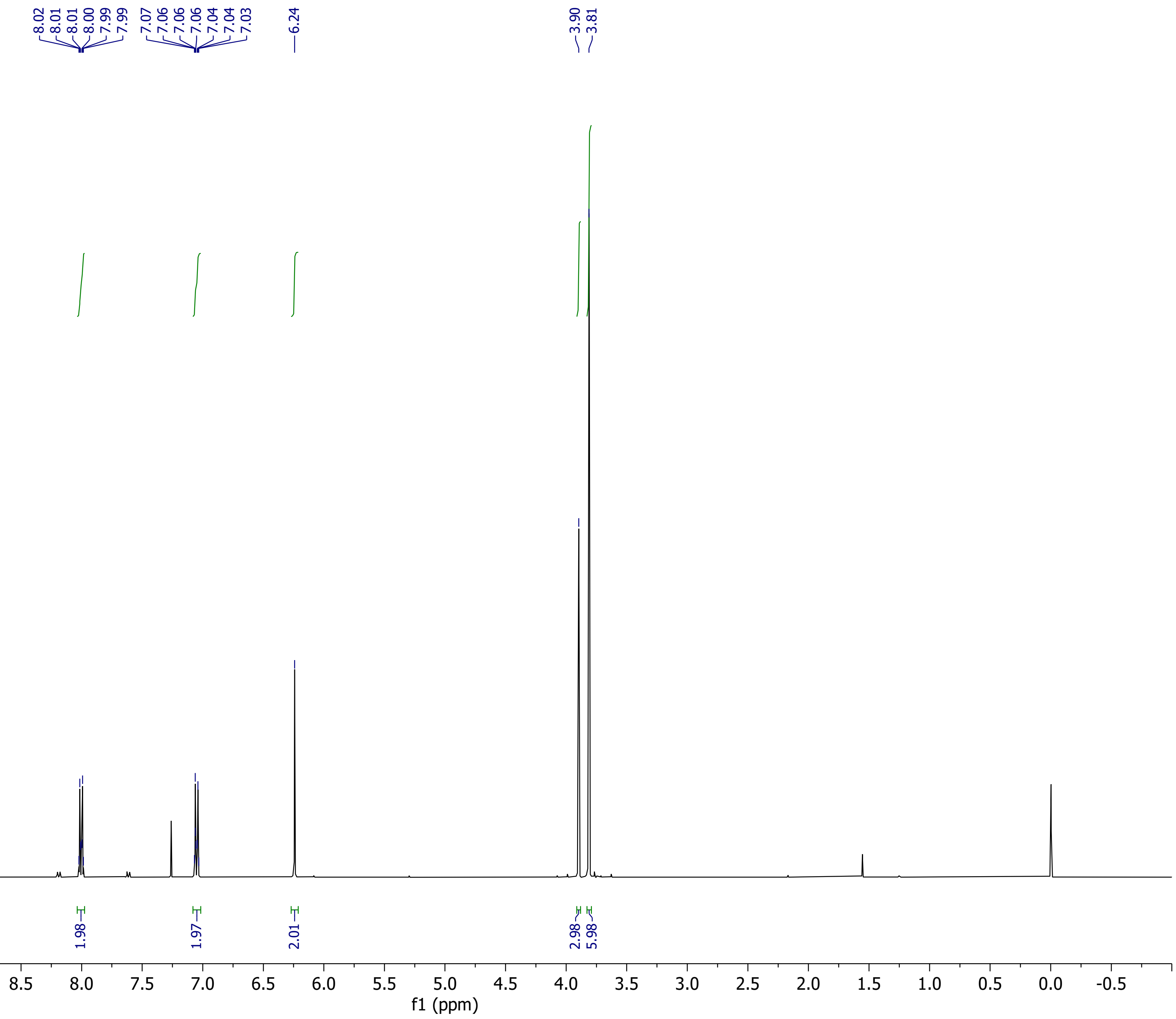

$^{13}C\{^{1}H\}$ NMR (101 MHz, $CDCl_3$)

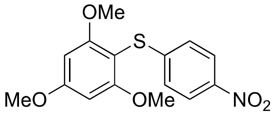

7c

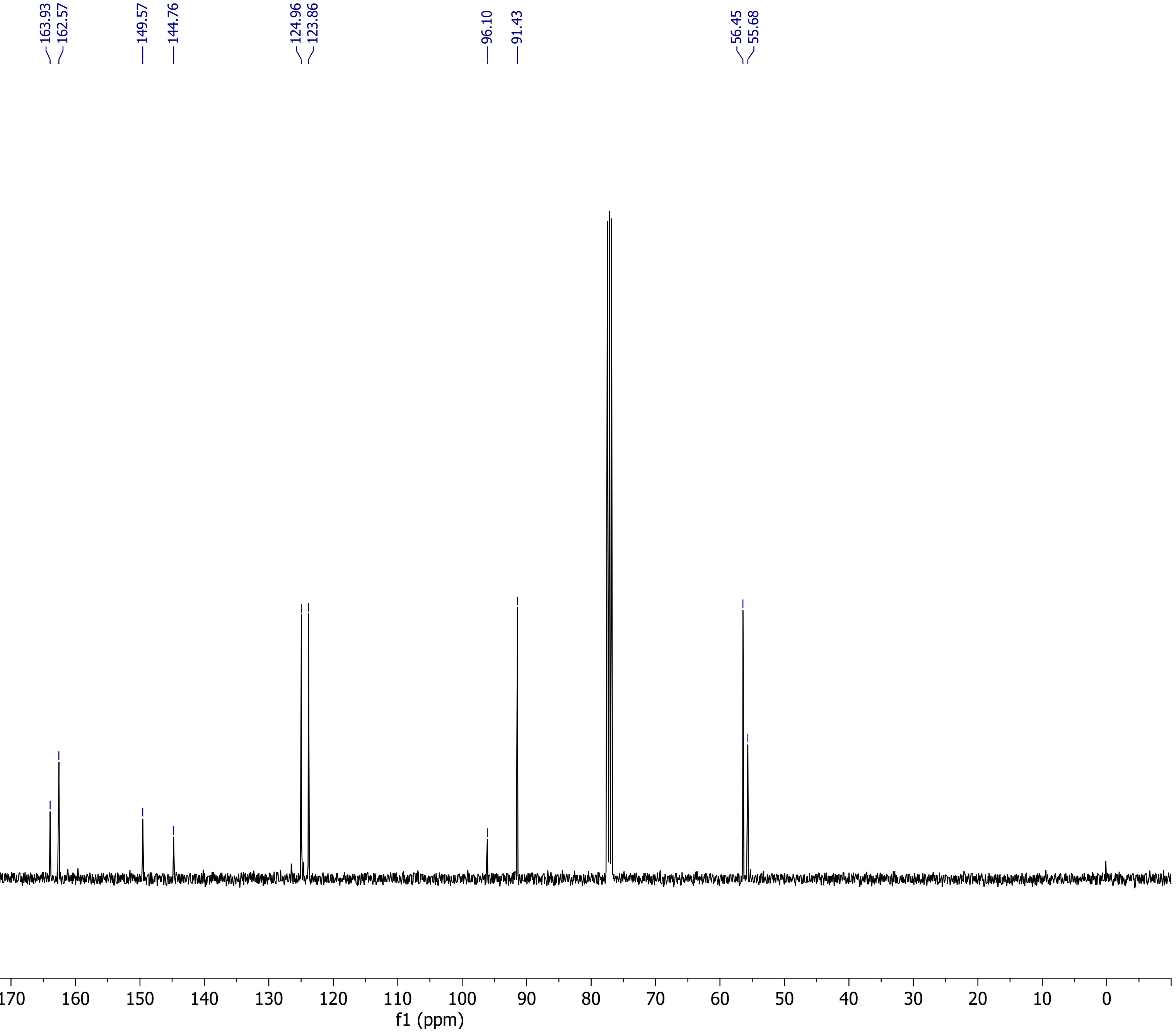

^{1}H NMR (400 MHz, CDCl$_3$)

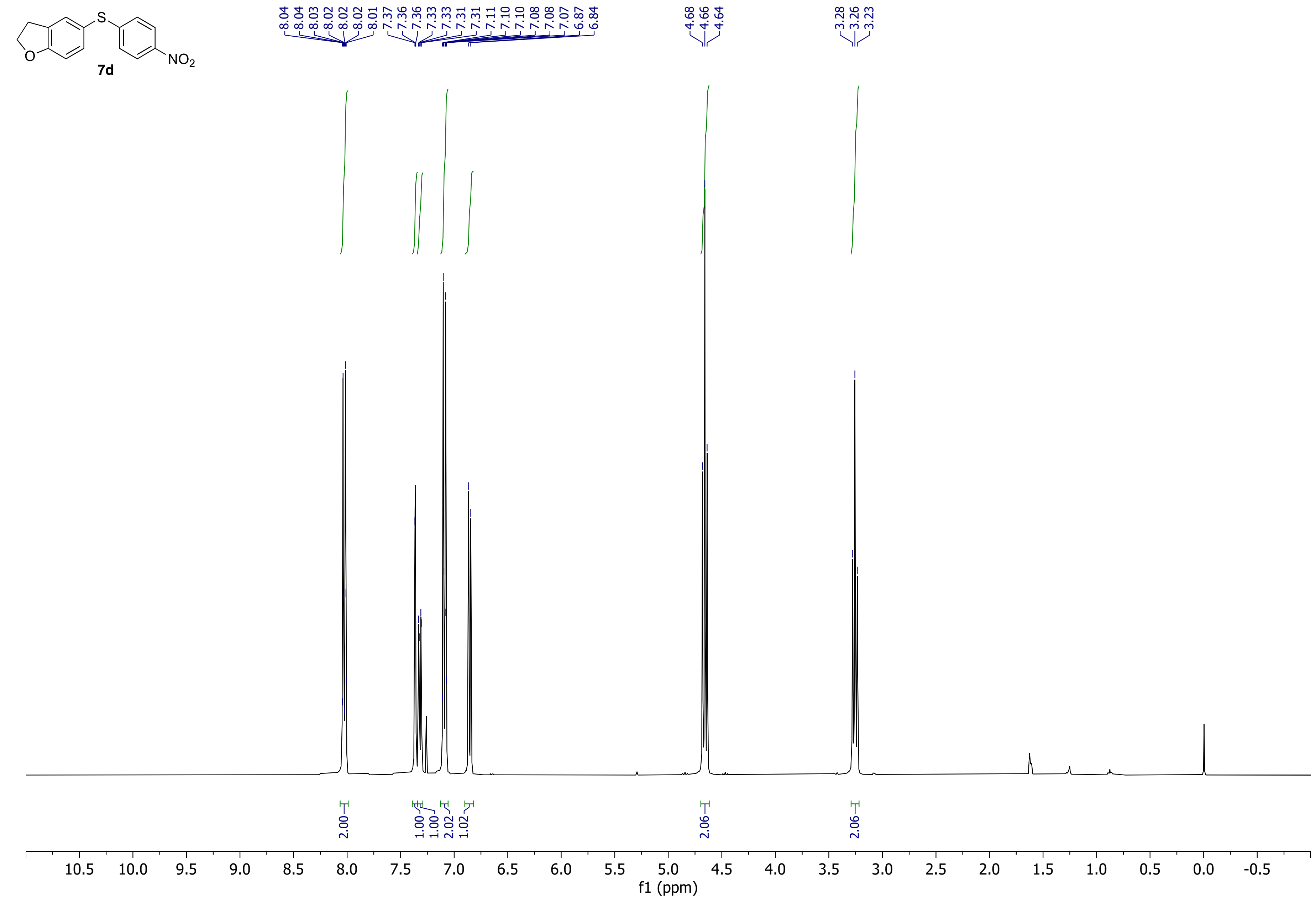

$^{13}C\{^{1}H\}$ NMR (101 MHz, $CDCl_3$)

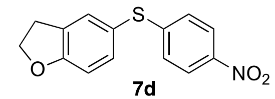

7d

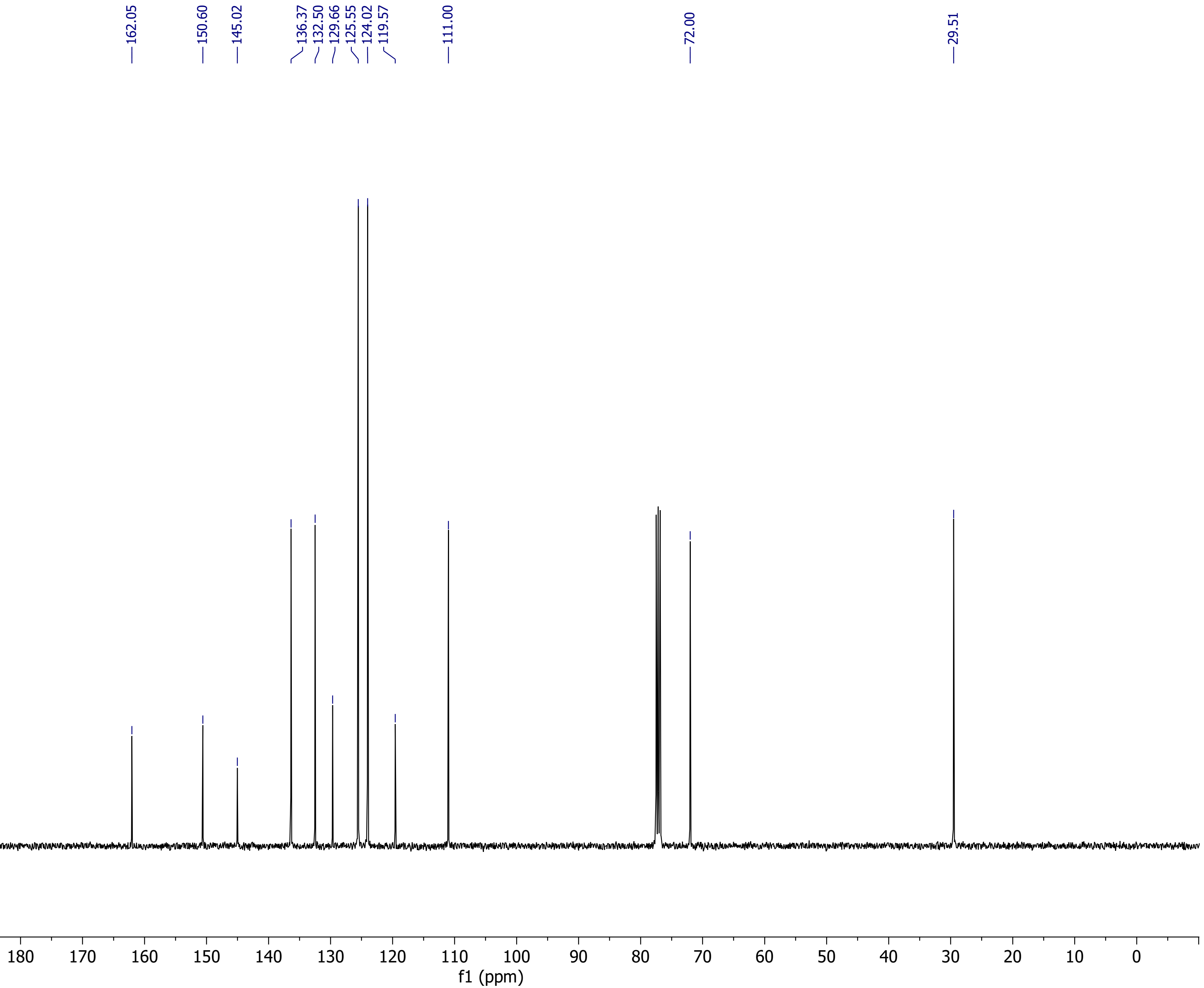

^{1}H NMR (400 MHz, DMSO-d_6)

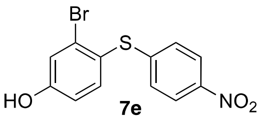

7e

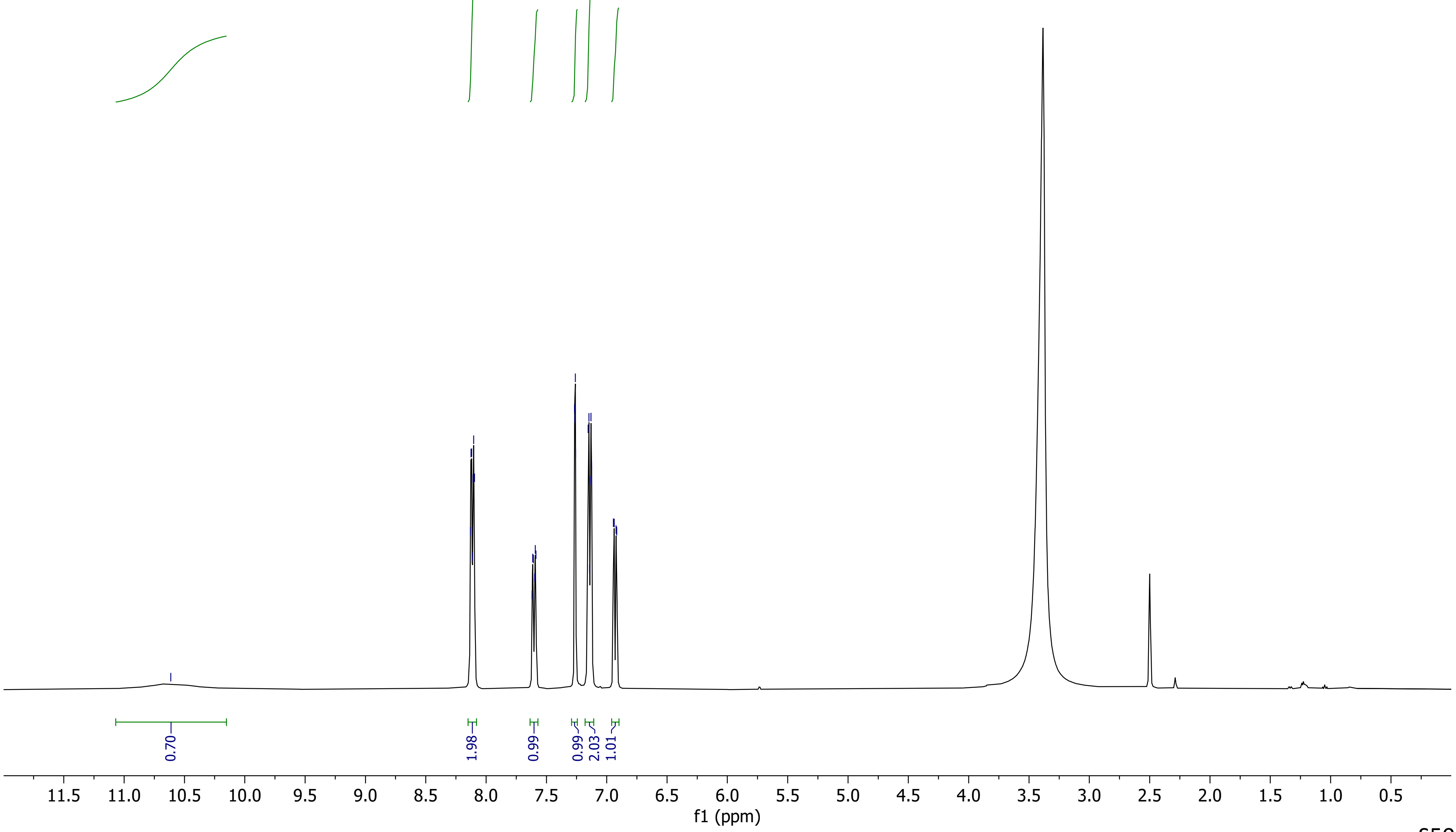

$^{13}C\{^{1}H\}$ NMR (101 MHz, DMSO-d_6)

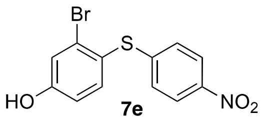

7e

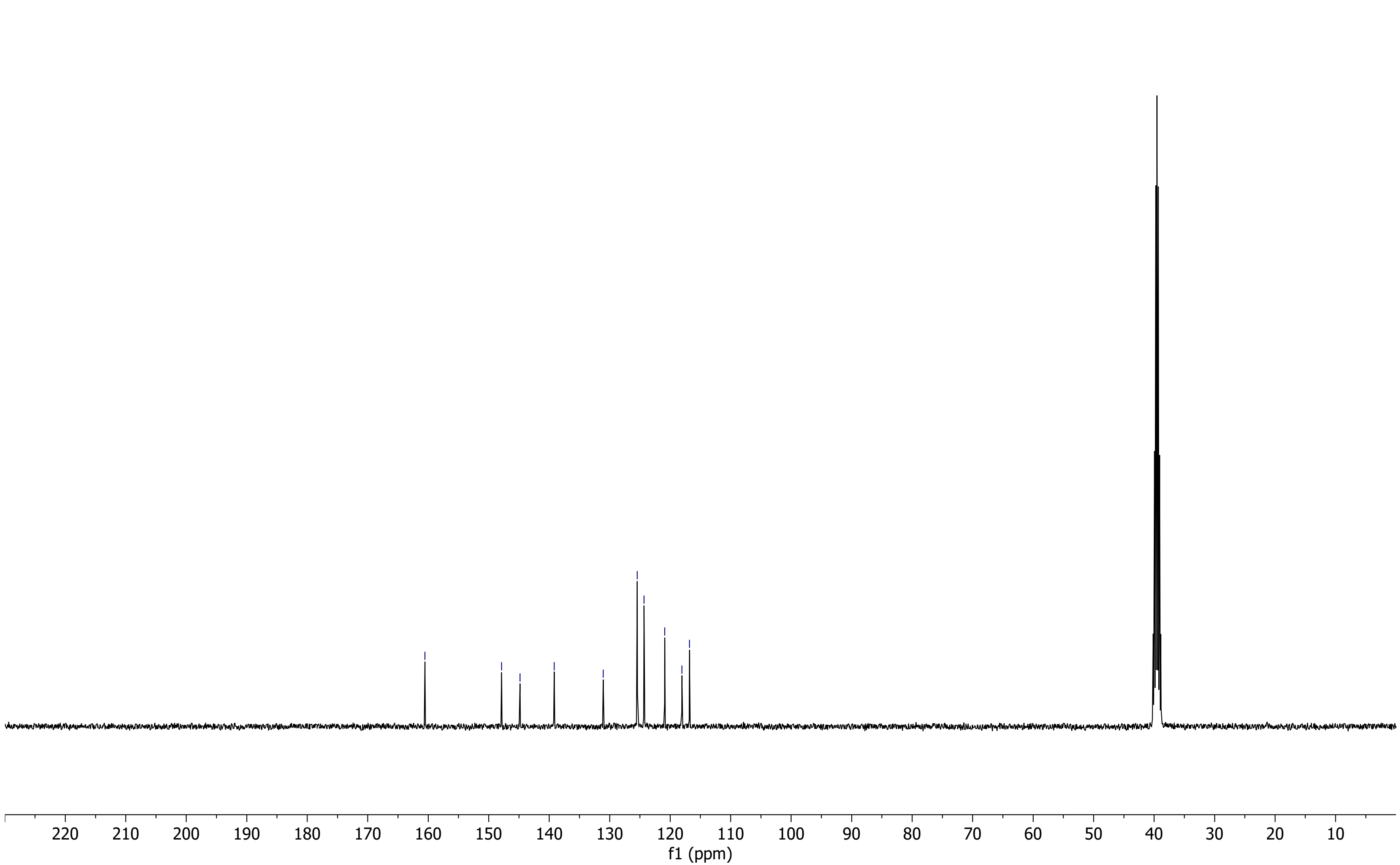

^{1}H NMR (400 MHz, $CDCl_3$)

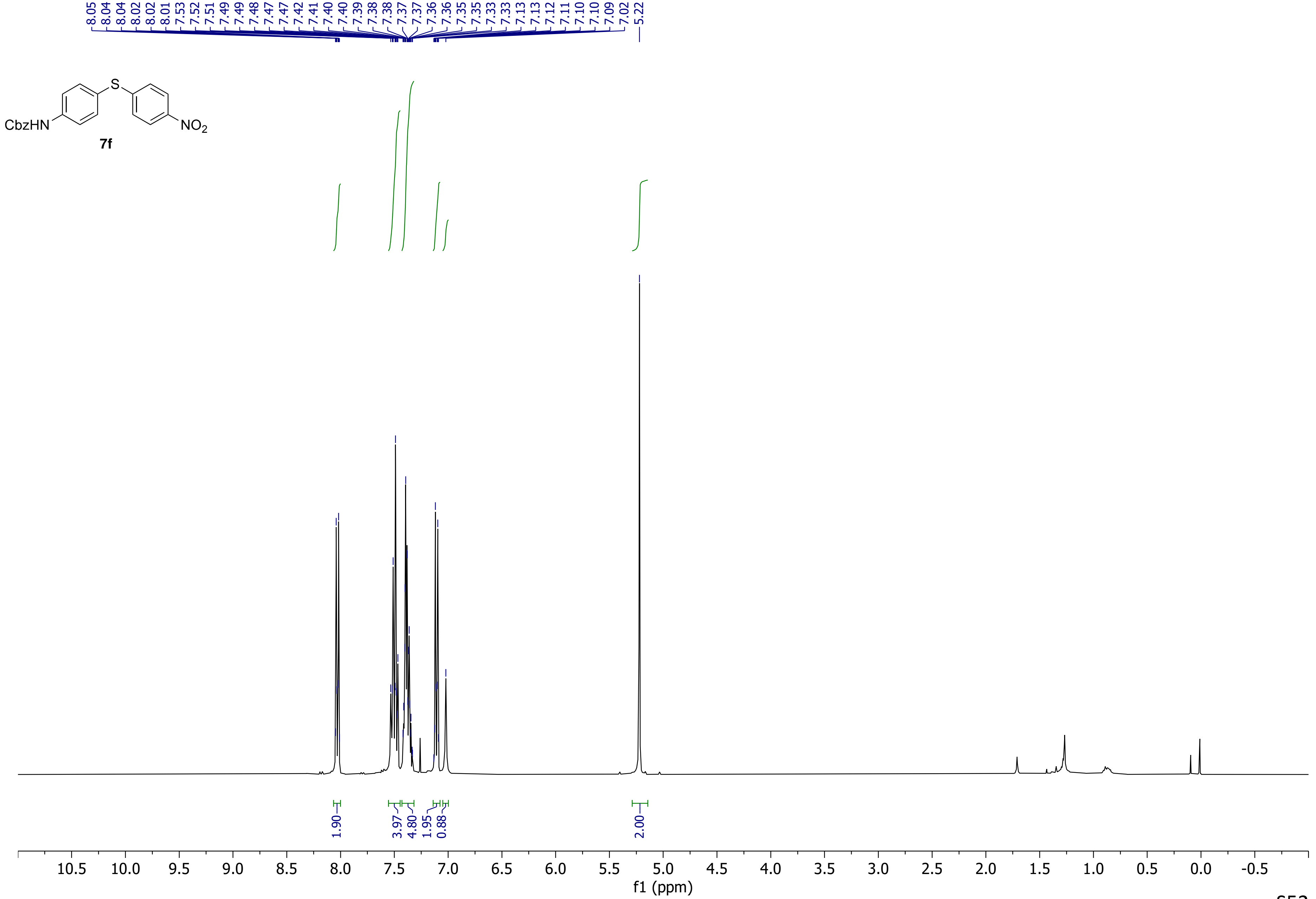

$^{13}C\{^{1}H\}$ NMR (101 MHz, $CDCl_3$)

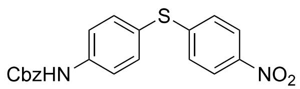

7f

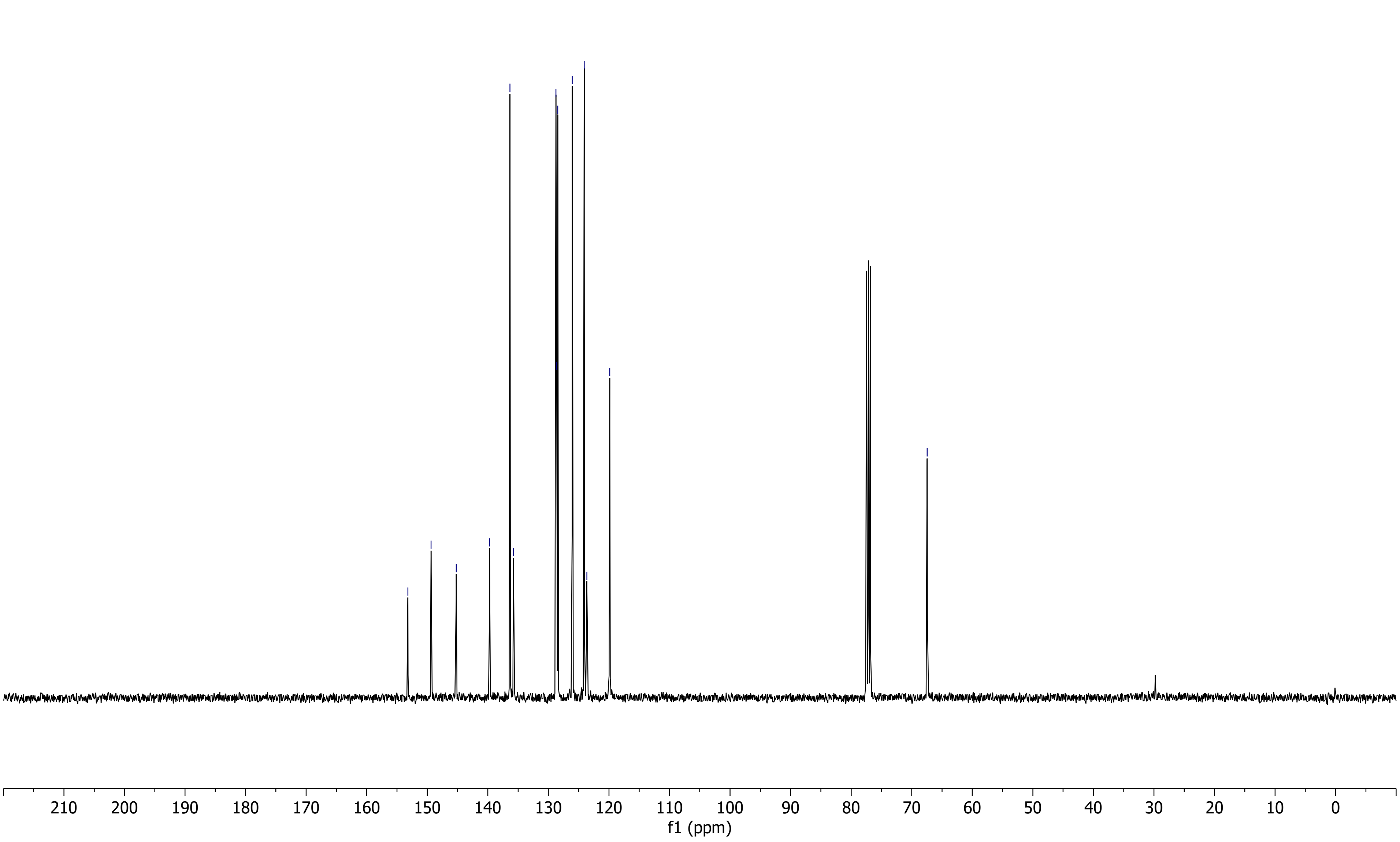

^{1}H NMR (400 MHz, $CDCl_3$)

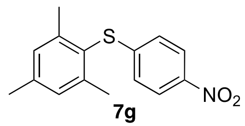

7g

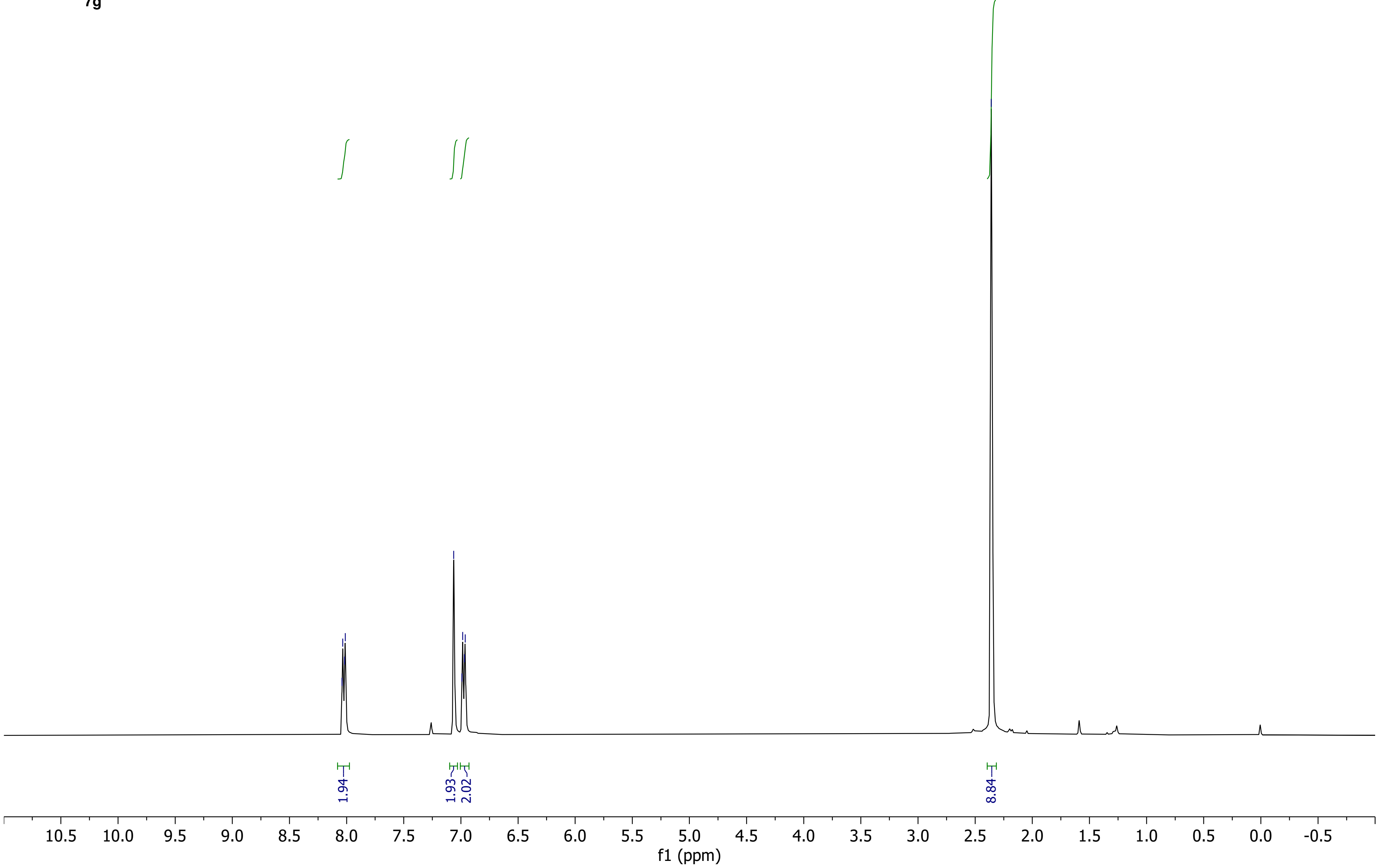

$^{13}C\{^1H\}$ NMR (101 MHz, $CDCl_3$)

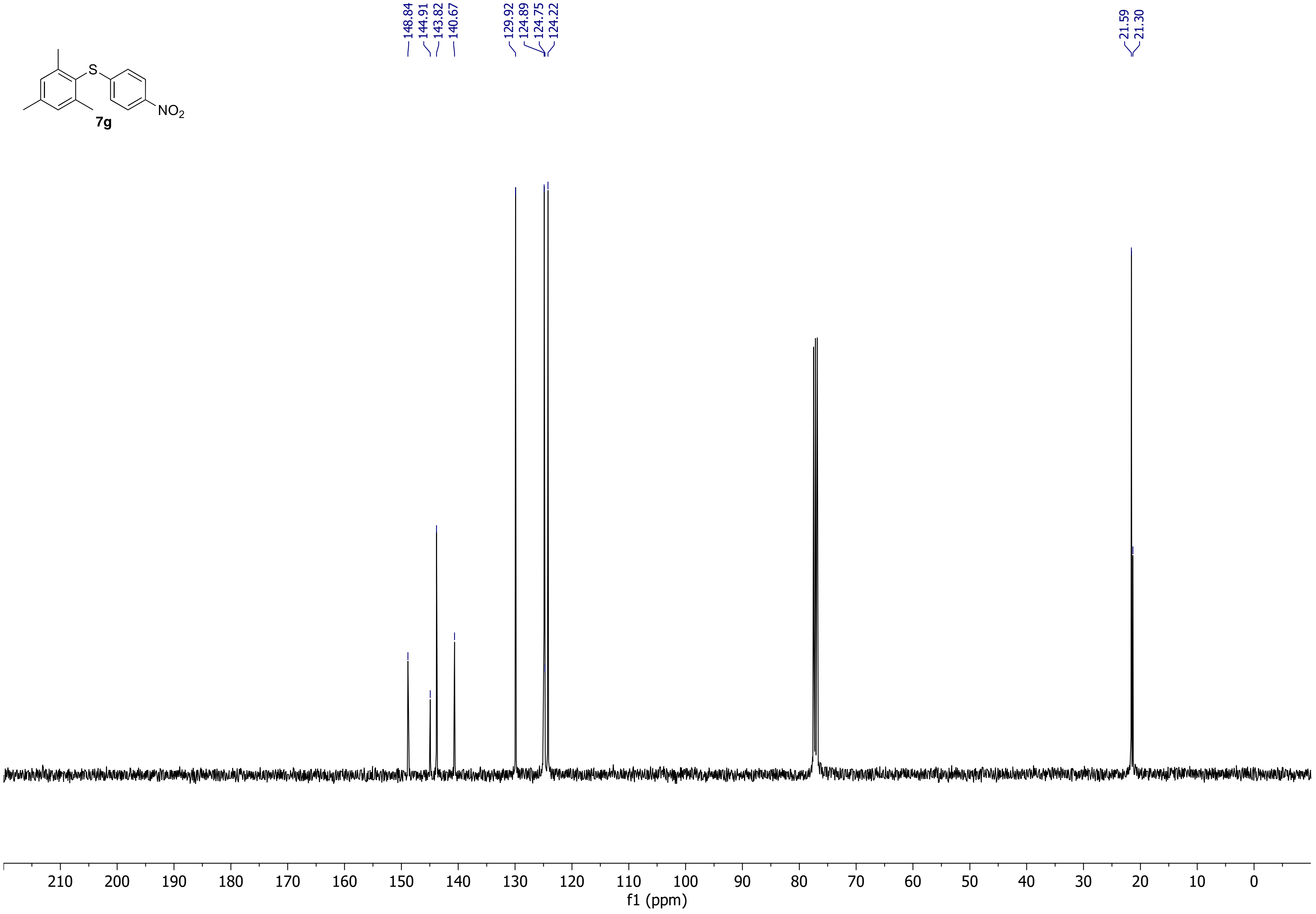

^{1}H NMR (400 MHz, CDCl$_3$)

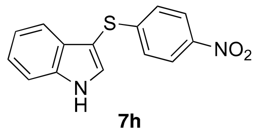

7h

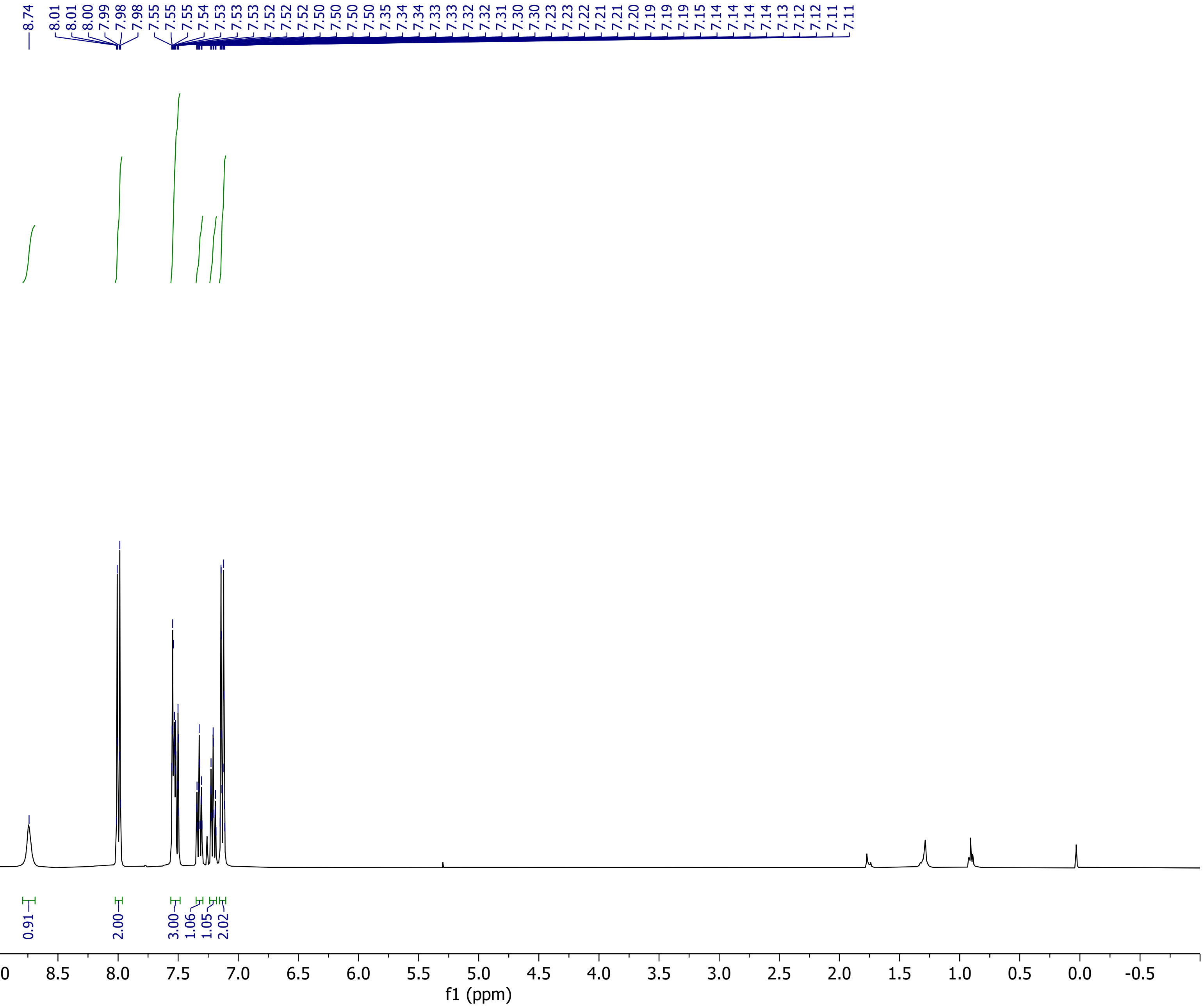

$^{13}C\{^1H\}$ NMR (101 MHz, $CDCl_3$)

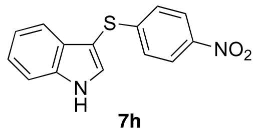

7h

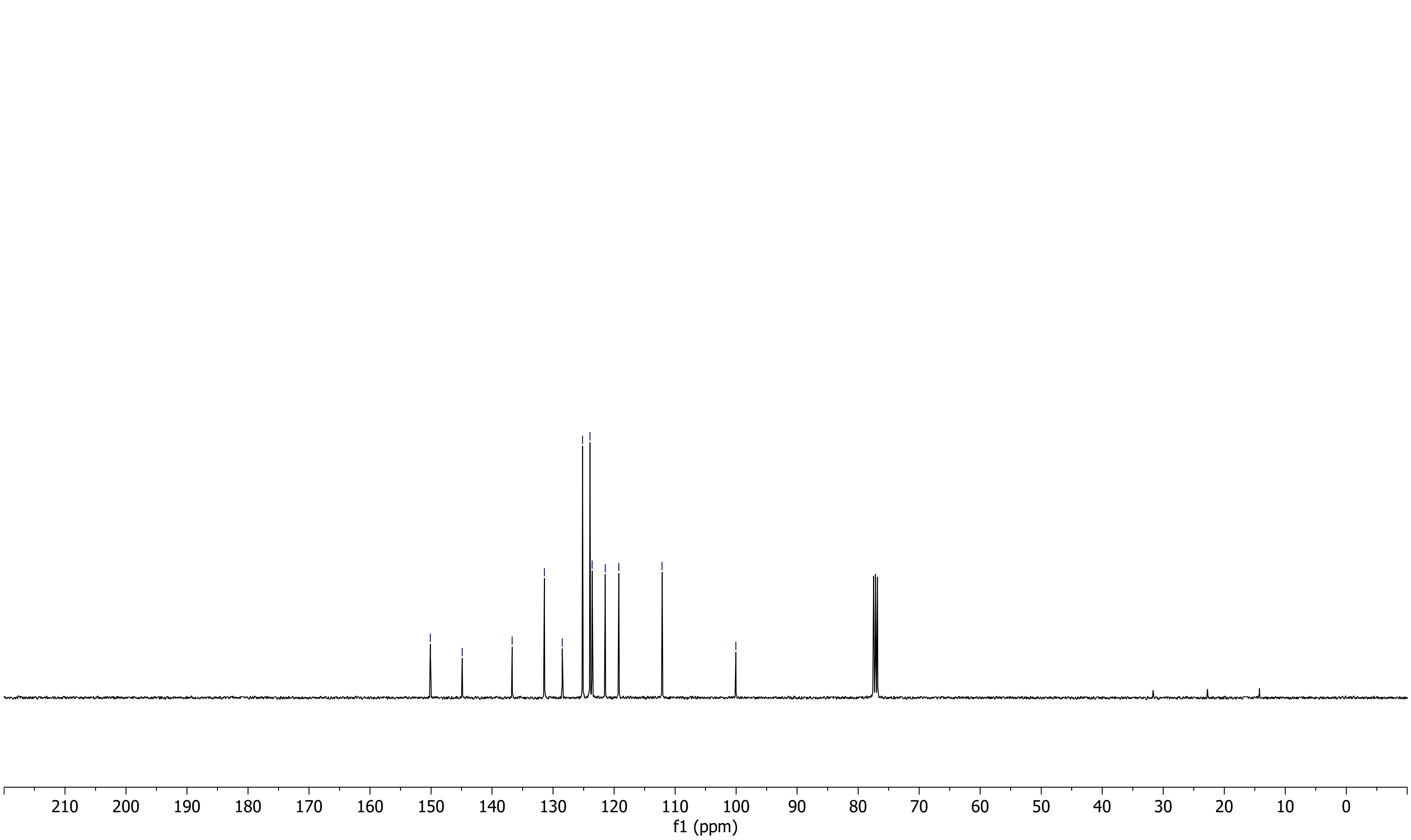

^{1}H NMR (400 MHz, DMSO-d_6)

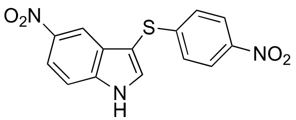

7i

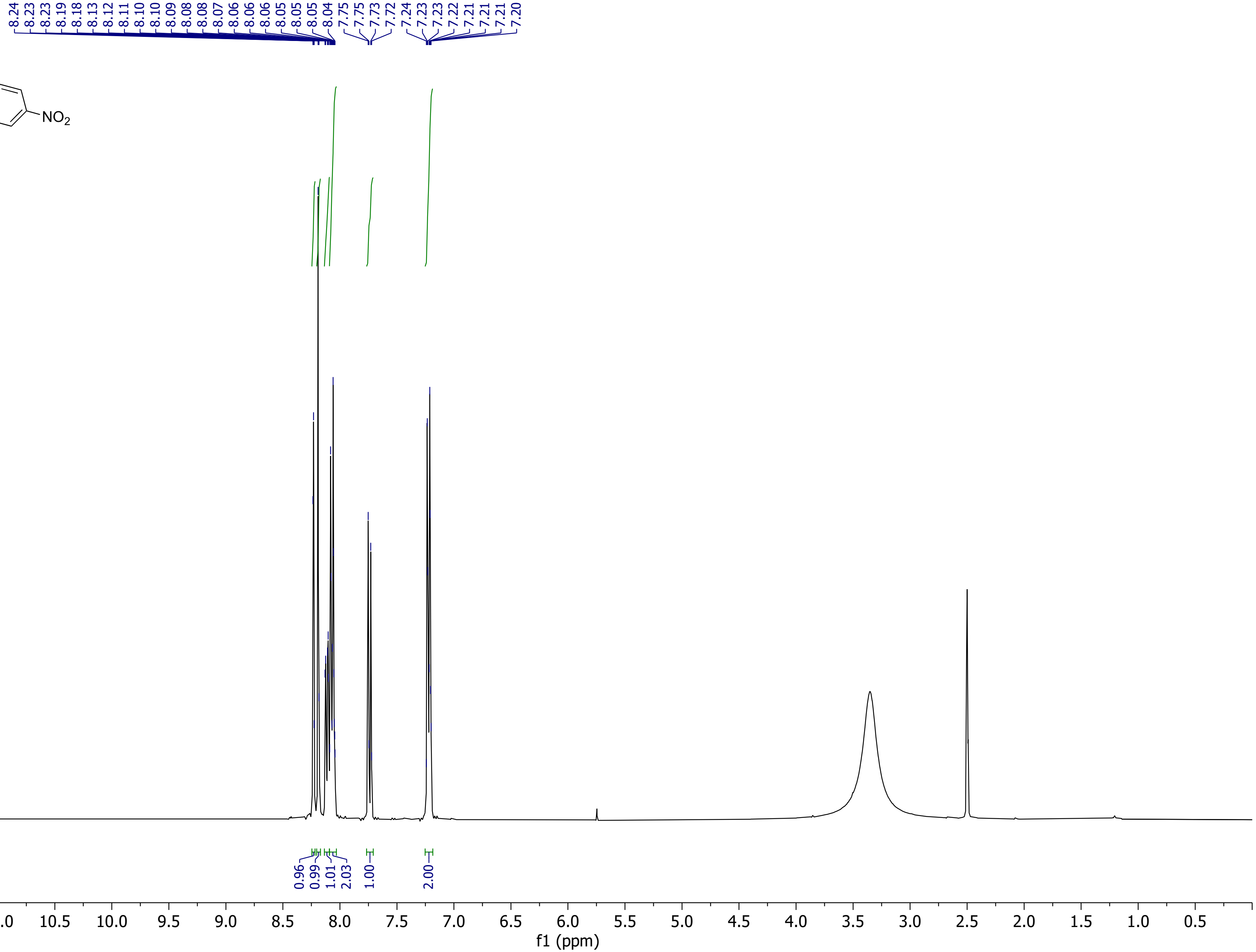

$^{13}C\{^1H\}$ NMR (101 MHz, DMSO-d_6)

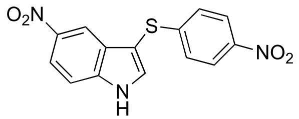

7i

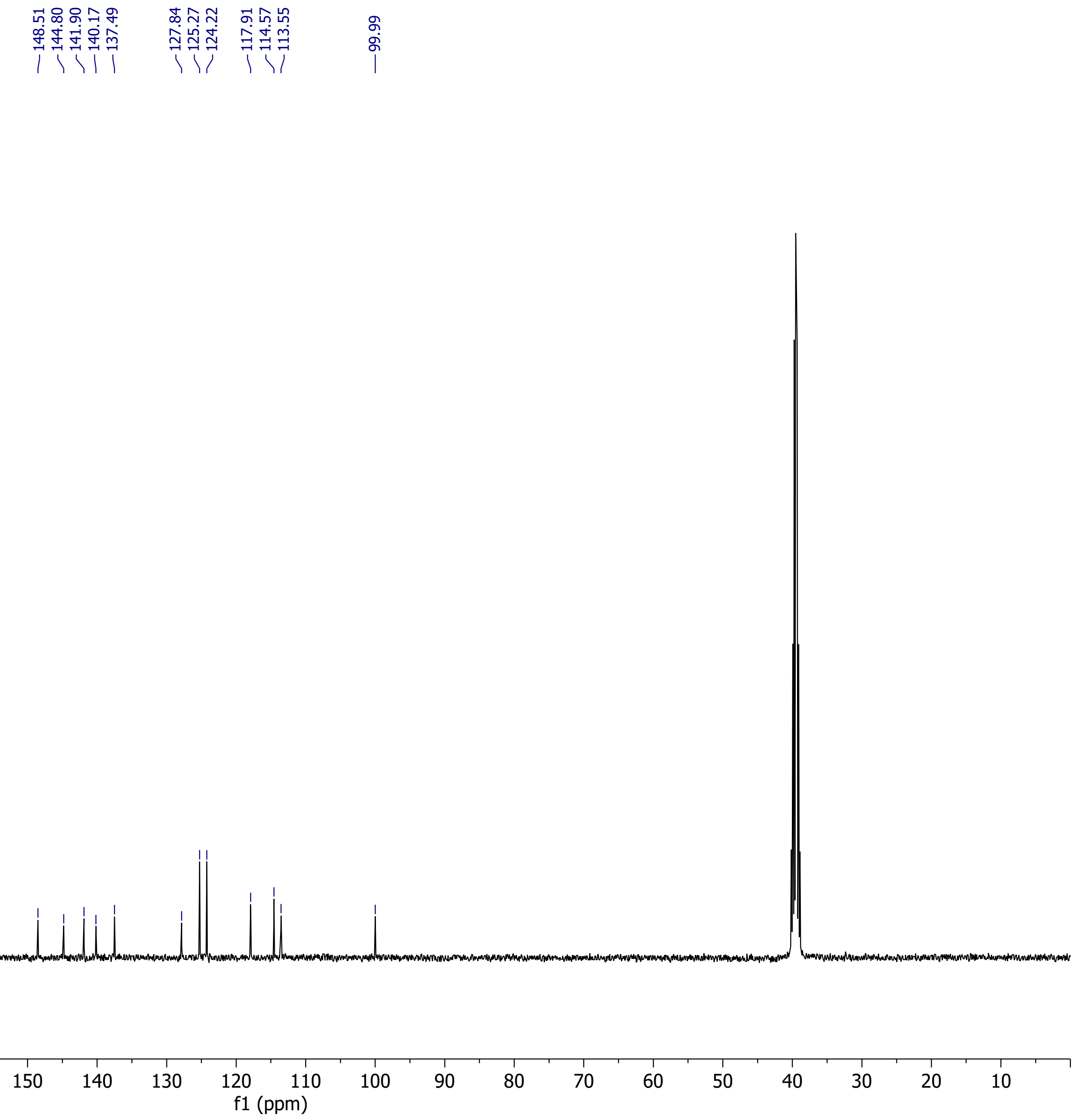

^{1}H NMR (400 MHz, $CDCl_3$)

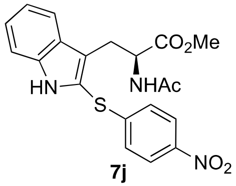

7j

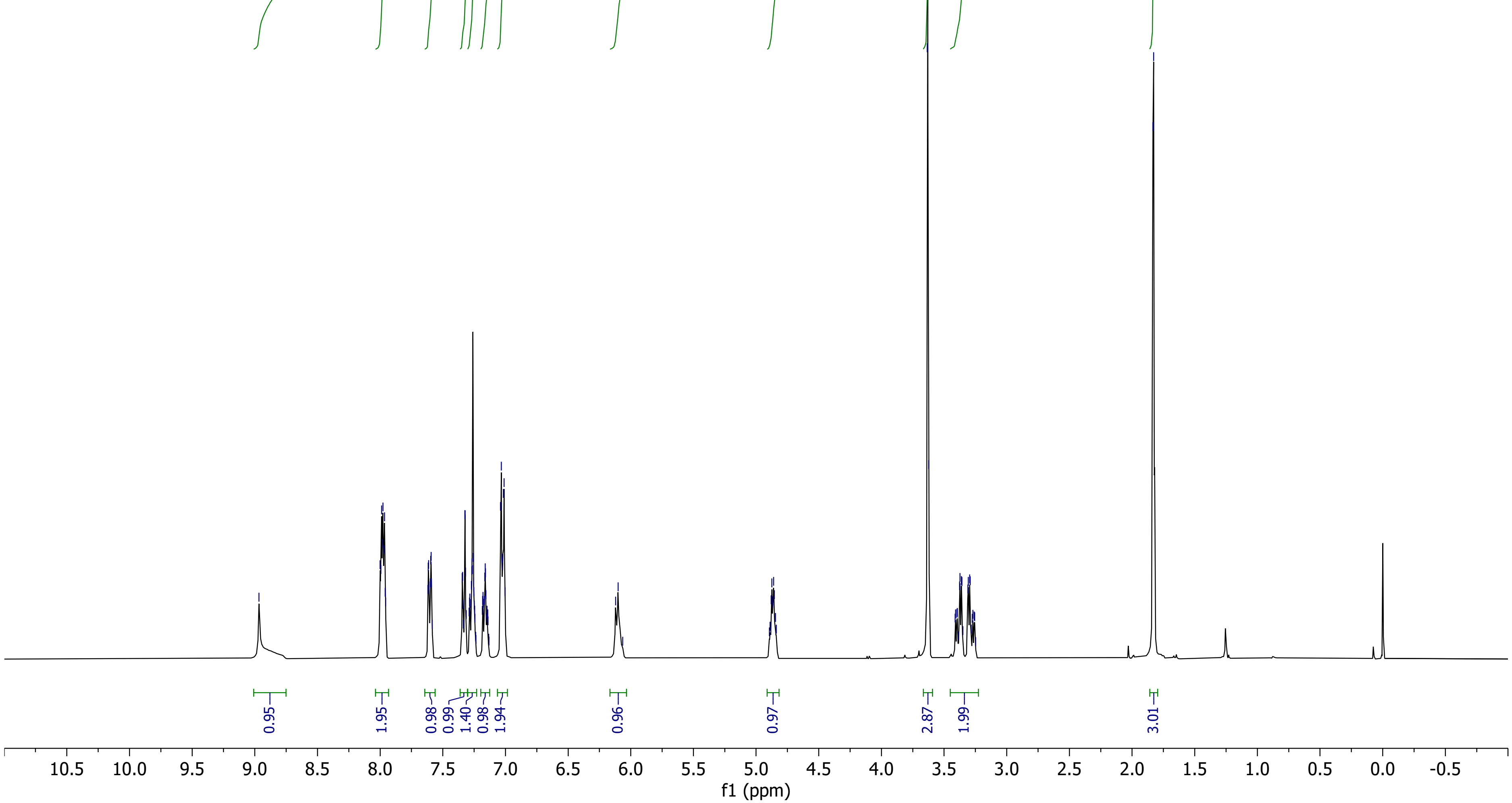

$^{13}C\{^1H\}$ NMR (101 MHz, $CDCl_3$)

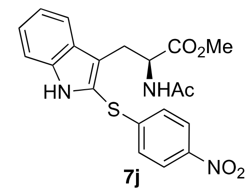

7j

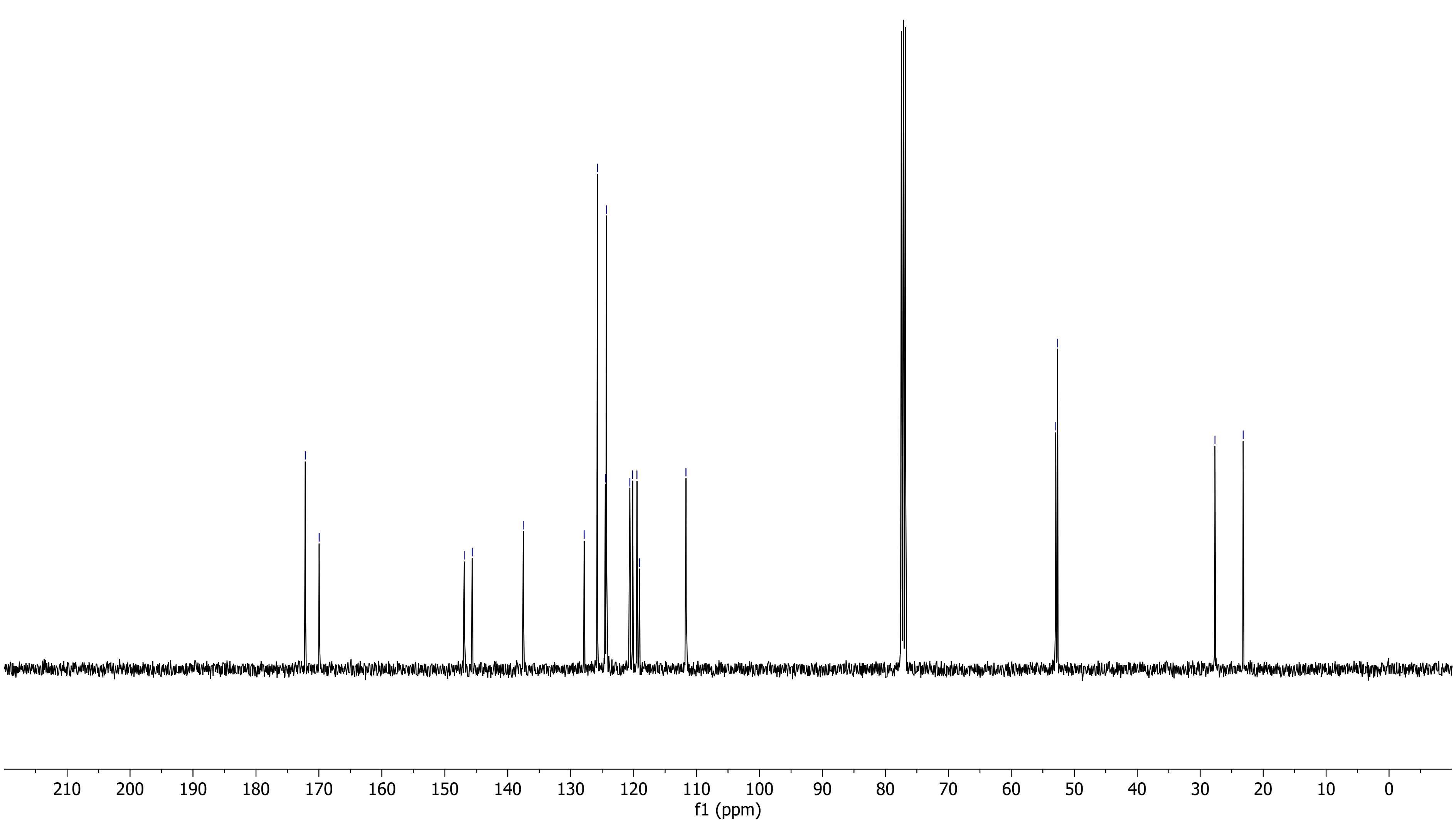

Supplement: Supplementary file 1 — jo5c00250_si_001.pdf [file jo5c00250_si_001.pdf]
